# Randomized phase III trial evaluating motivational interviewing and text interventions to optimize adherence to breast cancer endocrine therapy (Alliance A191901): the GETSET protocol

**DOI:** 10.1186/s13063-023-07672-8

**Published:** 2023-10-12

**Authors:** Joannie Ivory, Stephanie B. Wheeler, Sarah Drier, Heather Gunn, David Zahrieh, Electra Paskett, Michelle Naughton, Rachel Wills, Kayla Swetel, Selina Chow, Katherine Reeder-Hayes

**Affiliations:** 1 University of North Carolina at Chapel Hill, Chapel Hill, NC USA; 2 Chapel Hill, USA; 3 Alliance Statistics and Data Management Center, Mayo Clinic, Rochester, MN USA; 4 Rochester, USA; 5 The Ohio State University, Columbus, OH USA; 6 Columbus, USA; 7 University of Chicago, Chicago, IL USA; 8 Chicago, USA; 9 Alliance Operations Office, Chicago, IL USA

**Keywords:** Breast cancer, Endocrine therapy, Adherence, Clinical trials, Diversity, Health services research, Medical research methods

## Abstract

**Background:**

Hormone receptor-positive (HR +) breast cancer is the most common type of breast cancer in the USA but has excellent long-term outcomes in recent decades, in part due to effective oral endocrine therapy (ET). ET medications are typically prescribed for 5 to 10 years, depending on the risk of recurrence, and must be taken daily. One limiting factor to ET efficacy is nonadherence, with high-risk groups for nonadherence including younger women and Black women.

**Methods:**

The Alliance for Clinical Trials in Oncology (Alliance) trial A191901 is an ongoing, four-arm (text message reminder (TMR), motivational interviewing (MI), TMR plus MI, or enhanced usual care) randomized clinical trial that tests the efficacy and effect of two interventions (TMR and/or MI) on improved ET adherence, patient-reported outcomes (PROs), and resource use requirements among HR + breast cancer survivors. Participants are randomized in a 1:1:1:1 ratio to the four arms. With an assumed loss to follow-up of approximately 11%, we plan to recruit 1180 participants. Randomization is stratified based on age and race to ensure balance between the arms, and we oversample younger and Black women, with each group representing 30% of the study population. Participants randomized to an intervention will actively participate in the intervention for 9 months, and all participants will be followed for adherence data and PRO endpoints, through the use of the Pillsy cap medication event monitoring system and Alliance ePRO survey app (i.e., Patient Cloud). The primary analysis will compare Pillsy-measured ET adherence among study arms at 12 months.

**Discussion:**

This multisite study will not only define strategies to improve adherence to breast cancer oral therapies, but it will also potentially support strategies in large cooperative research groups that can increase delivery and tolerability of ET, involve diverse patient populations in clinical research, and engage patients effectively in interventional studies, using remote and cost-effective delivery methods.

**Trial registration:**

Clinicaltrials.gov NCT04379570. Registered on 7 May 2020.

**Supplementary Information:**

The online version contains supplementary material available at 10.1186/s13063-023-07672-8.

## Background and study rationale

Hormone receptor-positive (HR +) breast cancer is a significant public health problem, with an estimated 180,000 new cases diagnosed among women in the USA annually [1]. Survival rates for HR + breast cancer have steadily improved over the past three decades, in part, due to the introduction of adjuvant endocrine therapy (ET) in the early 1990s. Taken as a once-daily oral medication, ET has proven to be a dramatically efficacious targeted therapy, reducing 10-year risk of recurrence by 47% following 5 years of tamoxifen [2], with further incremental benefits from the newer aromatase inhibitor drugs and longer treatment courses of up to 10 years [3, 4]. Currently, the standard of care for early HR + breast cancer is to treat with ET for 5 to 10 years.

Despite its therapeutic success, nonadherence to ET is a widespread problem. Several recent studies have shown that patients struggle with both early discontinuation and suboptimal dosing, collectively termed “nonadherence” [5]. Hershman and colleagues reported that only 49% of patients in a large commercial insurance cohort took ET for the entire recommended duration at the optimal schedule [6]. In Medicaid and Medicare populations, adherence difficulties emerge even earlier with an estimated 36% nonadherent in the first year and 50% non-adherent by year four [7, 8]. ET non-adherence has been linked to significant decrements in cancer-specific and overall survival in observational studies [9, 10]. In addition, clinical trials comparing different durations of tamoxifen have shown higher recurrence rates for shorter durations of therapy [11].

Many of the factors associated with ET non-adherence are more prominent for younger and Black women and represent potential points of intervention to improve patient care. Factors associated with ET nonadherence include higher side effect burden [12, 13], lower health-related quality of life at baseline, and while taking ET [12–16], lower self-efficacy [17], and poor satisfaction with decision-making and with treatment [13, 18], all of which appear to be more prevalent issues among Black women and younger women [13, 19, 20]. Attitudes and beliefs associated with non-adherence include a woman’s belief that her risk of recurrence is low and that the risk will not change if ET is discontinued [13, 20]. Our team previously found that these beliefs are more common among Black breast cancer survivors and that adjusting for such beliefs attenuates the effect of side-effect burden on non-adherence [13]. In addition, many patients report that they forget to take ET in various situations [13, 18]. Structural factors associated with non-adherence include logistical challenges accessing refills and higher co-payments [21]; as such, longer refill intervals and Medicare Part D low-income subsidies and other copayment assistance may protect against non-adherence [13, 22].

Although modifiable factors affecting ET adherence are well-documented, few effective interventions have been reported to date to address the widespread problem of ET non-adherence in breast cancer [23]. Development of such interventions faces multiple challenges. Effective designs must address multifaceted and cumulative barriers across a wide array of unrelated domains, from forgetfulness and perceptions of risk/benefit tradeoffs to side effect concerns and self-efficacy [6, 19, 21, 24–26]. Due to the large number of patients potentially affected, and the competing demands of busy oncology clinics, feasible designs must find ways to reach patients outside the clinical encounter, and to connect with high-risk groups, including younger (< 50 years old) breast cancer survivors and Black patients. Finally, if health system stakeholders are to adopt ET adherence interventions on a large scale, data regarding the resources required to implement and value of the intervention must be provided.

Successful strategies for medication adherence in non-cancer populations may be adaptable to endocrine therapy delivery. In cardiovascular disease, multi-faceted interventions that address a variety of barriers appear to be most effective [27]. Motivational interviewing (MI), a lay counseling technique in which trained personnel help patients identify their most salient barriers, facilitators, and preferred strategies [28, 29], improves adherence in HIV and other chronic diseases and can be offered remotely [29–32]. Alternatively, interventions leveraging mobile technology through “smart” devices may offer opportunities to reach more patients remotely in an efficient manner; specifically, text message reminder (TMR) systems have been linked to improved adherence in cardiovascular disease patients [33, 34]. However, the optimal combination of remotely delivered medication support strategies for oncology patients generally, and for ET patients specifically, remains unknown.

To evaluate the optimal strategy for remote support of ET adherence, we designed the Guiding Endocrine Therapy Success through Empowerment and Technology (GET SET) trial (Alliance for Clinical Trials in Oncology [Alliance] A191901). GET SET is conducted within the National Clinical Trials Network (NCTN) in the USA, and all participating sites must be one of the following: (1) a lead academic participating site (LAPS), (2) a NCTN main member site, or (3) a member of the NCI Community Oncology Research Program (NCORP) through the Oncology Patient Enrollment Network (OPEN). Sites self-select to participate in trials, provided the institution obtains institutional review board (IRB) approval and meets all the credentialing requirements included in the specific protocol. In addition, the Alliance membership committee evaluates the site’s institutional resources for clinical trials, prior clinical research experience, patient population, prior institutional performance evaluation metrics, audit results, and other regulatory considerations. The study evaluates the impact of smartphone-based text messaging reminders and telephone-delivered motivational interviewing on ET adherence, with a prospective randomized design and sampling framework that permit full evaluation of effects in young and minority populations, as well as resource use and value. The trial leverages collaborations between the University of North Carolina at Chapel Hill, The Ohio State University, and the infrastructure of the Alliance research network, with a particular focus on network partners who engage minority and younger patients. The study opened to accrual on February 4, 2021, and as of September 15, 2023, has accrued 1024 of 1180 planned patients. In this manuscript, we outline the study design and protocol considerations of the ongoing study.

## Methods

### Objectives

Guiding Endocrine Therapy Success through Empowerment and Technology (GETSET) is a randomized controlled trial designed to test the efficacy of two interventions compared to usual care: (1) a text messaging reminder (TMR) system and (2) telephone-based motivational interviewing (MI). Patients are randomized into one of four arms: TMR, MI, combination of TMR and MI, or enhanced usual care. The primary objective is to compare adherence to oral endocrine therapy at 12 months post-enrollment among the 4 arms. Secondary and exploratory objectives will describe (1) effects of the interventions on health-related quality of life and other patient-reported outcomes, (2) costs and cost-effectiveness of the interventions and other implementation outcomes, and (3) differences in disease-free survival across arms.

### Study design

Target enrollment is 1180 patients. The study population will include both 30% Black women and 30% patients under age 50, which are both groups at high risk for non-adherence. An efficient study design will be used to compare each intervention and their combination to usual care, in terms of the following: ET adherence assessed by electronic pill cap (Aim 1), patient-reported outcomes including medication self-efficacy, health-related quality of life, cancer worry, knowledge and attitudes about ET (Aim 2), and relative resource use (cost) and value of interventions (cost-effectiveness) (Aim 3). We will also evaluate whether the intervention effects on outcomes differ by race and age. We used the SPIRIT reporting guidelines to ensure we have included all pertinent clinical trial protocol information in this manuscript [35] and have included the checklist as a supplement.

### Research ethics and dissemination

The trial protocol, template informed consent forms, participant-facing materials, and recruitment materials were reviewed and approved by the NCI Division of Cancer Prevention (DCP), Alliance for Clinical Trials in Oncology’s Cancer Control Program (CCP) protocol committee, and the NCI Central Institutional Review Board (CIRB) on May 28, 2020. The trial protocol and study activities are reviewed annually by the NCI CIRB, and progress reports are submitted annually to the NCI DCP. All subsequent protocol amendments and modifications are subject to approval from the Alliance for Clinical Trials in Oncology’s Cancer Control Program (CCP), the NCI Community Oncology Research Program (NCORP), and NCI CIRB. Protocol modifications are communicated to site research staff via memorandums from the Alliance for Clinical Trials in Oncology and via monthly site staff webinars.

The study’s data will be monitored by the Alliance Data Safety and Monitoring Board (DSMB), an NCI-approved functioning body, twice per year. The DSMB follows the Alliance Policies and Procedures for all randomized phase III trials. The DSMB will review administrative information, accrual, including accrual of Black women and according to age categories, and interim analysis results. All summary findings of the DSMB will be communicated to study investigators by the Alliance.

All participant and study-related information will be stored in secure databases with password-protected access given only to members of the trial team. All study data will be coded with a unique study identification number, assigned at registration, to maintain participant confidentiality, and will be stored separately from linking participant information. Any patient-level data shared with researchers outside of the trial team for subsequent research will limit the amount of identifiable data, in accordance with informed consent forms and HIPAA authorizations obtained for trial participation.

Adverse events beyond those related to usual care are not anticipated and, as such, are not being collected or reported to the DSMB. If participants feel distressed due to participation in this minimal-risk behavioral intervention, they may choose not to continue or finish the allocated intervention and/or speak to the site staff. Participants experiencing any physical or psychological complications related to taking endocrine therapy should discuss these concerns with their treating physician.

The final dataset is the property of Alliance for Clinical Trials in Oncology which adheres to the NCI’s requirements for data accessibility. It is anticipated that individual-level data, whether de-identified or otherwise, will be available on the National Clinical Trials Network (NCTN) Data Archive or Project Data Sphere within 15 months after publication of the primary analysis. If the desired data are not available on either of these resources, the data will be made available to individuals upon request through the Alliance website. Trial results will also be disseminated via professional meeting presentations, research publications, and the clinicaltrials.gov website, which must be approved by the Alliance publications committee. The study’s website (https://getsetstudy.org) will be used to disseminate key findings to study participants and other interested parties in layperson terms.

### Patient selection

The trial enrolls women ages 18 + years whose initial diagnosis of stage I–III, HR + , HER2-neu negative, invasive breast cancer occurred within 18 months prior to trial enrollment. Participants must have received cancer-directed surgery and completed all other planned adjuvant therapy, except breast reconstruction. However, the extent of prior adjuvant therapy is not mandated. The trial employs a new user design, meaning that eligible patients have either initiated their first ET medication within the 6 months prior to enrollment, or have received a prescription with stated intent to initiate within 6 weeks post-enrollment, and have no medical contraindications to ET (e.g., existing, or planned pregnancy). Participants are required to use a smartphone for study participation; for participants who do not have a smartphone at baseline, phones and services are provided. Because the medication event monitoring (pill cap) system and the interventions themselves were designed to measure and address adherence to a single once-daily oral drug, patients whose ongoing therapy includes additional drugs such as a CDK 4/6 inhibitor, as well as those taking endocrine therapy on nonstandard schedules (e.g., tamoxifen 10 mg twice daily), are excluded; however, premenopausal patients treated concurrently with an injected gonadotropin-releasing hormone agonist (e.g., leuprolide) are eligible.

### Site requirements

Participating sites must be a member of the NCI Central Institutional Review Board (CIRB) and accept NCI CIRB review to activate new sites. A protocol-specific training is provided to all sites prior to local study activation. The training included information regarding study protocol; key eligibility criteria; intervention activities for each study arm; study procedures for site staff specific to the Pillsy medication event monitoring system, the Medidata ePRO app, and the study-provided smartphones; and brief strategies for troubleshooting study technology. Time was provided at the end of the training for questions from site staff regarding the protocol and/or study operating procedures. All institutions are audited at least once every 36 months to assure performance standards are being met and as an educational experience for the new investigators and their staff.

### Patient registration requirements

**Fig. 1:**
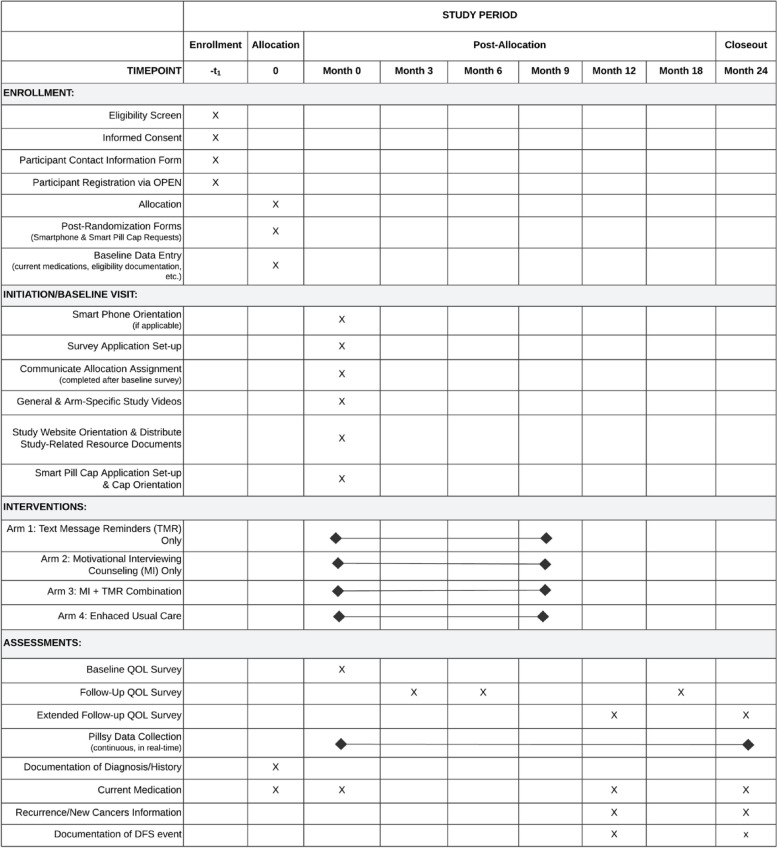
Schedule of enrollment, interventions, and assessments

Participants are recruited by clinical research coordinators at participating sites, including the main member and NCI Community Oncology Research Program (NCORP) network sites of the Alliance for Clinical Trials in Oncology. Local site coordinators complete informed consent procedures, assess the need for a study-provided smartphone, and collect study-required contact information for intervention administration. Following consent, site coordinators complete registration and randomization procedures via a web-based registration system, which accounts for the stratification factors: age and self-identified race. Participants are randomized in a 1:1:1:1 ratio to participate in one of the four study arms. Randomization is stratified on the basis of age, in years (< 50; ≥ 50), and race (Black women; non-Black women). Participants return to the clinic for a required, in-person baseline visit to set up the Patient Cloud (ePRO) and Pillsy smartphone applications and receive other arm-specific resources. The schedule of enrollment, interventions, and assessment can be found in Fig. 1.

### Study interventions

**Fig. 2:**
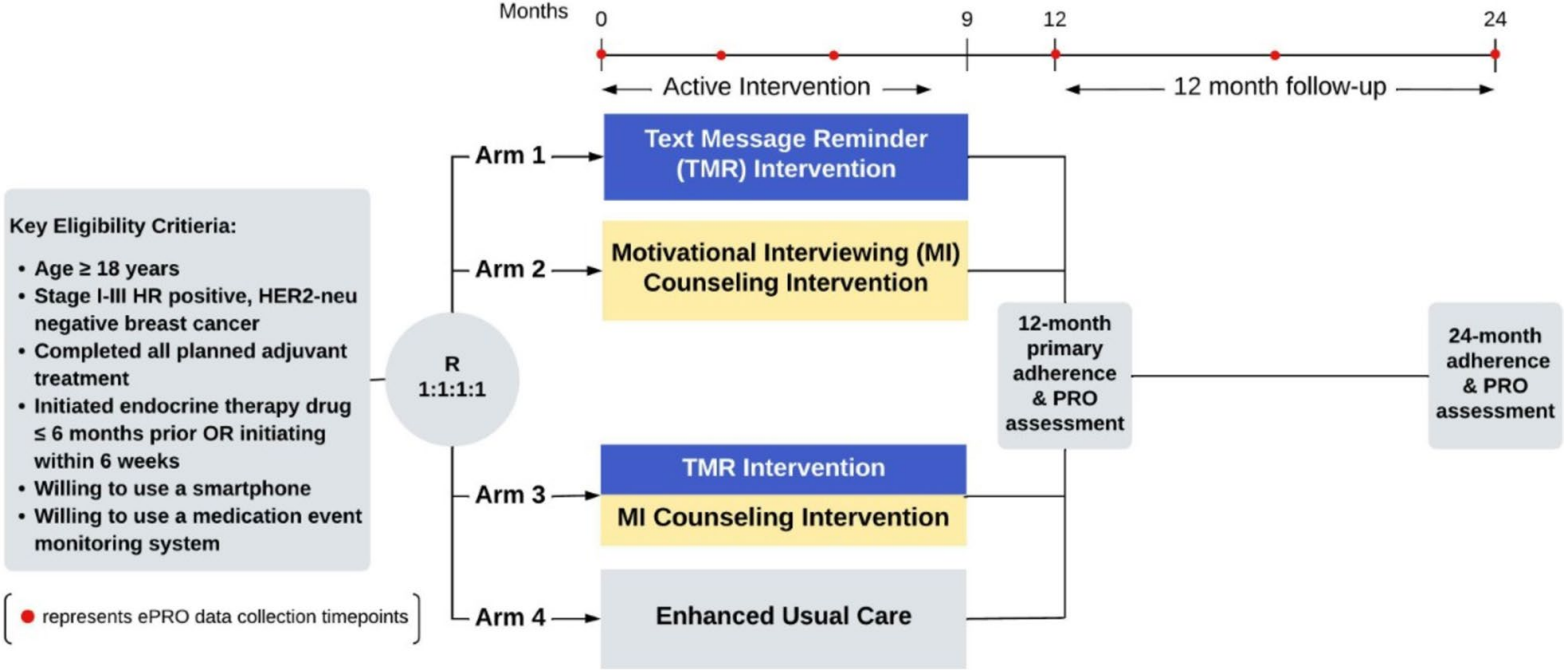
Study schema depicting timing of intervention activities and outcome assessment

All study intervention components are delivered centrally by one of the coordinating centers and delivered over a 9-month period. Participants who have not started endocrine therapy medication at the time of registration must initiate the drug within 6 weeks after randomization and begin their allocated intervention within 8 weeks after randomization. Allocated intervention may be stopped prior to completion at the patient’s request or disease progression. The study schema can be found in Fig. 2.

#### Text message reminder (TMR) component (arms 1 and 3)

Participants receive two types of messages to address adherence behavior: (1) daily text message reminders to take their endocrine therapy medication and (2) monthly interactive adherence check-ins, which help to provide accountability and resolve specific barriers through the provision of pre-set motivational responses. Daily text reminders are delivered from a library of 62 distinct messages designed to encourage medication adherence in a positive manner; text reminders focus on three behaviors: initiation, continuation, and adherence to the prescribed dose, as appropriate. Monthly adherence check-ins ask participants “Over the past 7 days, on how many days did you take your ET [endocrine therapy medication]?” Participants respond with a number 0–7, and any response of 6 or greater triggers a positive message that encourages patients to keep taking the medication. Responses of less than 6 are followed by questions about reasons for missed doses and tips for improving adherence. Participants who report at least 2 consecutive months of adherence problems in response to the monthly adherence check-ins will receive additional texts regarding contact with their physicians/health care team and refer participants back to the text message intervention informational handout provided at the beginning of the study participation. All of the daily text message reminders and interactive responses are based on PACE principles (Presenting detailed information, Asking questions, Checking your understanding, and Expressing concerns), with the most common response being to talk directly with their health care team [36, 37].

#### Motivational interviewing (MI) counseling component (arms 2 and 3)

Participants receive 5 telephone-based behavioral counseling sessions utilizing motivational interviewing to target ET initiation, adherence, and persistence. Counseling sessions last anywhere from 30 to 90 min, are conducted by a trained MI counselor in a one-on-one format, and are tailored to individual participant needs, circumstances, and/or values. Each session follows a standardized, semi-structured guide to help develop motivation and skills to aid adherence. Later sessions utilize a toolbox approach, allowing participants to choose from a list of available activities and topics. This format provides flexibility in how the intervention is delivered to meet individual needs and tailors the intervention to meet the needs of specific ethnic, socio-economic, and other specific problems. A counseling workbook is used to facilitate conversation and support participant adherence between sessions. Counselors, located centrally at a coordinating center, attend 3 days of MI-focused training that includes a combination of didactic sessions, video demonstrations, and practice sessions with role-play to develop and enhance skills. The training also provides detailed information about endocrine therapy, cultural sensitivity and competency, barriers women taking endocrine therapy often face, and potential practical solutions to these barriers.

#### Enhanced usual care component (arms 1–4)

In addition to routine breast cancer care received by each participant by their local oncology care team (“usual care”), participants in all arms receive general health education information for breast cancer survivors, delivered via the CHART tool’s interactive modules on the study website. The CHART behavioral assessment tool aims to strengthen behavioral interventions and collect health behavior data by assessing, improving, and modifying participants’ behavioral risk factors. Modules provided to study participants include physical activity, eating habits, weight, emotional health, tobacco use, alcohol use, sleep habits, and balance.

### Study measures

Baseline sociodemographic data are collected on all participants using the Alliance for Clinical Trials in Oncology patient questionnaire. Key components include self-identified race, gender, ethnicity and language preferences, information on income, household size, insurance, employment status, and selected personal and family health history (Table 1).

**Table 1:** Participant study measures

Domains and Measures	# items	Baseline	Months 3, 6, 18	Month 12	Month 24
**Socio-demographics:**					
**Alliance Patient Questionnaire (select items):** Age, gender, ethnicity, race, language, education, marital status, health insurance, household size, income, employment status	13 (9 items at Months 12, 24)	X		X • Marital status • Health insurance • Household size/income • Employment	X • Marital status • Health insurance • Household size/income • Employment
**Medical and Family History**:					
Medical and Family History	7	X		X	X
**Quality of Life/ Psychosocial:**					
PROMIS Global Health Questionnaire	10	X		X	X
Breast Cancer Prevention Trial (BCPT) Symptom Questionnaire	25	X		X	X
PROMIS Depression Short Form 8a	8	X		X	X
Concerns About Cancer Recurrence (CARS); overall concerns subscale only	4	X		X	X
Perceived Stress Scale	10	X		X	
Modified Medical Outcomes Study Social Support (mMOS-SS) Questionnaire	8	X		X	
**ET Medication Use:**					
Medication Understanding and Use Self-Efficacy Scale (MUSE)	8	X	X	X	X
Self-reported ET adherence and persistence	17	X	X	X	X
**Intervention Acceptability**:					
Intervention Evaluation: Acceptability, Appropriateness, Ease of Use	15			X	
Was It Worth It (WIWI) Questionnaire for Alliance clinical trial participation	5			X	
**TOTAL ITEMS:**	130	110	25	126	88
**TOTAL ESTIMATED TIME:**		37	10	42	30

At baseline and key follow-up timepoints, electronically collected patient-reported outcome (ePRO) data are collected using the following validated instruments: PROMIS global health and depression questionnaires for health-related quality of life [38], Breast Cancer Prevention Trial (BCPT) survey for breast cancer-related physical symptoms [39], Concerns About Recurrence scale for cancer worry [40], Perceived Stress Scale [41, 42], Modified Medical Outcomes Study Social Support Survey (MMOS-SS) [43], and Medication Use Self Efficacy (MUSE) scale [44]. Self-reported ET adherence behaviors are also collected using the Self-Reported Endocrine Therapy Adherence and Persistence (SETAP) instrument used by Drs Reeder-Hayes and Wheeler in multiple prior published studies. The majority of ePRO measures are collected at baseline and 12 and 24 months; the MUSE scale and SETAP are also collected at 3 and 6 months. All patient-reported data are obtained via the Alliance ePRO app which is downloaded to patient’s phones at enrollment and stored along with sociodemographic data and coordinator-entered baseline data in the Medidata RAVE system. This ePRO data collection enables the remote nature of the study, with only the baseline visit being a required face-to-face interaction between site staff and the participant. Participants receive incentive gift cards after completion of surveys at each timepoint.

Data for the primary endpoint of endocrine therapy adherence are collected automatically from participants using an electronic medication event monitoring system (Pillsy Inc, Seattle, WA). An electronic cap is mailed from the coordinating center and distributed to each participant at the baseline visit. Daily adherence data are collected beginning from the first day following electronic pill cap activation on which the cap records an electronic “open” event for 24 months. Adherence is assessed at 12 months as a percentage of days covered, defined as the number of days with at least one “open” event divided by the total number of days in the observation period. Electronic adherence data are returned to Pillsy continuously via Bluetooth connection to the patient’s phone; data for all participants are returned to the study team on a monthly basis via secure data feed and stored in Medidata RAVE. Since the pill cap stores data continuously, periodic disconnections of the cap from its Bluetooth connection do not threaten data integrity if the cap is eventually re-connected. Participants are reminded at four pre-set timepoints of participation to place their cap near their phone and verify connectivity. With the exception of these reminders, data collection is completely passive on the participant’s part and the cap does not issue sounds or other reminders to prompt medication taking.

The amount of missing data in Alliance clinical trials has been minimized, typically to less than 5%, due to a long history of targeted approaches. If the proportion of missing patient information is larger than 5%, we will examine the missingness mechanism by modeling indicators of missing values as a function of baseline patient characteristics, observed outcomes, and missingness of outcomes. Based on this diagnostic exercise, we will determine whether we can enhance the analyses by incorporating certain covariates or whether we need to explore more advanced methods.

### Sample size

Enhanced usual care adherence at 12 months is assumed to be 69%, based on our preliminary studies of pharmacy refill data from claims in Medicare, Medicaid, and private insurance populations, using weighted estimates of 12-month adherence among Black women < 50 years old, non-Black women < 50 years old, Black women ≥ 50 years old, and non-Black women ≥ 50 years old. We hypothesize 81% adherence for the TMR-only and MI-only intervention arms based on preliminary data from the GETSET MI counseling pilot intervention [37, 45]. To maintain a primary family-wise two-sided type I error rate of* α* = 0.05, the two chi-square tests will be conducted with nominal *α* = 0.025. At a 2.5% two-sided significance level, the study will have at least 80% power to detect differences in adherence (81% in single component intervention arms vs 69% in enhanced usual care) if 263 evaluable patients are randomized each to the TMR-only, MI-only, and enhanced usual care arms. Because we anticipate ineligible and loss to follow-up of approximately 11%, consistent with other clinical trials of cancer survivors, we will over-recruit, rendering a recruitment target of 1180 randomized patients total (295 per arm). Power Analysis and Sample Size (PASS) software (PASS v15, NCSS, Kaysville, UT) was used to conduct the power analysis.

Young age and self-reported Black race have been shown to be risk factors for low ET adherence [13, 19, 20]. As such, the study is designed to accrue 30% (or 354 of 1180) of patients who self-identify as Black as well as 30% who are < 50 years old at time of recruitment. Additionally, the ideal case is for race and age to be uncorrelated with one another so that they have independent effects on the outcome of interest. To achieve this, we have defined distinct accrual targets for four non-overlapping groups (i.e., Black women < 50 years old, non-Black women < 50 years old, Black women ≥ 50 years old, and non-Black women ≥ 50 years old) and close accrual for each group independently. The target accrual numbers for each group are shown in Table 2.

**Table 2:** Accrual targets by race and age strata

Age (years)	Black	Non-Black	Total
< 50	106	248	354
≥ 50	248	578	826
Total	354	826	1180

### Randomization and strategic staged closure

Patients are randomized using a dynamic allocation system based on the method by Pocock and Simon [46] that continuously balances the allocation of participants across the four treatment groups and race (black vs. non-Black) and age (≥ 50 vs. < 50) stratification factors, regardless of which race and age strata remain open at the time of randomization. To avoid the algorithm being deterministic, a level of randomness is added to the algorithm such that patients are assigned to the arm that leads to more imbalance 10% of the time. Participants are consented and enrolled to the trial by local site coordinators via the Oncology Patient Enrollment Network (OPEN), a web-based registration system integrated with the regulatory and roster data as well as the lead protocol organization (LPO) registration/randomization system and the Theradex Interactive Web Response System (IWRS) which is used for retrieval of the patient registration/randomization assignment. Using these systems, participant assignment to study arms is automatically completed upon enrollment. Neither participant nor study staff are blinded to intervention allocation since this would not be feasible in a behavioral intervention. However, the primary outcome assessment is measured objectively using a medication event monitoring cap, and investigators are effectively blinded to primary endpoint data since data are collected and centrally managed externally by Alliance’s RAVE system and analysts. To achieve target numbers of patients in each race and age stratum as laid out in the statistical plan, while still accruing the overall study as quickly as possible, we use a process we describe as “strategic staged closure”. Strategic staged closure is the process of monitoring accruals according to the study’s pre-specified sub-group strata (in this case, younger Black, older Black, younger non-Black and older non-Black) and closing each stratum individually as it approaches its target accrual. The study chairs meet monthly throughout the study with the statistical team to review the pace and cumulative numbers of patients accrued in each stratum. As numbers approach the desired target for each stratum, a memorandum of impending closure is prepared for distribution to participating sites setting a date for closure estimated from the recent pace of accruals in that sub-group. We aim to notify sites of impending closure approximately 2 weeks in advance to allow any patients in active screening to consent to the study before stratum closure.

## Outcomes

### Primary endpoint

The primary endpoint is ET adherence at 12 months post-randomization as measured by electronic pill monitoring cap (Pillsy-reported). Patients will be labeled as adherent if the proportion of days covered by any ET medication is at or above 80% (i.e., taking ET at least 292 of 365 days). Women who do not take ET for at least 292 days will be deemed non-adherent at 12 months. The primary analysis will be based on a modified intention-to-treat population (mITT), defined as all women who signed a consent form, met the eligibility criteria, were randomized, and took ET on at least one occasion; furthermore, these women will be analyzed in the arms to which they were randomized. For the primary analysis, two comparisons will be made using two chi-squared tests: TMR-only versus enhanced usual care and MI-only versus enhanced usual care. For each comparison, statistical significance will be assessed at the 2.5% level. Should either TMR-only or MI-only arms achieve statistical significance at its allocated α-level (or accumulated unused α-levels if both achieve statistical significance), then the TMR + MI vs enhanced usual care comparison will be assessed and the unused α-level passed down and applied to determine statistical significance. In this way, the type I error rate applied to the primary family of endpoints can be controlled at the 5% level.

As a sensitivity analysis, we will assess if the intervention effects on ET adherence differ by the stratification factors of age and race. Therefore, logistic regression is planned to model the binary measure of adherence at 12 months separately for each intervention vs enhanced usual care comparison so that three separate logistic regression models will be estimated. Covariates included in each logistic regression model will be age (< 50 years; ≥ 50 year), race (Black women; non-Black women), intervention arm assignment, and the interaction terms between each stratification factor (age and race) and intervention arm assignment to ascertain whether the magnitude of the intervention effect is different across the levels of age and race. Statistical significance of the interaction terms will be assessed at the 10% level with the likelihood ratio test comparing models with and without the interaction terms.

### Exploratory endpoints

In addition to the primary endpoint, we will observe 13 exploratory endpoints. These include self-reported adherence, patient-reported MUSE score, PROMIS physical health subscale score, PROMIS mental health subscale score, PROMIS depression short form, Concerns of Recurrence scale score, Perceived Stress scale score, Medical Outcomes Study Social Support Questionnaire score, disease-free survival including ductal carcinoma in situ, cost, intervention acceptability, research study satisfaction, and accrual pattern of Black and younger women for the first half of patients enrolled to the study (i.e., 590 patients). All of these endpoints are hypothesis-generating, requiring additional examination in independent study.

### Analysis of accrual patterns

At 50% enrollment, a pre-planned analysis of accrual patterns will be triggered to examine site-level factors associated with accrual of a higher proportion of sub-groups of particular interest (Black and younger participants). Site-level factors of interest include sites’ volume of accrual to previous Alliance protocols, membership type, geographic region, and (for race analysis) racial composition of the neighborhood in which the site is located. Data from this analysis will be used to select Alliance sites not yet activated to the study for targeted outreach and invitation to open the study if overall enrollment is lagging, and to inform study design and site selection for future Alliance protocols seeking to enroll higher-minority samples.

### Process evaluation

The goal of the process evaluation is to explore the implementation of GETSET to inform future integration into cancer care delivery. The process evaluation includes data about cost as well as the facilitators and barriers to implementing remotely delivered medication support interventions like TMR and MI, from both the patient participant and practice staff perspectives.

*Cost analyses* are focused on ascertaining the overall cost, cost-effectiveness, and budget impact associated with delivering the TMR and MI interventions from the health system/practice (primary) and societal (secondary) perspective. Total incremental per-patient cost of MI-only, TMR-only, and TMR + MI interventions over the first 12 months, and the incremental cost of MI, TMR, and MI + TMR per 1 percentage point improvement in PDC at 12 months, compared to usual care will be reported. We are interested in estimating the total cost (sum of fixed and variable costs) in current US dollars per person recruited to the MI, TMR, or MI + TMR arms, relative to usual care. We will document all fixed and variable costs associated with delivering the interventions (above and beyond usual care) from the health system/practice perspective, including personnel costs associated with identifying eligible patients, MI counseling, TMR message delivery system setup and monitoring, and educational materials printing/mailing costs. For MI, allowable costs will include all counselor time required to schedule, complete, and document MI sessions. For TMR, allowable costs will include staff time to support the text-messaging platform, provision of phone service for 12 months to participants without text-messaging-capable phones, and staff time to troubleshoot participant problems with phones or other technical aspects of the TMR intervention. Patient time required to complete surveys and research coordinator time for study-related procedures will not be included. Cost data will be collected from a variety of sources, including administrative invoicing records and receipts (e.g., smart phones and TMR programming costs), counselor call logs in REDCap (including time spent contacting and counseling patients), and personnel time logs and wages. Cost data from all sources will be collated, deduplicated, and sorted in a spreadsheet and analyzed and reported descriptively.

*Participant process assessments* will be collected via surveys about ease of use, acceptability, responsiveness to needs, perceived benefits, and challenges associated with intervention participation. At the end of the study, participants are asked to evaluate intervention acceptability using the Acceptability of Intervention and Intervention Appropriateness Measures of Weiner et al. [47] as well as an ease-of-use questionnaire developed specifically for this study and the Alliance “Was It Worth It” questionnaire regarding the overall trial participation. Data from these patient surveys will be summarized descriptively, overall, and by race and age. Participants in the TMR component will also be asked to complete a brief survey about the ease of identifying and answering the text messages and responses, the length of time receiving text message reminders, and other feedback about the TMR components. The survey will be texted directly to participants at the same time as their monthly questions (month 9). Any participant randomized to the TMR component who receives an iPhone from The Ohio State University will be texted a brief evaluation survey at month 12 about training provided, the ease of using the iPhone, and benefits of having a smartphone.

*Practice and research staff process assessments* will be collected via key informant interviews exploring acceptability, fit with workflows, and more. Based on the process evaluation conducted in the counseling-based pilot study, we will use the Proctor Implementation Outcomes Framework to understand staff perceptions of feasibility, fidelity, acceptability, and potential sustainability [48]. We will also use the Consolidated Framework for Implementation Research (CFIR) [49] to clarify determinants of GETSET implementation. To collect these data, we will conduct semi-structured, one-on-one interviews with key informants from participating organizations, including partnering oncologists and other care team members, as well as intervention and research staff, including counselors, clinical coordinators, and technical staff. No interviews will be conducted with study participants. The qualitative interview guides will be tailored to include questions specific to each category of respondent and will address constructs such as intervention features, compatibility with needs and resources, compatibility with internal organizational structure, and process. A semi-structured interview guide can be found in Appendix XVI of the study protocol which is attached as an additional file. Data from these interviews will be transcribed in whole and analyzed thematically using CFIR-derived codebooks.

## Discussion

The Alliance A191901 GETSET trial is innovative because we will evaluate not only the efficacy of the proposed interventions, but also patient-reported outcomes and economic outcomes associated with the interventions within the same research proposal. In addition, we employ stratified sampling to achieve a diverse sample, oversampling both Black women and younger women to ensure adequate representation. This strategy will also inform the design of future clinical trials and efforts to increase enrollment of participants from underrepresented populations. We also use novel approaches to behavioral intervention that can be delivered remotely at scale with low burden to patients and research coordinators, thus appealing to a wider array of patients and research sites than traditional clinical trials. GETSET also represents one of the largest, completely remote behavioral interventions administered within the Alliance cooperative research group, and our hope is to show the feasibility of executing this type of intervention on a large scale.

A temporary suspension of accrual to the trial at all sites began in November 2022, approximately 21 months into recruitment. This temporary suspension was for administrative reasons, and not associated with rate of recruitment, IRB requirements, or adverse events experienced by participants. The vendor supplying the electronic medication event monitoring caps experienced unanticipated problems activating the equipment, and therefore, accrual was temporarily suspended to new patient registration until this issue is resolved. During the suspension, patients previously accrued to the study have continued to follow protocol-specified treatment and testing schedules, as planned, and data collection has continued as per protocol. We anticipate completion of the original target accrual after re-opening, expected in October 2024.

We see multiple potential lessons from this ongoing trial, not only to define strategies that improve adherence to breast cancer oral therapies, but to expand knowledge of how oncology clinical trials can adopt innovative design features that increase sample diversity, maximize accrual, and engage patients effectively in interventional research, using remote and cost-effective delivery methods. We hope to apply multiple design features of the GETSET study to future cooperative group clinical trials to increase the feasibility, efficiency, and diversity of clinical research, including guided over-sampling of patient subgroups of interest through strategic staged closure by subgroup, remote collection of both patient-reported and clinical data, promotion of rapid accrual by offering behavioral support interventions of widespread interest to patients and clinicians, and integration of economic analysis into the evaluation of patient support programs to promote their wider eventual adoption into clinical practice. This study’s attention to studying the process of implementation will also support additional investigation of the “key ingredients” of the intervention, potential adaptation of the intervention, and strategies that may successfully influence adoption and sustainment in clinical practice settings.

## Trial status

Accrual temporarily suspended.

Protocol version number and date: Update 01, June 28, 2022.

Date recruitment began: February 15, 2021.

Approximate date recruitment will be completed: October 2024.

The trial registration details are posted to the clinicaltrials.gov registry and results database.

### Supplementary Information

**Additional file 1.** SPIRIT Checklist for Trials.

**Additional file 2.** Research Study Informed Consent Document.

**Additional file 3. **Optimizing endocrine therapy through motivational interviewing and text interventions.

## Data Availability

There are no results being reported in this publication. The manuscript is a description of the design and methods for a randomized clinical trial, testing endocrine therapy adherence support. The results of the study will be reported after full enrollment and data analysis.
