# Supplementary material for: Randomized phase III trial evaluating motivational interviewing and text interventions to optimize adherence to breast cancer endocrine therapy (Alliance A191901): the GETSET protocol
Source: Trials. 2023 Oct 12;24:664. doi: 10.1186/s13063-023-07672-8 (PMC10568920; doi:10.1186/s13063-023-07672-8)
Supplement: Supplementary file 2 — Additional file 2. Research Study Informed Consent Document. [file 13063_2023_7672_MOESM2_ESM.docx]

Research Study Informed Consent Document

Study Title for Participants: Additional Support Program via Text Messaging and Telephone-based Counseling for Breast Cancer Patients receiving Hormonal Therapy

Official Study Title for Internet Search on <http://www.ClinicalTrials.gov>: Protocol A191901, “Optimizing Endocrine Therapy Through Motivational Interviewing and Text Interventions,” (NCT # 04379570)

Overview and Key Information

What am I being asked to do?

We are asking you to take part in a research study. This study has public funding from the National Cancer Institute (NCI), part of the National Institutes of Health (NIH) in the United States Department of Health and Human Services. We do research studies to try to answer questions about how to prevent, diagnose, and treat diseases like cancer.

We are asking you to take part in this research study because your doctor has prescribed taking an endocrine therapy (ET) medication daily by mouth as part of your treatment for your hormone receptor positive breast cancer.

Taking part in this study is your choice.

You can choose to take part or you can choose not to take part in this study. You also can change your mind at any time. Whatever choice you make, you will not lose access to your medical care or give up any legal rights or benefits.

This document has important information to help you make your choice. Take time to read it. Talk to your doctor, family, or friends about the risks and benefits of taking part in the study. It’s important that you have as much information as you need and that all your questions are answered. See the “Where can I get more information?” section for resources for more clinical trials and general cancer information.

This study is conducted by the Alliance for Clinical Trials in Oncology, a national clinical research group supported by the National Cancer Institute. The Alliance is made up of cancer doctors, health professionals, and laboratory researchers, whose goal is to develop better treatments for cancer, to prevent cancer, to reduce side effects from cancer, and to improve the quality of life of cancer patients.

Why is this study being done?

This study is being done to answer the following question:

Can we increase the number of days that women take their ET medication by adding support programs such as text message reminders, telephone-based counseling, or both?

We are doing this study because we want to find out if using either text message reminders or telephone-based counseling, or both is better or worse than the usual approach for making sure that women take their ET medication as prescribed. This is also called medication adherence.

Medication adherence is how well patients take the medication as prescribed by their doctors and good medical adherence is when patients take medications correctly. Poor medication adherence has been shown to be a serious barrier to effective treatment for hormone receptor positive breast cancer patients.

The usual approach is defined as the medical care most people get while taking ET medication.

What is the usual approach to my medication adherence?

The usual approach for patients who are not in a study is to get basic education about the ET medication from their doctor. This information might be provided by a doctor, nurse, or pharmacist, and might be provided verbally, in writing, or both. Also, as part of the usual approach, patients have regular visits with their doctor so that they can be watched for side effects and to answer any questions or concerns while taking their ET medication.

What are my choices if I decide not to take part in this study?

- You may choose to have the usual approach described above.
- You may choose to take part in a different research study, if one is available.

What will happen if I decide to take part in this study?

If you decide to take part in this study, you will either get text message reminders to take your medication or you will get a telephone-based counseling support program or both for up to 9 months, or you will get educational information about the ET medication that your doctor prescribed to you and about healthy living after breast cancer. If you get the counseling support program, the first two sessions can be done either by phone or video call.

After you finish participating in this support program, your doctor and study team will continue to follow your condition for up to 24 months after enrollment in the study to monitor your medication adherence.

What are the risks and benefits of taking part in this study?

There are both risks and benefits to taking part in this study. It is important for you to think carefully about these as you make your decision.

Risks

We want to make sure you know about a few key risks right now. We give you more information in the “What risks can I expect from taking part in this study?” section.

If you choose to take part in this study, there is a risk that the study approach may not be as good as the usual approach for helping you take your ET medication.

There may be some risks that the study doctors do not yet know about.

Benefits

There is some evidence that text message reminders and/or telephone-based counseling have helped people take their medication as prescribed for other medications and for other health conditions. It is not possible to know now if receiving these support programs during your ET medication treatment will help you to take your medication consistently, or helps lower the risk that breast cancer will come back compared to the usual approach. This study will help the study doctors learn things that may help people in the future.

If I decide to take part in this study, can I stop later?

Yes, you can decide to stop taking part in the study at any time.

If you decide to stop, let your study doctor know as soon as possible. If you stop, you can decide if you want to keep letting the study doctor know how you are doing.

Your study doctor will tell you about new information or changes in the study that may affect your health or your willingness to continue in the study.

Are there other reasons why I might stop being in the study?

Yes. The study doctor may take you off the study if:

- Your health changes and the study is no longer in your best interest.
- New information becomes available and the study is no longer in your best interest.
- You do not follow the study rules.
- The study is stopped by the National Cancer Institute (NCI), Institutional Review Board (IRB), or study sponsor (the Alliance). The study sponsor is the organization who oversees the study.

**It is important that you understand the information in the informed consent before making your decision.** Please read, or have someone read to you, the rest of this document. If there is anything you don’t understand, be sure to ask your study doctor or nurse.

What is the purpose of this study?

The purpose of this study is to test if different types of added support such as text message reminders and/or telephone based counseling can help increase your ability to take this medication regularly. The effects of these additional support programs will be compared to basic educational material provided by your study team about ET medication.

There will be about 1,180 people taking part in this study.

What are the study groups?

This study has 4 study groups.

- **Group 1**
  If you are in this group, you will receive the ET educational information provided to Group 4 described below. Also, you will receive daily text message reminders to take your ET medication and monthly text messages about how you are doing with taking your ET medication. You will receive these text messages for 9 months.

There will be about 290 people in this group.

- **Group 2**

If you are in this group, you will receive the ET educational content, provided to Group 4, as described below. Also, you will receive a total of five counseling sessions over the phone. These sessions are designed to support you while you take ET medication, develop your health goals, and stay on track in achieving those goals. Each session will last from 30-90 minutes. You may choose to use either phone or video for the first two counseling sessions. The sessions will take place for up to about 9 months.

There will be about 290 people in this group.

- **Group 3**

If you are in this group, you will receive the ET educational content, as described in Group 4 below. Also, you will receive daily text message reminders to take your medication and monthly text messages, as described for Group 1 above, as well as five counseling sessions, as described in Group 2 above. You will receive text messages for 9 months and counseling sessions for up to about 9 months.

There will be about 290 people in this group.

- **Group 4**

If you are in this group, you will get educational instructions about the ET medication you have been prescribed for your hormone receptor positive breast cancer. You will receive ET educational information on the study website at the start of your ET medication. Also, you will receive optional information about living a healthy life after breast cancer on a website.

There will be about 290 people in this group.

We will use a computer to assign you to one of the study groups. This process is called “randomization.” It means that your doctor will not choose and you cannot choose which study group you are in. You will be put into a group by chance. You will have an equal chance of being in each of the groups.

Another way to find out what will happen to you during this study is to read the chart below. Start reading at the left side and read across to the right, following the lines and arrows.

**Group 1**

Text Message Reminders & ET educational content

(Study Group)

**Group 2**

Phone counseling & ET educational content

(Study Group)

**Group 3**

Text message reminders, phone counseling & ET educational content

(Study Group)

You agree to take part in the study and sign this consent form.

Randomize –

The computer will randomly put you in a study group.

**Group 4**

ET educational content (Enhanced Usual Care)

What exams, tests, and procedures are involved in this study?

Before you begin the study your doctor will review the results of your exams, tests, and procedures. This helps your doctor decide if it is safe for you to take part in the study.

Listed below are exams, tests, and procedures that need to be done as part of this study but may not be included in your usual care. We will use them to carefully follow the effects of the provided support programs.

If you choose to take part in this study, you will be asked to complete surveys with questions about your personal health history, quality of life, and endocrine therapy adherence. Researchers will use this information to determine how the study activities (text message reminders and counseling program) affect endocrine therapy medication adherence.

**Electronic surveys:** For this study, you will be asked to install a survey application (or “app”) and answer questions on your personal smartphone. If you need help installing and/or using the survey app on your phone, ask for help from your study staff. The use of your own electronic device on a cellular network may result in a small cost to your data plan. Regardless of the device you use, your answers and personal information will not be stored on the device.

Your survey answers will be sent to the research database and will be kept private as described in the section below called, “Who will see my medical information?” Your e-mail address will only be used for this survey and will not be used for mail or marketing purposes. The Alliance will not keep your email address.

Since these forms are being used for research, the responses you provide will not be shared with your study doctor. If you have any serious health issues or health-related concerns, please talk with your doctor or nurse right away.

**Consent and Registration Visit:** At the time that you are considering participating in this study, you will be asked about when you started or plan to start ET medication. Study staff will ask you if your cellphone will work for the requirements of this study. Finally, you will be asked to provide your name, address, telephone number, telephone carrier (for example, Verizon, Sprint, T-Mobile, AT&T) and other personal information.

**Baseline Visit:** After you have been enrolled to the study, you will be asked to return to the clinic to complete the following procedures:

- You will download the survey app and shown how the app works;
- Complete the baseline electronic survey (about 40 minutes)
- You will then be told to which group you have been randomized
- You will be asked to watch a video, you will be shown the study participant web site, and be given other informational materials
- Lastly, you will be given a Pillsy^®^ Cap and pill bottle and will be shown how the Pillsy^®^ Cap works (see below)

**Survey Schedule:** You will be asked to complete more electronic surveys 5 more times throughout your participation in this study on the following schedule:

- 3 months after randomization (about 10 minutes),
- 6 months (about 10 minutes),
- 12 months (about 45 minutes),
- 18 months (about 10 minutes), and
- 24 months (about 30 minutes) after randomization.

The surveys will ask about things like your symptoms, mood, stress, fears, social support, socio-demographics and medication adherence. You don’t have to answer any question that makes you feel uncomfortable.

**Pillsy^®^ Cap:** You will also be asked to use a Pillsy^®^ Cap throughout your participation in this study. You will be asked to install the Pillsy^®^ app on your personal smartphone. The Pillsy^®^ Cap is a medication pill bottle cap that will keep track of when you take your ET medication.

In addition to the Pillsy® Cap, you will also be given a study-provided pill bottle. You may choose to use the study-provided pill bottle along with the Pillsy® Cap, or you may choose to move the Pillsy® Cap to your pharmacy-provided pill bottle. Each time you refill your prescription you will need to place your pills into the study-provided pill bottle, or you will need to move the Pillsy® Cap to the pill bottle you receive from your pharmacy.

What risks can I expect from taking part in this study?

General Risks

If you choose to take part in this study, there is a risk that the study approach may not be as good as the usual approach for helping you take your ET medication.

You also may have the following discomforts:

- Spend more time in the hospital or doctor’s office.
- Be asked sensitive or private questions about things you normally do not discuss.
- It is also possible that you may have some anxiety from discussing and answering questions about your ET medication. We recognize that this is a sensitive subject and some people would prefer not to talk about these issues. If you feel uncomfortable or nervous while completing the surveys, you may skip any questions on the surveys that you feel uncomfortable answering.
- There is a small risk that your study information could become known to someone who is not involved in performing or monitoring this study. However, we make every effort to protect your privacy.

What are my responsibilities in this study?

If you choose to take part in this study you will need to:

- Keep your in-person study appointments with research staff at your site; also, depending on your group assignment, keep your telephone counseling appointments.
- Respond to study text messages and phone calls
- Tell your doctor about:
  - all medications and supplements you are taking
  - any side effects
  - any doctors’ visits or hospital stays outside of this study
  - if you have been or are currently in another research study.

What are the costs of taking part in this study?

You and/or your insurance plan will need to pay for the costs of medical care you get as part of the study, just as you would if you were getting the usual care for your hormone receptor positive breast cancer.

Taking part in this study may mean that you need to make more visits to the clinic or hospital than if you were getting the usual approach to treat your cancer. You may:

- Have more travel costs.
- Need to take more time off work.
- Have other additional personal costs.

Also, due to the nature of the required study activities, you may have extra data or phone charges, if you do not have unlimited phone/data plan with your cellular network provider. Use of the ePRO survey app and Pillsy app uses data on a cellular network data plan. However, these apps can also be used on a Wi-Fi network. Free Wi-Fi networks can be found at public places such as coffee shops, libraries, etc.

You will receive a gift card worth $50 for completion of your baseline visit. You will then receive an additional gift card worth $20 for completion of each of the 3, 6, 12, 18, and 24 month surveys. You will receive a gift card worth $55 for completion of the final Pillsy^®^ Cap data collection. The most that you could receive for participation in this study is $205.

In addition, you may keep your Pillsy^®^ cap at the end of the study. More information on the Pillsy^®^ cap will be provided to you separately.

What happens if I am injured because I took part in this study?

If you are injured as a result of taking part in this study and need medical treatment, please talk with your study doctor right away about your treatment options. The study sponsors will not pay for medical treatment for injury. Your insurance company may not be willing to pay for a study-related injury. Ask them if they will pay. If you do not have insurance, then you would need to pay for these medical costs.

If you feel this injury was caused by medical error on the part of the study doctors or others involved in the study, you have the legal right to seek payment, even though you are in a study. Agreeing to take part in this study does not mean you give up these rights.

Who will see my medical information?

Your privacy is very important to us. The study doctors will make every effort to protect it. The study doctors have a privacy permit to help protect your records if there is a court case. However, some of your medical information may be given out if required by law. If this should happen, the study doctors will do their best to make sure that any information that goes out to others will not identify who you are.

Some of your health information, such as your response to cancer treatment, results of study tests, and medicines you took, will be kept by the study sponsor in a central research database. If information from this study is published or presented at scientific meetings, your name and other personal information will not be used.

There are researchers for this study located at the University of North Carolina at Chapel Hill (UNC) and the Ohio State University (OSU). These researchers are responsible for providing the text message reminders and telephone counseling sessions. In order for these researchers to be able to contact you for study activities, it will be necessary for you to provide your name, mailing address, mobile phone number, mobile phone carrier, and an alternate phone number. You will provide this information on the “Patient Mailing Contact Information” form that will be securely faxed to the study staff at OSU. The “Patient Mailing and Contact Information Form” will be destroyed upon completion of the study. Your contact information will be kept only for the duration of the study. The Pillsy^®^ Cap app will store your initials and your study patient ID number.

If you receive text messages as part of your study group, no sensitive health information will be included in texts from the study team to you and you will only be addressed by your first name when receiving texts, regardless of your cell phone carrier. For more specific information regarding privacy for your cell phone carrier, please review the End User License Agreement with your cell phone service provider.

If you are receiving a study-provided cell phone, your name and other sensitive information will not be shared with Verizon Wireless. Instead of names, subject identification numbers will be used in documents regarding the Verizon phone program to keep your identity confidential.

There are organizations that may look at or receive copies of some of the information in your study records. Your health information in the research database also may be shared with these organizations. They must keep your information private, unless required by law to give it to another group.

Some of these organizations are:

- The study sponsor (Alliance)
- The Ohio State University and the University of North Carolina, where your contact and telephone information will be stored.
- The NCI Central IRB, which is a group of people who review the research with the goal of protecting the people who take part in the study.
- The NCI and the groups it works with to review research.
- The NCI’s National Clinical Trials Network and the groups it works with to conduct research

In addition to storing data in the study database, data from studies that are publicly funded may also be shared broadly for future research with protections for your privacy. The goal of this data sharing is to make more research possible that may improve people’s health. Your study records may be stored and shared for future use in public databases. However, your name and other personal information will not be used.

Some types of future research may include looking at your information and information from other patients to see who had side effects across many studies or comparing new study data with older study data. However, right now we don’t know what research may be done in the future using your information. This means that:

- You will not be asked if you agree to take part in the specific future research studies using your health information.
- You and your study doctor will not be told when or what type of research will be done.
- You will not get reports or other information about any research that is done using your information.

As part of this study, we will collect information from an application downloaded from the Internet called “Patient Cloud.” You may choose to download the app from the Internet through the App store on your own device (iPhone® or iPad®) or an Android device, using your Apple ID or Google account.

The maker of the application and/or device may collect and store personal information, such as health information, location data, and internet usage. A complete description of what data will be collected and what the company will do with it can be found in the Terms of Service. You will need to agree to the Terms of Service to participate in this study.

The researchers in this study may not have any control over what the company does with your information. The application and/or device may collect and transmit more information to the company than is needed for this study.

Where can I get more information?

You may visit the NCI web site at http://cancer.gov/ for more information about studies or general information about cancer. You may also call the NCI Cancer Information Service to get the same information at: 1-800-4-CANCER (1-800-422-6237).

A description of this clinical trial will be available on http://www.ClinicalTrials.gov, as required by U.S. Law. This Web site will not include information that can identify you. At most, the Web site will include a summary of the results. You can search this Web site at any time.

You can talk to the study doctor about any questions or concerns you have about this study or to report side effects or injuries. Contact the study doctor (*insert name of study doctor[s]*) at (*insert telephone number, and email address if appropriate*).

For questions about your rights while in this study, call the (*insert name of organization or center*) Institutional Review Board at (*insert telephone number*).

My signature agreeing to take part in the study

I have read this consent form or had it read to me. I have discussed it with the study doctor and my questions have been answered. I will be given a signed and dated copy of this form. I agree to take part in the main study.

Participant’s signature

Date of signature

Signature of person(s) conducting the informed consent discussion

Date of signature
