# Supplementary material for: Randomized phase III trial evaluating motivational interviewing and text interventions to optimize adherence to breast cancer endocrine therapy (Alliance A191901): the GETSET protocol
Source: Trials. 2023 Oct 12;24:664. doi: 10.1186/s13063-023-07672-8 (PMC10568920; doi:10.1186/s13063-023-07672-8)
Supplement: Supplementary file 3 — Additional file 3. Optimizing endocrine therapy through motivational interviewing and text interventions. [file 13063_2023_7672_MOESM3_ESM.pdf]

ALLIANCE FOR CLINICAL TRIALS IN ONCOLOGY

ALLIANCE A191901

OPTIMIZING ENDOCRINE THERAPY THROUGH MOTIVATIONAL INTERVIEWING AND TEXT INTERVENTIONS

ClinicalTrials.gov Identifier: NCT04379570

Study Co-Chair

Katherine E. Reeder-Hayes, MD
University of North Carolina – Chapel Hill
Lineberger Comprehensive Cancer Center
101 East Weaver Street
Chapel Hill, NC 27599
Tel: 919-445-6147
kreeder@med.unc.edu

Study Co-chair
Stephanie B. Wheeler, PhD MPH
Univ. of North Carolina – Chapel Hill
Tel: 919-966-7374
stephanie_wheeler@unc.edu

Community Oncology Co-chair
Judith Owen Hopkins, MD
Novant Health Forsyth Medical Center
Tel: 336-564-4170
johopkins@novanthealth.org

Health Outcomes Co-chair
Michelle J. Naughton, PhD, MPH
Ohio State University Comprehensive Cancer Center
Tel: 614-293-6390
Michelle.Naughton@osumc.edu

Health Disparities Co-chair
Electra D. Paskett, PhD
Ohio State University
Tel: 614-293-7712
Electra.paskett@osumc.edu

Health Disparities Committee Chair
Lucille Adams-Campbell, PhD
Georgetown University
Tel: 202-697-3770
lla9@georgetown.edu

Breast Committee Co-Chairs

Ann Partridge, MD, MPH
Dana Farber Cancer Center
Tel: 617- 632-3800
ahpartridge@partners.org

Lisa Carey, MD
Univ. of North Carolina
Tel: 919- 966-4431
Lisa_carey@med.unc.edu

Primary Statistician
Heather Gunn, PhD
Tel: 507-422-6526
Gunn.Heather2@mayo.edu

Secondary Statistician
Paul Novotny, MS
Tel: 507-284-4186
novotny@mayo.edu

Data Manager
Isabelle Gadeke
Tel: 507-422-6252
Sorensen.Isabelle@mayo.edu

Protocol Coordinator
Rachel Wills
Tel: 773-702-9814
rwills@bsd.uchicago.edu

Participating NCTN Groups:
Alliance/Alliance for Clinical Trials in Oncology (lead), ECOG-ACRIN / ECOG-ACRIN Cancer Research Group, NRG / NRG Oncology, SWOG / SWOG

Study Resources:

Expedited Adverse Event Reporting<br>https://ctepcore.nci.nih.gov/ctepaers	Medidata Rave® iMedidata portal<br>https://login.imedidata.com
OPEN (Oncology Patient Enrollment Network)<br>https://open.ctsu.org	

Protocol Contacts:

A191901 Nursing Contact
Barbara Kleiber MACPR, BSN, RN, OCN, CCRC
The Ohio State University
Tel: 614-293-1815
barbara.kleiber@osumc.edu

A191901 Pharmacy Contact
Heidi D. Finnes, PharmD, BCOP
Mayo Clinic
Tel: 507-538-7066
finnes.heidi@mayo.edu

A191901 Central Study Project Manager
Project Manager
UNC Chapel Hill
Tel: 919-445-6147
Fax: 919-966-4330
GETSETstudy@unc.edu

Protocol-related questions may be directed as follows:

**Questions**	**Contact (via email)**
Questions regarding patient eligibility, treatment, and dose modification:	Study Chair, Nursing Contact, Protocol Coordinator, and (where applicable) Data Manager
Questions related to data submission, RAVE or patient follow-up:	Data Manager
Questions regarding the protocol document and model informed consent:	Protocol Coordinator
Questions related to IRB review	Alliance Regulatory Inbox<br>regulatory@allianceNCTN.org
Questions regarding CTEP-AERS reporting:	Alliance Pharmacovigilance Inbox<br>pharmacovigilance@alliancenctn.org

CANCER TRIALS SUPPORT UNIT (CTSU) ADDRESS AND CONTACT INFORMATION

**For regulatory requirements:**	**For patient enrollments:**	**For data submission:**
Regulatory documentation must be submitted to the Cancer Trials Support Unit (CTSU) via the Regulatory Submission Portal.<br>(Sign in at https://www.ctsu.org, and select the Regulatory > Regulatory Submission.)<br><br>Institutions with patients waiting that are unable to use the Portal should alert the CTSU Regulatory Office immediately by phone or email: 1-866-651-CTSU (2878), or CTSURegHelp@coccg.org to receive further instruction and support.<br><br>Contact the CTSU Regulatory Help Desk at 1-866-651-CTSU (2878) for regulatory assistance.	Refer to the patient enrollment section of the protocol for instructions on using the Oncology Patient Enrollment Network (OPEN). OPEN is accessed at https://www.ctsu.org/OPEN_SYSTEM/ or https://OPEN.ctsu.org.<br><br>Contact the CTSU Help Desk with any OPEN related questions by phone or email : 1-888-823-5923, or ctsucontact@westat.com.	Data collection for this study will be done exclusively through Medidata Rave. Refer to the data submission section of the protocol for further instructions.

The most current version of the **study protocol and all supporting documents** must be downloaded from the protocol-specific page located on the CTSU members' website (https://www.ctsu.org). Access to the CTSU members' website is managed through the Cancer Therapy and Evaluation Program - Identity and Access Management (CTEP-IAM) registration system and requires log in with a CTEP-IAM username and password.

Permission to view and download this protocol and its supporting documents is restricted and is based on person and site roster assignment housed in the CTSU Regulatory Support System (RSS).

For clinical questions (i.e. patient eligibility or treatment-related) see the Protocol Contacts, Page 2.

For non-clinical questions (i.e. unrelated to patient eligibility, treatment, or clinical data submission) Contact the CTSU Help Desk by phone or email:
CTSU General Information Line – 1-888-823-5923, or ctsucontact@westat.com. All calls and correspondence will be triaged to the appropriate CTSU representative.

OPTIMIZING ENDOCRINE THERAPY THROUGH MOTIVATIONAL INTERVIEWING AND TEXT INTERVENTIONS

Eligibility Criteria (see Section 3.0)

- Women with an initial pathologically confirmed initial diagnosis of stage I-III, hormone receptor positive, HER2-neu negative invasive breast cancer within 18 months prior to registration (See §3.2.1).
- Patients must have completed any planned cancer-directed surgery (except reconstruction or oophorectomy) and completed any planned adjuvant therapy prior to registration (See §3.2.2).
- Patients must be taking an endocrine therapy drug initiated no earlier than 6 months prior to registration OR have received a prescription with stated intent to initiate within 6 weeks after registration (See §3.2.3).
- Patients must have no history of cancer as follows: no prior history of invasive or non-invasive breast cancer at any time, and no history of non-breast cancer within the last 5 years, excluding non-melanoma skin cancer (See §3.2.4).
- Patients must be willing to use a smart phone for study activities (See §3.2.5).
- Patients must be willing to use a Pillsy medication event monitoring system (“Pillsy cap”) for study activities (See §3.2.6).
- Age ≥ 18 years
- Patients must be able to speak and read English

Required Initial Laboratory Values

None

Schema

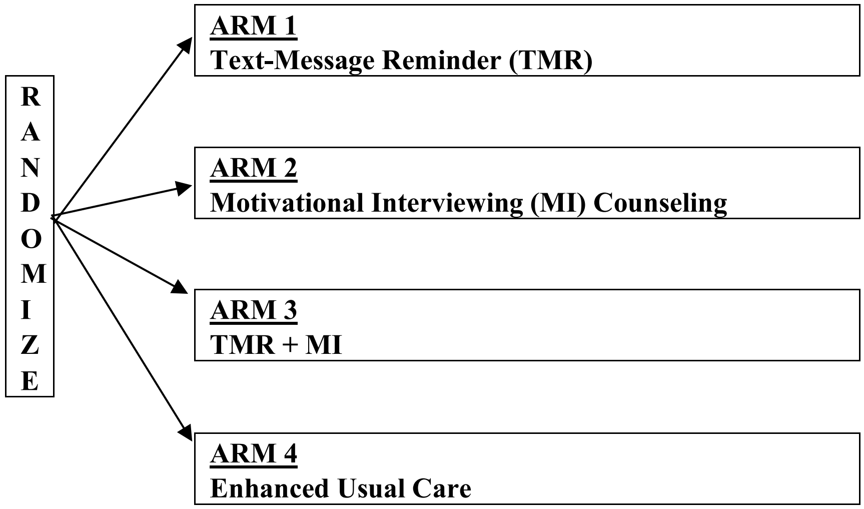

Please refer to the full protocol text for a complete description of the eligibility criteria and intervention plan.

Table of Contents

1.0 Background 8
1.1 Introduction 8
1.2 Conceptual and Theoretical Model of ET Medication Taking Behaviors 9
1.3 Gaps Addressed in this Study 10
1.4 Study Overview 11
1.5 Preliminary Data & Study Design Considerations 12
1.6 Study Design 15
2.0 Objectives 15
2.1 Primary objective 15
2.2 Secondary objective 16
2.3 Exploratory objectives 16
3.0 Patient Selection 16
3.1 On-Study Guidelines 16
3.2 Eligibility Criteria 16
4.0 Patient Registration 18
4.1 Investigator and Research Associate Registration with CTEP 18
4.2 CTSU Site Registration Procedures 19
4.3 Patient Registration Requirements 21
4.4 Patient registration/randomization procedures 22
4.5 Stratification Factors and Treatment Assignments 23
5.0 Study Calendar 24
6.0 Data and Specimen Submission 25
6.1 Data Collection and Submission 25
6.2 Submission of Patient Completed Measures 26
7.0 Intervention 27
7.1 Screening & Informed Consent Visit 27
7.2 Initiation & Baseline Visit 29
7.3 Text-Message Reminder (TMR) Component (Arms 1 and 3) 31
7.4 Motivational Interviewing (MI) Counseling Component (Arms 2 and 3) 32
7.5 Enhanced Usual Care (Arm 4) 37
7.6 Incentives 38
8.0 Adverse Events 38
9.0 Measures 38
9.1 Alliance Patient Questionnaire – Socio-demographics (Appendix II) 39

9.2 Personal Health History (Appendix III) 39
9.3 PROMIS Global Health Questionnaire v1.2 (Appendix IV) 39
9.4 Breast Cancer Prevention Trial (BCPT) Symptom Questionnaire (Appendix V) 39
9.5 PROMIS Depression Short Form 8a v1.0 (Appendix VI) 40
9.6 Concerns About Recurrence Scale (CARS) (Appendix VII) 40
9.7 Perceived Stress Scale (PSS) (Appendix VIII) 40
9.8 Modified Medical Outcomes Study Social Support (mMOS-SS) Questionnaire (Appendix IX) 40
9.9 Medical Use Self-Efficacy Scale (MUSE) (Appendix X) 40
9.10 Self-reported Endocrine Therapy Adherence and Persistence (Appendix XI) 40
9.11 Intervention Evaluation: Acceptability, Patient Satisfaction, and Appropriateness (Appendix XII) 41
9.12 Was It Worth It (WIWI) Questionnaire for Alliance Clinical Trial Participation (Appendix XIII) 41
10.0 End of Treatment/Intervention 41
10.1 Duration of Protocol Intervention 41
10.2 Criteria for Discontinuation of Protocol Intervention 42
10.3 Follow-up 42
10.4 Extraordinary Medical Circumstances 45
10.5 Managing ineligible patients and registered patients who never receive protocol intervention 45
10.6 Follow-up for patients who are registered, but who never start study intervention 45
11.0 Statistical Considerations 45
11.1 Study Design 45
11.2 Description of Randomization Routine 46
11.3 Sample Size, Accrual Time, and Study Duration 46
11.4 Statement for Primary Endpoint 47
11.5 Statement for Secondary Endpoints 48
11.6 Statement for Exploratory Endpoints 49
11.7 Missing Data 52
11.8 Study Monitoring 52
11.9 Inclusion of Women and Minorities 53
12.0 Credentialing Requirements 55
12.1 Institutional Credentialing 55
13.0 References 56
Appendix I: Patient Mailing and Contact Information Form 60
Appendix II: Alliance Patient Questionnaire – Socio-demographics 62
Appendix III: Personal Health History 65

Appendix IV: PROMIS Global Health Questionnaire v1.2 67
Appendix V: Breast Cancer Prevention Trial (BCPT) Symptom Questionnaire 69
Appendix VI: PROMIS Depression Short Form 8a v1.0 71
Appendix VII: Concerns About Recurrence Scale (CARS) 72
Appendix VIII: Perceived Stress Scale (PSS) 74
Appendix IX: Modified Medical Outcomes Study Social Support Survey (mMOS-SS) 75
Appendix X: Medical Use Self-Efficacy Scale (MUSE) 76
Appendix XI: Self-reported Endocrine Therapy Adherence and Persistence 78
Appendix XII: Intervention Evaluation: acceptability, Patient Satisfaction, and Appropriateness 81
Appendix XIII: Was It Worth It (WIWI) Questionnaire 82
Appendix XIV: Text Message Reminder (TMR) Messages and Interactive Monthly Questions 83
Appendix XV: Alliance ePRO 87
Appendix XVI: Semi-Structured Qualitative Interview Guide For Process Evaluation 89
Appendix XVII: Text Message Reminder (TMR) Intervention Evaluation 91
Appendix XVIII: iPhone Recipient Evaluation Survey 92
Appendix XIX: iPhone Participant Responsibilities and Agreement Form 94
Appendix XX: A191901 Recruitment Scripts 96
Appendix XXI: Telephone Consent Procedure & Script 100

1.0 BACKGROUND

1.1 Introduction

Hormone Receptor-positive (HR+) breast cancer is an important public health problem, with approximately 184,000 new cases diagnosed among US women annually [1]. Survival rates of HR+ breast cancer have improved steadily over the past three decades, due in part to the introduction of adjuvant endocrine therapy (ET) in the early 1990s. Taken as a once-daily oral medication, ET has proven to be a dramatically efficacious targeted therapy, with reductions in 10 year recurrence of 47% following five years of tamoxifen [2], and further incremental benefits from the newer aromatase inhibitor drugs and longer treatment courses of up to 10 years [3, 4]. Today, oral ET for five to ten years is the standard of care for HR+ breast cancer.

Despite its therapeutic success, non-adherence to endocrine therapy is a critical barrier to maximally effective treatment in HR+ breast cancer. Several recent studies have shown that patients struggle with long term ET adherence. (Within this application, “non-adherence” will refer to both early discontinuation and sub-optimal dosing of ET medications, generally considered to be below a threshold of 80% of prescribed doses taken)[5]. Hershman and colleagues reported that only 49% of patients in a large commercial insurance cohort took ET for the full recommended duration at the optimal schedule [6]. In Medicaid and Medicare populations, adherence difficulties emerge even earlier with an estimated 36% non-adherent in the first year [7] and 50% non-adherent by year four [8]. ET non-adherence has been linked to significant decrements in cancer-specific and overall survival in observational studies [9, 10], and clinical trials comparing different durations of tamoxifen have shown higher recurrence rates for shorter durations of therapy [11].

Many of the factors associated with ET non-adherence are potentially modifiable. Factors affecting adherence can be broadly divided into four categories: patient experiences with ET (e.g., side effects and poor communication with providers about side effects), attitudes and beliefs (e.g., regarding recurrence risk and perception of ET benefits), forgetfulness, and structural barriers (e.g., cost). Patient experience factors associated with ET non-adherence include a higher side effect burden [12, 13], lower health-related quality of life at baseline and while taking ET [14-16], lower self-efficacy [17], and poor satisfaction with decision-making and with treatment [13, 18], all of which appear to be more prevalent issues among black women and younger women (<50 yrs) [13, 19, 20]. Attitudes and beliefs associated with non-adherence include a woman’s belief that her risk of recurrence is low and that the risk will not change if ET is discontinued [13, 20]. Our team previously found that these beliefs are more common among black breast cancer survivors, and that adjusting for such beliefs attenuates the effect of side-effect burden on non-adherence [13]. In addition, many patients report that they forget to take ET in various situations [13, 18]. Structural factors associated with non-adherence include higher co-payment [21], while longer refill intervals and Medicare part D low-income subsidies may protect against non-adherence [22].

Although modifiable factors affecting ET adherence are well-documented, few effective interventions have been reported to date to address the widespread problem of ET non-adherence in breast cancer [23]. Development of such interventions faces multiple challenges. Effective designs must address barriers across a wide array of unrelated domains, from forgetfulness and perceptions of risk/benefit tradeoffs to side effect concerns and self-efficacy [6, 19, 21, 24-26]. Due to the large number of patients potentially affected, and the competing demands of busy oncology clinics, feasible designs must find ways to reach patients outside the clinical encounter, and to connect with high-risk but difficult-to-recruit populations, such as younger breast cancer survivors and minority patients. Finally, if health system stakeholders are to adopt ET adherence interventions on a large scale, data regarding the resource use and benefits of the intervention must be provided.

Successful strategies exist to improve medication adherence in non-cancer populations, but these strategies have yet to be successfully applied to endocrine therapy delivery. In cardiovascular disease, multi-faceted interventions that address a variety of barriers appear to be most effective [27]. Motivational interviewing (MI) improves adherence in multiple studies of HIV and other chronic

diseases [28-31]. MI is a flexible counseling approach that identifies patient-specific barriers, clarifies goals of care, and assists patients in developing their own solutions [29, 32]. Because of its patient-centered design, MI is inherently culturally competent and potentially responsive to a wide range of barriers that diverse women may face. Alternatively, interventions leveraging mobile technology through "smart" devices may offer opportunities to reach more patients in an efficient manner; specifically, text message reminder (TMR) systems have been linked to improved adherence in cardiovascular disease patients [33, 34]. However, text messaging may not be able address the same range of barriers as more intensive interventions, and the optimal combination of approaches remains unknown.

1.2 Conceptual and Theoretical Model of ET Medication Taking Behaviors

We adapted the integrative, poly-theoretical, conceptual framework used by Golin and colleagues for oral medication adherence research [32, 35] to identify factors that likely influence ET adherence among breast cancer survivors and inform our intervention design and variable selection (Figure 1). This framework, postulates that if people have strong motivation, necessary self-efficacy to follow through, and minimal social, environmental or structural barriers, they are likely to take medications as prescribed [32, 35-37]. The many factors that influence a person's motivation and self-efficacy to take ET are grouped into four broad categories: person-level; provider-level; regimen-based; and social/structural-level. In this model, motivation and self-efficacy directly influence medication adherence [6, 19, 20] and are affected, not only by regimen characteristics (e.g., dose frequency and side effects), but also by person-level features such as health beliefs, health status, attitudes, and risk perceptions [12-14, 16]. Moreover, both motivation and self-efficacy are influenced by external factors known to affect medication taking, including provider and social/structural-level factors [26, 35, 38, 39]. To address this multitude of factors influencing medication adherence, appropriate interventions must: (1) identify factors that inhibit and enhance motivation and self-efficacy; (2) raise patients' awareness of their relative level of motivation and self-efficacy; and (3) help patients develop strategies to overcome barriers and enhance facilitators [40]. By identifying individual precursors to motivation and self-efficacy, our intervention can promote readiness to change across diverse populations.

Figure 1. Conceptual framework for improving medication taking behavior using motivational interviewing, adapted from Adamian et al., 2004[34]

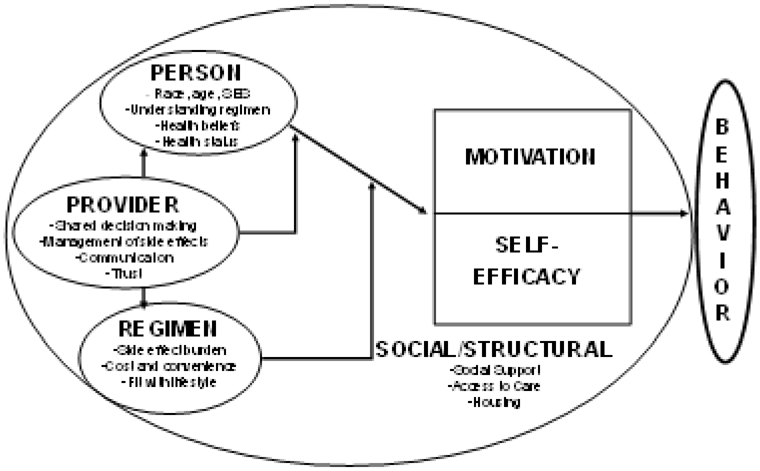

1.2.1 Theoretical Basis of Motivational Interviewing

Motivational Interviewing counseling is a research-tested behavioral intervention that targets known barriers to healthy behaviors, yet also allows a highly individualized application of the model [31, 32, 41]. MI is appropriate in our context because it ascertains how ready patients are to adhere to medical recommendations in terms of both motivation and self-efficacy, helps identify adherence barriers, and develop solutions to address numerous types of barriers. Best practices for MI indicate that greatest success is achieved after multiple (usually 4-5) sessions with a trained MI counselor. After multiple sessions, patients develop the skills and capabilities needed to clarify their goals and problem-solve solutions on their own. In conjunction with

counseling sessions, we provide a workbook incorporating characters who are diverse in both race and age, an important component of similar interventions [30]. This workbook is structured to follow MI session content, introduce concepts that may require deeper exploration, allow journaling outside of the MI sessions, and record goals and decisions.

1.2.2 Theoretical Basis of Text Messaging Reminders

Mobile text messaging is a straightforward way to reach patients without the need for face-to-face contact, and addresses several factors in our conceptual model, including providing cues to action, overcoming forgetfulness, boosting motivation, communicating positive messages that affect health beliefs, promoting self-efficacy and communication with medical providers. TMR also offers the benefits of message automation for efficient outreach at the population scale. The set of daily messages used in the TMR pilot study, which will be further adapted and expanded for the proposed study, were pre-tested for acceptability and appropriateness among diverse breast cancer patients. The TMR pilot study also included regular queries of self-reported adherence (e.g., *How many ET pills did you take in the past 7 days?*), which will occur monthly in this study. These monthly questions will not be a part of the study data collection. When women responded that they had taken fewer than 6 pills in any 7-day period, a response message probed on barriers, with barrier-specific response options (e.g., for serious problems with side effects, a text response might encourage a woman to reach out to her doctor for assistance). These interactive TMR components can be fully automated, with routine monitoring by the Ohio State University to ensure that patients' responses are reasonable and do not warrant a more direct intervention. TMR allows patients to indicate and receive guidance for overcoming common barriers, and directly addresses forgetfulness through daily reminders.

1.2.3 Rationale for Text Messaging Reminders + Motivational Interviewing Combination Intervention

In other medication settings, multi-component interventions have had greater efficacy in improving adherence. A multicomponent intervention consisting of both MI and TMR may better target complex barriers and reinforce motivation and self-efficacy through complementary formats. However, this potential gain in efficacy likely comes at the expense of additional resources, more complexity in delivery, and potentially lower feasibility for broad dissemination. This randomized controlled trial (RCT) will therefore explore descriptively the marginal gains of TMR+MI over either component alone, as well as the cost trade-offs involved.

1.3 Gaps Addressed in this Study

The effectiveness of endocrine therapy is threatened by medication non-adherence, which is common and associated with increased mortality [6, 42]. Intervention is particularly needed for young women and black women, who are at high risk for non-adherence [13, 43]. TMR and MI approaches have been successful in promoting medication adherence in other disease populations, but to our knowledge, have not been tested in breast cancer patients within a RCT setting—although there have been recent studies of short duration in small samples using smart phones for monitoring ET adherence [44] and assessing acceptability of smart phone-based adherence tools for ET [45]. Our study will comprehensively evaluate the impact of smart phone-based text messaging reminders and telephone-delivered motivational interviewing on ET adherence, with a prospective randomized design and sampling framework that permit full evaluation of effects in young and minority populations, as well as resource use and value. Our proposed TMR and MI counseling interventions have been rigorously pilot-tested and address multiple barriers to adherence, including cue-to-action, accountability, information, and motivation (e.g., perceived benefits and harms tradeoffs), and self-efficacy. This trial brings TMR and MI interventions into the cancer medication adherence space for the first time in a RCT of a large number of patients. The scientific premise of our study is that medication adherence interventions using motivational interviewing and text messaging reminders

can be successfully translated to the breast cancer space and used to increase endocrine therapy adherence, thereby impacting HR+ breast cancer recurrence and mortality.

1.4 Study Overview

We propose to conduct a 4-arm RCT to test the efficacy, effect on patient-reported outcomes, and resource use requirements, of two interventions to improve ET adherence among HR+ breast cancer survivors: (1) a text messaging reminder (TMR) system and (2) a telephone-based motivational interviewing (MI) counseling. We will also explore whether the combination of TMR+MI may be more efficacious. This RCT will be tested through the Alliance for Clinical trials in Oncology (Alliance), a national network supported by the National Cancer Institute (NCI) comprising 10,000 cancer specialists at hospitals, medical centers, and community clinics. We will over-sample groups at high risk of non-adherence, including black patients and patients under age 50. The intervention materials and design have been pre-tested to be culturally appropriate and salient to specific barriers faced by racially- and age-diverse patients. In this study, an efficient study design will be used to compare each intervention and their combination to usual care, in terms of: Pillsy-reported ET adherence (Aim 1), patient-reported outcomes including medication self-efficacy, health-related quality of life, cancer worry, knowledge and attitudes about ET (Aim 2), and relative resource use (cost) and value of interventions (cost-effectiveness) (Aim 3). We will also evaluate whether the intervention effects on outcomes differ by race and age. An overview of study arms, activities, and outcomes assessment is presented in Figure 2 below.

Figure 2. Study schema depicting timing of intervention activities and outcome assessment

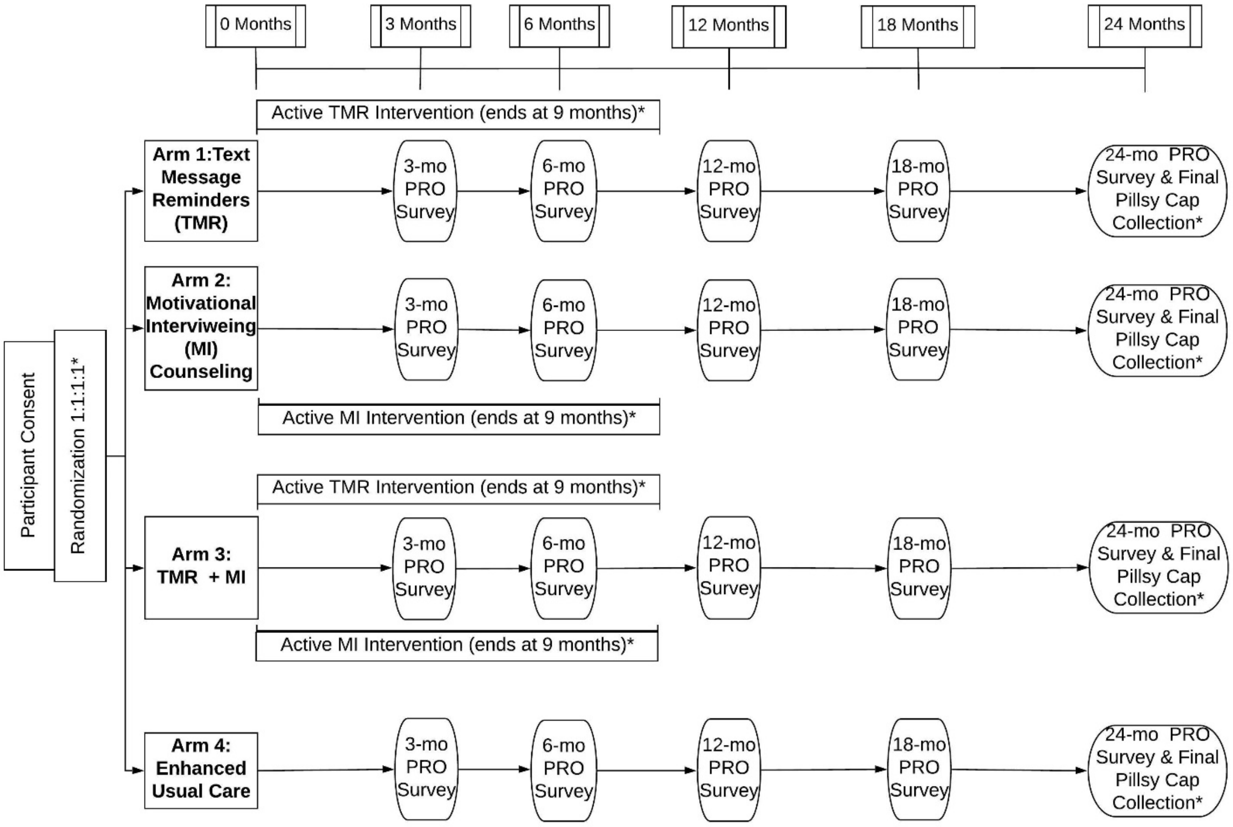

*NOTES:
(1) randomization will be stratified by race and age to ensure balance between arms
(2) If participant has not initiated endocrine therapy yet at consent, study activities and tracking will be delayed until initation is confirmed
(3) Pillsy cap data will be collected in real time through participant smartphone, and if needed, will be collected at clinic visits

1.5 Preliminary Data & Study Design Considerations

1.5.1 Evidence for Oversampling of Young Women and Black Women

Lower ET adherence within these groups across multiple studies, including our own, suggests that the drivers of ineffective ET use vary across diverse populations [10, 24, 43, 46]. We have pursued a series of related studies to understand ET adherence within key populations of interest, including black, young, and low-income women with breast cancer. First, we analyzed linked Medicaid administrative billing and cancer registry data to assess ET initiation among hormone receptor positive (HR+) breast cancer patients diagnosed in North Carolina; less than 50% had a pharmacy claim for ET within 15 months of diagnosis, suggesting significant failure to initiate in this population [47]. Second, we recruited more than 50 HR+ breast cancer patients and 20 oncology care providers for interviews to understand key barriers and facilitators to ET use; we learned that patients and providers expressed very different views of the ET experience and that non-adherers struggled with patient-provider communication, risk-benefit tradeoffs, and side effects that influenced their adherence behaviors [48]. Third, we analyzed multi-payer insurance data in North Carolina to explore patterns of ET initiation and adherence by race; we found that younger black women, particularly those with higher-risk disease were less likely to initiate endocrine therapy [43], and less likely to be adherent in the first year following initiation (unpublished data). Fourth, we developed a multi-item questionnaire on ET adherence, ET-related symptoms, and quality of life, which was administered to 1,280 HR+ patients and found significant differences in overall adherence 2-years post-diagnosis, by race (16% of white women non-adherent versus 24% of black women; $p<0.05$), with significant differences by race in the reasons for ET non-adherence (Figure 3).

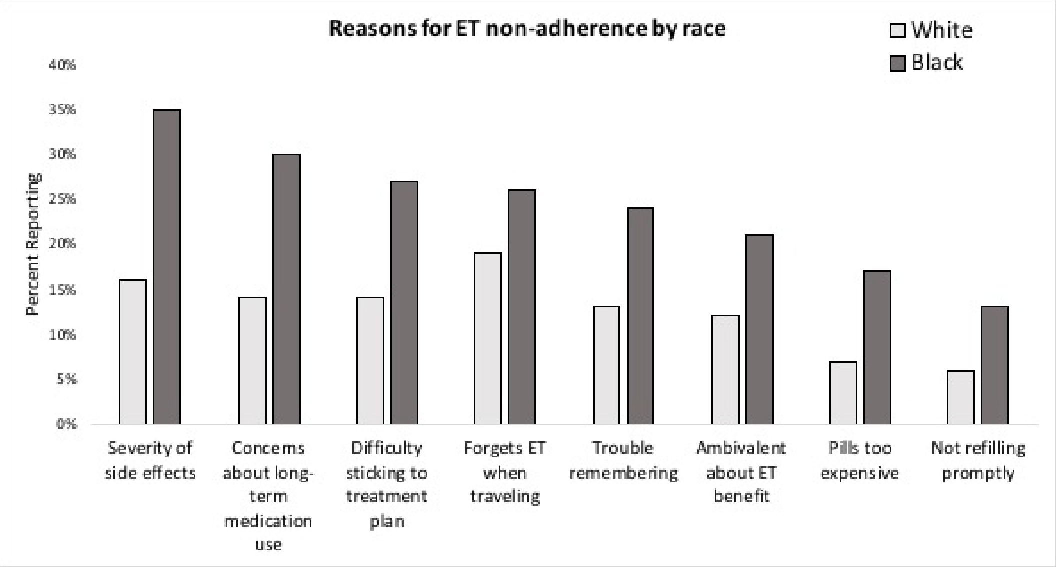

1.5.2 Use of Motivational Interviewing to Support ET Adherence

Motivational Interviewing (MI) is a promising behavioral intervention to support ET use. As part of an ongoing grant from the American Cancer Society, Drs. Wheeler and Reeder-Hayes developed and pilot-tested a single-arm MI-based intervention to promote ET adherence over a 1-year period. We used diverse patient focus groups to pre-test key content, graphic design and formatting, and acceptability of our intervention materials. We also met with oncologists and clinical staff to gauge clinical feasibility, acceptability, and appropriateness of the intervention. We recruited 42 participants at five oncology clinics within the UNC Cancer Care network to the GET SET (Guiding Endocrine Therapy Success through Empowerment and Teamwork) intervention study. We oversampled black and young women, recruiting a total of 15 black and 27 non-black patients; median age of participants was 55 years (range 25-73). All

participants were offered an educational video, 5 MI sessions (1 in-person, 4 via telephone), MI workbook, and evidence-based resource guide.

Preliminary results of this pilot study are summarized in Figure 4 and indicate promising ET adherence among black women in particular at 12 months using proportion of days covered (PDC) with ET medication as measured with Medication Event Monitoring systems caps and using an 80% threshold to evaluate adherence (dashed line).

Figure 4: GETSET ET Adherence at 12 months

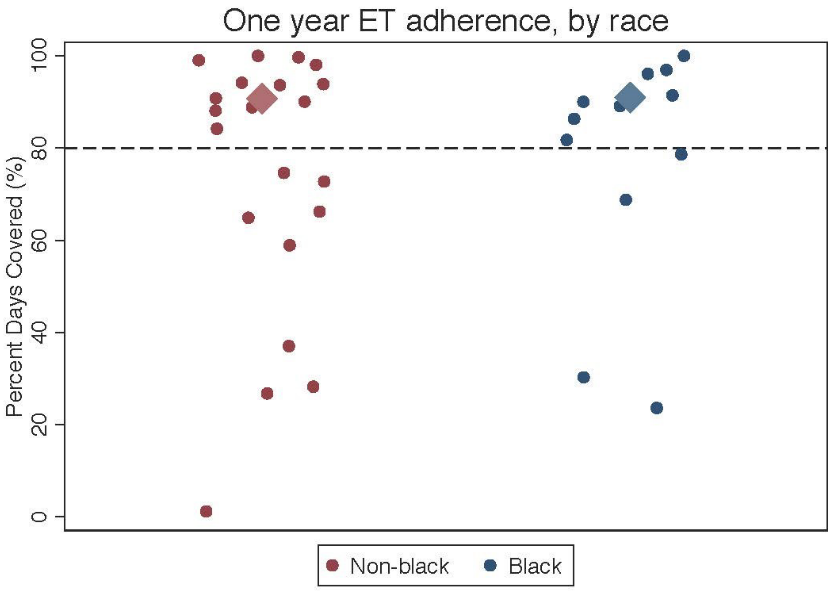

Table 1: GETSET Retention, Adherence, and Satisfaction

n (%)	Black	Non-Black	Overall
Recruitment	15	27	42
Completed 3/5 sessions	12 (80%)	24 (89%)	34 (81%)
**One-year outcomes (n=12 black, 23 non-black participants)**			
Self-reported adherence	11 (92%)	18 (78%)	29 (81%)
Satisfied with counseling overall	11 (92%)	18 (78%)	29 (83%)
Felt counseling was a good fit	11 (92%)	18 (78%)	29 (83%)

Among patients who have completed 12-month follow-up to date, participant retention, MI session participation, self-reported adherence, satisfaction, and acceptability were high (Table 1). Satisfaction and acceptability of the intervention among black women was particularly high. Although limited by small sample sizes, these preliminary data suggest that MI is a feasible and acceptable approach to promote ET adherence in diverse women.

1.5.3 Use of Text Message Reminders to Support ET Adherence

Text message reminders also support ET use. Drs. Electra Paskett and Michelle Naughton, along with Dr. Doug Post, now deceased, were awarded an Alliance Pilot Project to develop a single-arm text-based reminder intervention for ET adherence, conducted at five Alliance sites. Focus groups of ET patients and interviews with ET-prescribing physicians were used, along with a literature review, to develop a TMR protocol consisting of a 12-week daily TMR intervention with weekly interactive adherence monitoring, delivered by a smart phone app. The team created an educational video that participants viewed at the start of the intervention.

The video featured breast oncologists and breast cancer patients and discusses the importance of adherence and communication with the health care team about symptoms/challenges associated with ET, addresses barriers to adherence, and teaches women how to use PACE (Presenting detailed information, Asking questions, Checking your understanding, Expressing concerns) communication skills when communicating with their health care provider (e.g. how to discuss side effects) [49-52]. Thirty-nine participants were enrolled, and 27 (69.2%) completed all study requirements. Significant improvements were observed for self-reported medication adherence (p=0.02), mental health (p=0.007), and perceived stress (p=0.04). Patients (91.9%) and physicians (100%) agreed that patient participation in the TMR intervention was beneficial, easy to complete the intervention on the phone, and an overall positive experience [53].

Currently, the team at the Ohio State University (OSU) is recruiting for a follow-up study to this Alliance pilot, funded by the OSU Comprehensive Cancer Center Pelotonia funds. This is a randomized trial of breast cancer survivors who have been prescribed ET for at least 12 months, but who take their medication 6 days or less on average every week. These women are randomized to TMR (n=120) or usual care (n=120) for 4 months. Pillsy caps are being used in each arm to measure adherence. In part, this study will be used to develop and test additional TMR daily reminder slogans, as well as refine participant monitoring procedures during the conduct of the intervention. In sum, our preliminary studies reflect our extensive collective expertise in understanding the complex and multifactorial determinants of ET adherence in diverse breast cancer patient populations. This work positions us exceedingly well to conduct this investigation, which will extend our preliminary studies to elucidate the efficacy of TMR and MI in a randomized setting, to understand impact of these behavioral interventions on patient-reported outcomes (PROs), and to assess the economic efficiency of these interventions.

1.5.4 Use of Patient-Facing Components

Both pilot studies described above included an educational video about ET and the intervention, as well as additional patient materials, including an evidence-based resource guide. All materials were pre-tested in multiple focus groups for acceptability and cultural appropriateness. However, to fully capture both the MI and TRM intervention components in a single educational video, for this RCT, we will create a new educational video and rework our resource guide to encompass additional educational content and be website-friendly. As done in our pilot studies, the video will feature racially- and age-diverse simulated patients and use representative quotes from real patient experiences; the video introduction will make clear that the patient actors represent the views and statements of patients but are not actual patients.

Our patient-facing educational materials and online patient website will be developed in partnership with the Communication for Health Applications and Interventions (CHAI) Core, a resource of the UNC-Lineberger Comprehensive Cancer Center with expertise in design of patient-facing materials for intervention studies. The CHAI Core will develop the study website accessible to participants, providers and research staff, including an online copy of the workbook used in MI counseling sessions (for patients in MI study arms), and other general ET educational resources (for all participants). Participants will also be able to find relevant contact information specific to their assigned arm on the website for patients to reach out to the study team if help is needed. Access to website content will be gated by a participant login during the study, with the ability to open the website to the public at study completion as a dissemination tool. Participants will be informed that website use data is collected in an aggregated fashion to facilitate website improvements.

1.5.5 Use of Medication Event Monitoring Systems (Pillsy) Caps

Medication Event Monitoring Systems caps have been used extensively in other disease settings to understand daily pill-taking behavior by using technology that monitors opening and closing of pill bottles [54, 55]. This passive medication monitoring system is an improvement

over daily pill diaries, self-reported recall, and pharmacy or claims-based refill data in that self-reported measures may be compromised by social desirability bias and forgetfulness, whereas refill data are limited in the disconnect between medication refills and actual pill consumption [56]. Although there is no gold standard for medication adherence, Pillsy caps are arguably a more objective and rigorous adherence metric [57].

1.5.6 Provision of Smart Phones for Eligible Participants without Access

For participants who do not have access to a smart phone, the study will provide phones and service via the OSU's contract with Verizon Wireless. After the consent/registration visit, but prior to the initiation/baseline visit, the smart phone will be registered and activated by the OSU project manager and mailed to the site coordinator. Smartphones will be delivered within 7 days of randomization. At the initiation/baseline visit, the site coordinators will orient the participant to the device, and ask them to complete several tasks on their phone to ensure that the phone is operating properly.

For the OSU-provided phones, charges for participant's service and data plan will only be paid through the end of the intervention period at 12 months. The study will not pay for extra applications, other equipment, or additional lines. At the end of the 12-month intervention period, patients will be responsible for paying monthly fees if continued service is desired. Near the end of the 12 month period, the OSU staff will work with the patients to remind them of the expiring phone plan and will explain options for continuing service. The actual phones, however, will belong to patients at the end of 12 months and do not need to be returned to study staff.

1.6 Study Design

We plan a multi-site randomized trial using a four arm intervention design. All participants will be randomized equally to one of four arms: enhanced usual care, text message reminder intervention only, motivational interviewing counseling intervention only, or TMR + MI intervention. Randomization will be stratified by race and age to ensure balance between arms. We will use the interventions developed in our pilot work for the basic components of the three intervention arms, and usual care will consist of basic endocrine therapy educational content (via clinic and study website) provided to patients at medication initiation. Participants randomized to an intervention arm will actively participate in the intervention for approximately 9 months, but all participants will be followed for adherence and patient-reported outcomes (PRO) endpoints at 12 and 24 months. PRO surveys will be collected via the Alliance e-PRO survey administration system at baseline, 3-, 6-, 12-, 18-, and 24- months, and Pillsy cap data will be collected continuously via the Pillsy app on the patient's smart phone. The primary analysis will compare ET adherence in women exposed to TMR-only, MI-only, or TMR+MI interventions versus usual care. We will recruit approximately 263 patients to each arm, for a total of no less than 1052 participants. With an assumed loss-to-follow-up of approximately 11%, we will over recruit for a total of 1181 patients enrolled.

2.0 OBJECTIVES

2.1 Primary objective

Compare ET adherence in diverse women exposed to text message reminders (TMR)-only, telephone-based motivational interviewing counseling (MI)-only, or both (TMR+MI), versus usual care.

2.2 Secondary objective

2.2.1 Evaluate the effects of TMR, MI and TMR+MI interventions on secondary patient-reported outcomes, including medication use self-efficacy (MUSE), health related quality of life (HRQOL), cancer worry, and knowledge and attitudes about ET, relative to usual care.

2.2.2 Describe the incremental resources used and value of delivering TMR, MI, and TMR+MI interventions, relative to usual care.

2.3 Exploratory objectives

Difference in disease-free survival including DCIS (DFS-DCIS), to be defined according to standard definition as patients free of invasive ipsilateral recurrence, local/regional invasive recurrence, distant recurrence, death from any cause, invasive contralateral breast cancer, ipsilateral or contralateral DCIS, or second primary invasive non-breast cancer), between patients randomized to the TMR-only, MI-only, and TMR+MI interventions relative to usual care [58]. Additionally, the pattern of accrual on Black and younger patients will be assessed at the initial 50% of accrual enrolled and at the end of the trial.

3.0 PATIENT SELECTION

For questions regarding eligibility criteria, see the Study Resources page. Please note that the Study Chair cannot grant waivers to eligibility requirements.

3.1 On-Study Guidelines

This clinical trial can fulfill its objectives only if patients appropriate for this trial are enrolled. All relevant medical and other considerations should be taken into account when deciding whether this protocol is appropriate for a particular patient. Physicians should consider the risks and benefits of any therapy, and therefore only enroll patients for whom this treatment is appropriate.

Physicians should consider whether any of the following may render the patient inappropriate for this protocol:

- Patients with a contraindication to endocrine therapy medications, such as existing or planned pregnancy

3.2 Eligibility Criteria

Use the spaces provided to confirm a patient's eligibility by indicating Yes or No as appropriate. It is not required to complete or submit the following page(s).

When calculating days of tests and measurements, the day a test or measurement is done is considered Day 0. Therefore, if a test were done on a Monday, the Monday one week later would be considered Day 7.

___ **3.2.1 Women with an initial pathologically confirmed diagnosis of stage I-III, hormone receptor positive, HER2-neu negative, invasive breast cancer within 18 months (i.e., ≤548 days) prior to registration**

- If a patient has undergone neo-adjuvant chemotherapy and had no residual invasive disease at the time of surgery, eligibility can be based on clinical stage I-III prior to treatment and pathologic confirmation of receptor status from a diagnostic biopsy.
- Hormone receptor positive is defined as estrogen receptor (ER), progesterone receptor (PR), or both of >1%
- HER2-neu negative is defined as 0-1+ by ImmunoHistoChemical (IHC) analysis, or non-amplified by Fluorescence in situ Hybridization (FISH) analysis. If HER2-neu status is unknown due to insufficient tissue for evaluation, the patient is eligible.

- Patients with synchronous primary tumor foci of different receptor phenotypes, whether in the same or contralateral breast, may be enrolled as long as one tumor focus meets the receptor criteria and the patient is otherwise eligible.

___ **3.2.2 Prior Treatment:**

- Patients must have completed all planned cancer-directed surgery (except reconstruction surgery or oophorectomy).
- Patients must have completed all other adjuvant therapy (e.g., radiation and IV or oral chemotherapy) prior to registration (see Section 3.2.3 regarding adjuvant endocrine therapy).
- Patients who will be taking a CDK4/6 inhibitor (e.g., abemaciclib, palbociclib, or ribociclib) during endocrine therapy are NOT permitted to enroll as this is considered ongoing adjuvant therapy.
- Patients who have previously been on ET drug outside the 6-month window for any reason, including breast cancer prevention or high-risk non-malignant lesions (e.g., ADH, LCIS) are ineligible.

___ **3.2.3 Patients must be taking a once daily endocrine therapy drug initiated within the 6 months (i.e., ≤183 days) prior to registration OR have received a prescription for a once daily ET medication with stated intent to initiate within 6 weeks (i.e., ≤42 days) after registration.**

- Patients who switch ET drugs prior to enrollment remain eligible as long as the total time since initiation of the first ET drug does not exceed 6 months prior to registration.
- Patients who have stopped their ET drug prior to enrollment and not started another ET drug are ineligible.

___ **3.2.4 No history of previous cancer as follows:**

- Invasive or non-invasive breast cancer at any time
- Non-breast cancer, within the past 5 years, excluding non-melanoma skin cancer
- Patients with a history of high-risk breast lesions (e.g., atypical ductal hyperplasia – ADH, lobular carcinoma in situ—LCIS) are eligible as long as they have not previously taken an endocrine therapy drug (see Section 3.2.3).

___ **3.2.5 Patients must be willing to use a smart phone for study activities**

- Patient is NOT to be deemed ineligible during the recruitment process if they do not have a smart phone.
- A smart phone and service can be provided to the participant at no cost for the duration of the study activities if the participant meets at least one of the following criteria: (1) does not own a smart phone, (2) has a limited data, minutes, or texting plan, or (3) their smart phone cannot support the Alliance ePRO survey app. Study-provided smart phones will be provided through the Ohio State University partnership with Verizon Wireless.
- The Clinical Research Professional (CRP) is ONLY to discuss this option with those patients who self-identify a phone-related barrier to participation, including: lack of a

smart phone, insufficient phone plan (minutes/text/data), or a smart phone incompatible with the Alliance ePRO app.

___ **3.2.6 Patients must be willing to use a Pillsy medication event monitoring system for the duration of study participation**

- Patients must be willing to use a pill bottle (provided by either the study or the patient's pharmacy) in combination with the Pillsy medication event monitoring system each time ET drug is taken. Use of any other container (e.g., pill box, etc.) for ET drug during study period is not permitted.

___ **3.2.7 Age ≥ 18 years**

___ **3.2.8 Language: In order to complete the mandatory patient-completed measures, participants must be able to speak and read English.**

___ **3.2.9 Co-enrollment is allowed with the permission of the Alliance Executive Officer and both studies' Study Chairs.**

4.0 PATIENT REGISTRATION

4.1 Investigator and Research Associate Registration with CTEP

Food and Drug Administration (FDA) regulations and National Cancer Institute (NCI) policy require all individuals contributing to NCI-sponsored trials to register and renew their registration annually. To register, all individuals must obtain a Cancer Therapy Evaluation Program (CTEP) Identity and Access Management (IAM) account at https://ctepcore.nci.nih.gov/iam. In addition, persons with a registration type of Investigator (IVR), Non-Physician Investigator (NPIVR), or Associate Plus (AP) must complete their annual registration using CTEP's web-based Registration and Credential Repository (RCR) at https://ctepcore.nci.nih.gov/rcr.

RCR utilizes five person registration types.

- IVR — MD, DO, or international equivalent;
- NPIVR — advanced practice providers (e.g., NP or PA) or graduate level researchers (e.g., PhD);
- AP — clinical site staff (e.g., RN or CRA) with data entry access to CTSU applications such as the Roster Update Management System [RUMS], OPEN, Rave, acting as a primary site contact, or with consenting privileges;
- Associate (A) — other clinical site staff involved in the conduct of NCI-sponsored trials; and
- Associate Basic (AB) — individuals (e.g., pharmaceutical company employees) with limited access to NCI-supported systems.

RCR requires the following registration documents:

Documentation Required	IVR	NPIVR	AP	A	AB
FDA Form 1572	✓	✓			
Financial Disclosure Form	✓	✓	✓		
NCI Biosketch (education, training, employment, license, and certification)	✓	✓	✓		
GCP training	✓	✓	✓		
Agent Shipment Form (if applicable)	✓				
CV (optional)	✓	✓	✓		

An active CTEP-IAM user account and appropriate RCR registration is required to access all CTEP and Cancer Trials Support Unit (CTSU) websites and applications. In addition, IVRs and NPIVRs must list all clinical practice sites and Institutional Review Boards (IRBs) covering their practice sites on the FDA Form 1572 in RCR to allow the following:

- Addition to a site roster;
- Assign the treating, credit, consenting, or drug shipment (IVR only) tasks in OPEN;
- Act as the site-protocol Principal Investigator (PI) on the IRB approval; and
- Assign the Clinical Investigator (CI) role on the Delegation of Tasks Log (DTL).

In addition, all investigators acting as the Site-Protocol PI (investigator listed on the IRB approval), consenting/treating/drug shipment investigator in OPEN, or as the CI on the DTL must be rostered at the enrolling site with a participating organization.

Additional information is located on the CTEP website at https://ctep.cancer.gov/investigatorResources/default.htm. For questions, please contact the **RCR Help Desk** by email at RCRHelpDesk@nih.gov.

4.2 CTSU Site Registration Procedures

Permission to view and download this protocol and its supporting documents is restricted and is based on person and site roster assignment housed in the CTSU Regulatory Support System (RSS).

This study is supported by the NCI Cancer Trials Support Unit (CTSU).

IRB Approval

For CTEP and Division of Cancer Prevention (DCP) studies open to the National Clinical Trials Network (NCTN) and NCI Community Oncology Research Program (NCORP) Research Bases after March 1, 2019, all U.S.-based sites must be members of the NCI Central Institutional Review Board (NCI CIRB). In addition, U.S.-based sites must accept the NCI CIRB review to activate new studies at the site after March 1, 2019. Local IRB review will continue to be accepted for studies that are not reviewed by the CIRB, or if the study was previously open at the site under the local IRB. International sites should continue to submit Research Ethics Board (REB) approval to the CTSU Regulatory Office following country-specific regulations.

Sites participating with the NCI CIRB must submit the Study Specific Worksheet (SSW) for Local Context to the CIRB using IRBManager to indicate their intent to open the study locally. The NCI CIRB's approval of the SSW is automatically communicated to the CTSU Regulatory Office, but

sites are required to contact the CTSU Regulatory Office at CTSURegPref@ctsu.coccg.org to establish site preferences for applying NCI CIRB approvals across their Signatory Network. Site preferences can be set at the network or protocol level. Questions about establishing site preferences can be addressed to the CTSU Regulatory Office by email or calling 1-888-651-CTSU (2878).

In addition, the Site-Protocol Principal Investigator (PI) (i.e. the investigator on the IRB/REB approval) must meet the following criteria in order for the processing of the IRB/REB approval record to be completed:

- Holds an active CTEP status;
- Active status at the site(s) on the IRB/REB approval (*applies to US and Canadian sites only*) on at least one participating organization's roster;
- If using NCI CIRB, active on the NCI CIRB roster under the applicable CIRB Signatory Institution(s) record;
- Includes the IRB number of the IRB providing approval in the Form FDA 1572 in the RCR profile;
- Lists all sites on the IRB/REB approval as Practice Sites in the Form FDA 1572 in the RCR profile; and
- Holds the appropriate CTEP registration type for the protocol.

4.2.1 Additional site registration requirements

Additional site requirements to obtain an approved site registration status include:

- An active Federal Wide Assurance (FWA) number;
- An active roster affiliation with the Lead Protocol Organization (LPO) or a Participating Organization (PO);
- An active roster affiliation with the NCI CIRB roster under at least one CIRB Signatory Institution (*US sites only*); and
- Compliance with all protocol-specific requirements (PSRs) (See Section 12.0).

4.2.2 Downloading Site Registration Documents

Download the site registration forms from the protocol-specific page located on the CTSU members' website. Permission to view and download this protocol and its supporting documents is restricted to institutions and its associated investigators and staff on a participating roster. To view/download site registration forms:

- Log in to the CTSU members' website (https://www.ctsu.org) using your CTEP-IAM username and password;
- Click on *Protocols* in the upper left of the screen:
    - Enter the protocol number in the search field at the top of the protocol tree; or
    - Click on the By Lead Organization folder to expand, then select *Alliance*, and protocol number *A191901*.
- Click on *Documents, Protocol Related Documents, and* use the *Document Type* filter and select *Site Registration to* download and complete the forms provided. (Note: For sites under the CIRB, IRB data will load automatically to the CTSU.)

4.2.3 Submitting Regulatory Documents

Submit required forms and documents to the CTSU Regulatory Office using the Regulatory Submission Portal on the CTSU members' website.

To access the Regulatory Submission Portal log in to the CTSU members' website, go to the *Regulatory* section and select *Regulatory Submission*.

Institutions with patients waiting that are unable to use the Regulatory Submission Portal should alert the CTSU Regulatory Office immediately by phone or email: 1-866-651-CTSU (2878), or CTSURegHelp@coccg.org in order to receive further instruction and support.

4.2.4 Checking Site's Registration Status

Site registration status may be verified on the CTSU members' website.

- Click on *Regulatory* at the top of the screen;
- Click on *Site Registration; and*
- Enter the sites 5-character CTEP Institution Code and click on Go.
    - Additional filters are available to sort by Protocol, Registration Status, Protocol Status, and/or IRB Type.

Note: The status shown only reflects institutional compliance with site registration requirements as outlined within the protocol. It does not reflect compliance with protocol requirements for individuals participating on the protocol or the enrolling investigator's status with NCI or their affiliated networks.

4.3 Patient Registration Requirements

4.3.1 Informed consent

The patient must be aware of the neoplastic nature of her disease and willingly consent after being informed of the procedure to be followed, the experimental nature of the therapy, alternatives, potential benefits, side-effects, risks, and discomforts. Current human protection committee approval of this protocol and a consent form is required prior to patient consent and registration.

Patients with impaired decision making capacity may be enrolled on this study, if they are capable of providing informed consent without assistance from a proxy.

4.3.2 Patient-reported outcomes

Electronic patient reported outcomes (ePRO): This study includes the use of ePRO, (electronic patient-reported outcomes). After the patient is registered to the trial, the CRA will complete a second registration to the Patient Cloud. The CRA will create a unique patient registration code by accessing the Patient Cloud through iMedidata Rave. Patients (with assistance from CRAs) will need to download the Patient Cloud ePRO app on their own device and use the unique registration code given by the CRA to create an account. Once completed, the patient will be able to complete the submission of patient reported outcomes electronically.

Prior to registration, the patient should be asked about the availability of an electronic device and willingness to complete the patient-reported questionnaires on the device (see Section 3.2.5). The patient should be informed that ePRO must be used by the patient throughout the duration of the study for all time points. See Appendix XV for further instructions on setting up ePRO.

4.3.3 Protected health information

For local site HIPAA documentation, please note that the following personal health information (PHI) may be released outside of the Alliance.

Within 48 hours after patient randomization, sites must submit the "Patient Mailing and Contact Information Form," that can be found on the A191901 study page of the Alliance and CTSU websites, and securely fax to GET SET study staff at the OSU. The contact information form will be destroyed upon completion of the study. From this form, patients' names, mailing addresses, mobile phone numbers, mobile phone carriers, alternate phone numbers, MMS domain, and assigned randomized study arms will then be entered by OSU staff in the REDCap system at the OSU, housed within the Behavioral Measurement Shared Resource of the OSU Comprehensive Cancer Center, which Dr. Naughton now directs. REDCap data will be available to staff at both coordinating centers, OSU and UNC, to allow the separate intervention components to run independently. Site staff will re-verify the patients' contact information at every patient contact and send an updated form to OSU, in the event of a change. The OSU staff will update the REDCap data accordingly. All REDCap contact information data will be destroyed upon completion of the study.

4.4 Patient registration/randomization procedures

The Oncology Patient Enrollment Network (OPEN) is a web-based registration system available on a 24/7 basis. OPEN is integrated with CTSU regulatory and roster data and with the LPOs registration/randomization systems or the Theradex Interactive Web Response System (IWRS) for retrieval of patient registration/randomization assignment. OPEN will populate the patient enrollment data in NCI's clinical data management system, Medidata Rave.

Requirements for OPEN access:

- A valid CTEP-IAM account;
- To perform enrollments or request slot reservations: Must be on an LPO roster, ETCTN corresponding roster, or participating organization roster with the role of Registrar. Registrars must hold a minimum of an Associate Plus (AP) registration type;
- If a Delegation of Tasks Log (DTL) is required for the study, the registrars must hold the OPEN Registrar task on the DTL for the site; and
- Have an approved site registration for the protocol prior to patient enrollment.

To assign an Investigator (IVR) or Non-Physician Investigator (NPIVR) as the treating, crediting, consenting, drug shipment (IVR only), or receiving investigator for a patient transfer in OPEN, the IVR or NPIVR must list the IRB number used on the site's IRB approval on their Form FDA 1572 in RCR. If a DTL is required for the study, the IVR or NPIVR must be assigned the appropriate OPEN-related tasks on the DTL.

Prior to accessing OPEN, site staff should verify the following:

- Patient has met all eligibility criteria within the protocol stated timeframes; and
- All patients have signed an appropriate consent form and Health Insurance Portability and Accountability Act (HIPAA) authorization form (if applicable).

Note: The OPEN system will provide the site with a printable confirmation of registration and treatment information. You may print this confirmation for your records.

Access OPEN at https://open.ctsu.org or from the OPEN link on the CTSU members' website. Further instructional information is in the OPEN section of the CTSU website at https://www.ctsu.org or https://open.ctsu.org. For any additional questions, contact the CTSU Help Desk at 1-888-823-5923 or ctsucontact@westat.com.

4.5 Stratification Factors and Treatment Assignments

The "Patient Mailing and Contact Information" form (Appendix I) completed prior to registration will assist sites in determining stratification factors listed below. Information from the baseline questionnaire will be required for registration and randomization.

- Race (Black vs. Other) (Appendix I, Question 8)
  - Patient will be categorized as Black if they report their race to be only Black or Black in combination with any other race on the Baseline Questionnaire
  - All other patients will be categorized as "Other" (including white, Asian, etc.)
- Age (<50 years vs. 50 or older) (Appendix I, Question 7)
  - Patients will be categorized as <50 years or as 50 or older based on self-reported age in years at the time of baseline questionnaire

The randomization routine is found in Section 11.0 (Statistical Considerations).

5.0 STUDY CALENDAR

Laboratory and clinical parameters during treatment are to be followed using individual institutional guidelines and the best clinical judgment of the responsible physician. It is expected that patients on this study will be cared for by physicians experienced in the treatment and supportive care of patients on this trial.

	At Registration*	Months 3 & 6	Month 12	Month 18	Month 24
**Tests & Observations**					
Patient Mailing & Contact Information Form (Appendix I)	X(1)				
Current Medication	X(2)		X(2)		X(2)
Baseline Patient Questionnaire	X				
Follow-up Patient Questionnaire**		X	X	X	X
Pillsy Cap Data		X(3)			
Recurrence/New Cancers Information			X		X
**Staging**					
Documentation of Diagnosis/History	X (4)				
Documentation of DFS event			X (5)		X (5)

* May be completed any time following registration and prior to start of intervention.

** See Section 9.0 for details on the individual measures included in the Follow-up Patient Questionnaire.

1. Form must be completed and securely faxed to OSU within 48 hours after registration. See Section 4.3.3.
2. List of current medications will be abstracted by clinic staff from patient medical record. Update current medications at Months 12 and 24 to include any new medications that the participant begins while on study, including any change in endocrine therapy medication, and any medications that may have been stopped while on study.
3. Pillsy cap data collection will occur continuously and in real time through the Pillsy app on the participant's smartphone after it is activated at the baseline visit; If needed during study participation, participants will be asked to bring their Pillsy Cap to the local clinic or return via mail to the central coordinating center to allow for manual data collection. See Section 9.10.1 for more information.
4. See Section 6.1.4 for additional information.
5. See Section 6.1.4 for list of DFS events.

6.0 DATA AND SPECIMEN SUBMISSION

6.1 Data Collection and Submission

Data collection for this study will be done through Medidata Rave clinical data management system, the Alliance ePRO survey app (for all patient-reported outcome data), and the Pillsy Adherence Software (for all Pillsy caps data collection).

6.1.1 Data submission schedule

A Schedule of Forms is available on the Alliance study webpage, within the Case Report Forms section. The Schedule of Forms is also available on the CTSU site within the study-specific Education and Promotion folder, and is named Time & Events.

6.1.2 Medidata Rave

Medidata Rave is a clinical data management system being used for data collection for this trial/study. Access to the trial in Rave is controlled through the CTEP-IAM system and role assignments.

Requirements to access Rave via iMedidata:

- A valid CTEP-IAM account; and
- Assigned a Rave role on the LPO or PO roster at the enrolling site of: Rave CRA, Rave Read Only, Rave CRA (LabAdmin), Rave SLA, or Rave Investigator.

Rave role requirements:

- Rave CRA or Rave CRA (Lab Admin) role must have a minimum of an Associate Plus (AP) registration type;
- Rave Investigator role must be registered as an Non-Physician Investigator (NPIVR) or Investigator (IVR); and
- Rave Read Only role must have at a minimum an Associates (A) registration type.

Refer to https://ctep.cancer.gov/investigatorResources/default.htm for registration types and documentation required.

Upon initial site registration approval for the study in Regulatory Support System (RSS), all persons with Rave roles assigned on the appropriate roster will be sent a study invitation email from iMedidata. To accept the invitation, site staff must either click on the link in the email or log in to iMedidata via the CTSU members' website under *Data Management* > *Rave Home* and click to *accept* the invitation in the *Tasks* pane located in the upper right corner of the iMedidata screen. Site staff will not be able to access the study in Rave until all required Medidata and study specific trainings are completed. Trainings will be in the form of electronic learnings (eLearnings) and can be accessed by clicking on the eLearning link in the *Tasks* pane located in the upper right corner of the iMedidata screen. If an eLearning is required for a study and has not yet been taken, the link to the eLearning will appear under the study name in the *Studies* pane located in the center of the iMedidata screen; once the successful completion of the eLearning has been recorded, access to the study in Rave will be granted, and a *Rave EDC* link will replace the eLearning link under the study name.

Site staff that have not previously activated their iMedidata/Rave account at the time of initial site registration approval for the study in RSS will receive a separate invitation from iMedidata to activate their account. Account activation instructions are located on the CTSU website in the Data Management section under the Rave resource materials (Medidata Account Activation and Study Invitation Acceptance). Additional information on iMedidata/Rave is available on the CTSU members' website in the Data Management > Rave section at www.ctsu.org/RAVE/

or by contacting the CTSU Help Desk at 1-888-823-5923 or by email at ctsucontact@westat.com.

6.1.3 Data Quality Portal

The Data Quality Portal (DQP) provides a central location for site staff to manage unanswered queries and form delinquencies, monitor data quality and timeliness, generate reports, and review metrics.

The DQP is located on the CTSU members' website under Data Management. The Rave Home section displays a table providing summary counts of Total Delinquencies and Total Queries. DQP Queries, DQP Delinquent Forms, DQP Form Status and the DQP Reports modules are available to access details and reports of unanswered queries, delinquent forms, forms with current status and timeliness reports. Review the DQP modules on a regular basis to manage specified queries and delinquent forms.

The DQP is accessible by site staff that are rostered to a site and have access to the CTSU website. Staff that have Rave study access can access the Rave study data using a direct link on the DQP.

To learn more about DQP use and access, click on the Help icon displayed on the Rave Home, DQP Queries, DQP Delinquent Forms, DQP Form Status, and DQP Reports modules.

6.1.4 Supporting documentation to be submitted to the Alliance

This study requires supporting documentation for diagnosis, type of endocrine therapy initially prescribed and date of endocrine therapy prescription, invasive ipsilateral recurrence, local/regional invasive recurrence, distant recurrence, death from any cause, invasive contralateral breast cancer, ipsilateral or contralateral DCIS, or second primary invasive non-breast cancer. Supporting documentation will include clinic notes, prescriptions, pathology, surgery, and radiology reports. These must be submitted at the following time points:

- **At registration:** (a) pathology reports documenting patient's estrogen, progesterone and HER2 receptor status and final pathologic stage (or clinical stage if patient has had neoadjuvant chemotherapy with a complete pathologic response); (b) clinic note OR other medical record documentation by provider OR medication prescription documenting type and date of endocrine therapy prescribed (any one of these forms of documentation of endocrine therapy prescription is acceptable).
- **At time of DFS event:** pathology and radiology reports.

Note: Documentation of initial breast cancer diagnosis via clinic note is acceptable. Include pathology report that shows HR+ and HER2-neu negativity and confirmation of treatment within 18 months prior to enrollment. Description of prior treatment and future treatment plans (confirmation that no further treatment will be given alongside ET). Clinic note describing any past history of cancer. Clinic note or pharmacy prescription confirming ET prescription received within 6 months prior to registration.

All supporting documents must be de-identified, per institutional policy. Supporting documentation must be submitted via Rave.

6.2 Submission of Patient Completed Measures

The patient completed measures for this study (see Section 9.0), are available in English only. Participation in Alliance A191901 is restricted to patients who are able to speak, understand, and read English.

The data from the patients' responses are submitted directly from the device into the Rave database. There are no documents to audit. The electronic responses are the source documentation.

7.0 INTERVENTION

Participants will be randomized 1:1:1:1 to one of four arms: enhanced usual care, text message reminder intervention only, motivational interviewing counseling intervention only, or TMR + MI intervention. All intervention components will be delivered centrally, either by the Ohio State University (TMR component) or the University of North Carolina Chapel Hill (MI component). All components of the intervention will be delivered over a 9-month time period, and participants will continue to be followed for up to 24 months. Sites will be responsible for enrolling participants and collecting data, as described in Section 6.0.

In order to remain on trial, study activities must be completed within the following windows:

- Participants who have not yet started an endocrine therapy drug but have a prescription and stated an intent to initiate treatment, must initiate the prescribed drug ≤6 weeks of randomization.
- All participants must complete the Initiation & Baseline visit (see Section 7.2) ≤6 weeks of randomization.
- All participants must initiate intervention activities ≤8 weeks of randomization, regardless of endocrine therapy initiation status at the time of registration.

Intervention activity initiation is defined as follows for each active treatment arm:

- Arm 1 – receipt of the first text message
- Arm 2 – completion of the first telephone counseling session
- Arm 3 – receipt of first text message and completion of first telephone counseling session

Participants who have not confirmed drug initiation to the coordinator within 6 weeks of randomization, participants who have not completed the Initiation & Baseline visit within 6 weeks of randomization, and participant in an active treatment arm who have not begun all activities in the allocated intervention within ≤8 weeks of randomization, for any reason, will be withdrawn. Requests for a window extension may be granted by a study PI for any participants who do not complete the specified study activities within these windows. Requests will be considered on a case-by-case basis and should be submitted to the central study project manager and/or Study Chair as soon as possible.

For questions regarding the intervention, please see the study contacts page.

7.1 Screening & Informed Consent Visit

A checklist for the screening and informed consent visit will be provided to the site study coordinators to help ensure all steps are completed, and appropriate data are collected.

Screening

Participants will be recruited by clinical research coordinators at participating sites, including main member and NCI Community Oncology Research Program (NCORP) network sites of the Alliance.

Remote screening and recruitment will be allowed to reduce burden and the number of required in-clinic visits. As is preferred by each individual institution, patients may be recruited in a combination of ways, including in-person clinic visits or remote recruitment via telephone, email and/or mailed letters, using clinically available contact information. Study coordinators at each site will use the "Remote Recruitment Scripts" (Appendix XX) to guide these interactions with patients. Patients will be contacted by the site research coordinator and a time will be scheduled at the patient's convenience to complete the informed consent process, either by in-clinic visit or via remote consent procedures. Patients will be informed of the study aims and requirements with the aid of the consent form and invited to participate.

To aid sites with their recruitment procedures and screening process, the study team will conduct monthly teleconferences with site coordinators to help identify and troubleshoot issues as they arise.

Patient consent and registration visit

Participants may sign consent if they have received a prescription for ET and indicate that they plan to initiate within 6 weeks but have not yet taken their first dose; however, intervention activities and outcome assessment will begin only after ET initiation is confirmed by the participant. Study coordinators at the site will verify ET initiation by telephone or in person contact with the patient on a weekly basis. After confirming ET initiation, study coordinators will enter the date of ET initiation into Rave. If the study coordinator does not confirm ET initiation with the participant within 6 weeks of randomization, the patient will be withdrawn (See Section 10.3.2). Participants will be instructed by the site coordinator to bring their pharmacy-provided prescription bottle to the initiation visit.

Remote consent: Remote consenting is allowable if meeting a patient in person is not feasible. The site may follow The National Cancer Institute's CIRB Remote Consenting Procedures to implement a remote consenting process. The site research coordinator may use the "Telephone Consent Script" (Appendix XXI) as a guide.

Smartphone assessment: At the time of the informed consent and registration visit, site coordinators will assess whether the participants possess a smartphone device that can receive text messages and support the Alliance electronic patient reported outcomes (ePRO) survey and Pillsy apps. If a participant is consented remotely, the site research coordinator will ask if the participant owns and uses a smartphone that would comply with the study activities. For participants who do not have access to a smartphone and are willing to enroll and be randomized to the protocol, a smart phone and service will be provided via a contract that OSU has with Verizon Wireless.

Patient Mailing and Contact Information Form: After obtaining written informed consent, the site coordinator will ask the participant to fill out the "Patient Mailing and Contact Information Form" (Appendix I, see Section 4.3.3 for more details), and enter the self-reported race and age into Rave. Patients will then be assigned a unique study identifier (see Section 7.3.2), and randomized to either usual care, TMR-only, MI-only, or TMR+MI.

If a participant is consented remotely, site research coordinators must wait to receive participant's signed consent form (e.g., via mail, fax or email), prior to conducting any research activities. After receiving signed consent form, site research coordinators will contact the participant via telephone to obtain the information on the "Patient Mailing and Contact Information Form."

Following the patient consent and registration visit

Patient Mailing and Contact Information Form (Appendix I): Within 48 hours of receiving the participant's randomization assignment, site coordinators will indicate the randomization assignment, unique study identifier and randomization date on the "Patient Mailing and Contact Information Form," and fax the form to the study staff at OSU.

Smart Pill Cap/Bottle Request: After the informed consent and registration visit, the site coordinator will complete the "Smart Pill Cap/Bottle Request Form," provided by the UNC coordinating center with the protocol-specific training documents (see Section 12.1.1) and will fax it to the central study project manager at the University of North Carolina – Chapel Hill. When filling out the request form, the "date needed at site" should be no more than 5 business days after the fax date. After receiving the requested form, the UNC central study staff will assign and ship the requested cap/bottle directly to the site. Devices will typically be delivered within 5-7 business days.

Urgent shipment requests (defined as any shipments with a "date needed at site" ≤4 business days after fax date) will be accommodated in extenuating circumstances. If a cap is needed at the site in ≤4 business days after the fax date, the site coordinator should: (1) fax the form to UNC following the standard procedures described above, and (2) send a supplemental email to the central study project manager and Study Chair that includes study participant ID, requested date of arrival, and a

reasonable justification for the urgent shipment request. Site staff will receive an email confirmation of urgent request prior to processing.

Smartphone Request Form (if needed): If a site coordinator deems that a participant is in need of a smart phone to complete study requirements, he/she will complete the "Smartphone Request Form", provided by the UNC coordinating center with the protocol-specific training documents (see Section 12.1.1), and the site will fax to the OSU project manager. After the consent/registration visit but prior to the initial/baseline visit, the smart phone will be registered and activated by the OSU Verizon Wireless representative and mailed to the site coordinator. Smartphones will be delivered prior to the participant's initiation/baseline visit after the receipt of the "Smartphone Request Form."

7.2 Initiation & Baseline Visit

After receiving the patient's randomization assignment, smart pill cap and bottle, hard copy of patient-facing materials and, if necessary, a study-provided smart phone, study coordinators will arrange for participants to return to clinic for an in-person initiation and baseline visit. The baseline visit must occur within 6 weeks of randomization date.

Hard copies of all patient-facing materials will be sent to the site for each registered participant and will be included with the Pillsy cap shipment. The patient-facing materials included will be based on the requested participant's randomization assignment. Study coordinators will provide these hardcopies to the participant at the Initiation & Baseline visit. If a participant requests a duplicate copy from study coordinators or the study coordinators need to print a copy of the materials, all patient-facing materials should be accessed from the GET SET website. Documents are available on the CTSU website for CIRB reference only.

If necessary, the site coordinator will set up the study-provided phones for those participants receiving one prior to the initiation/baseline visit. At the initiation/baseline visit, the site coordinators will have participants who receive a study-provided phone sign the "iPhone Participant Responsibilities and Agreement" form (Appendix XIX), which states that participants are agreeing to the terms and conditions of the Ohio State University provided smart phones. The site coordinator will fax the signed "iPhone Participant Responsibilities and Agreement" form to the study staff at OSU. Additionally, the site coordinator will go through the mechanics of the phone, if they are unfamiliar with its operation, and will ask the participants to complete several tasks on the smart phone to ensure that the phone is operating properly. **It should be noted,** charges for the OSU-provided phones' unlimited talk, text and data service plans will be paid for by the grant through the first 12 months of the study, while active interventions are administered. At no time, will the study pay for any apps or additional services such as subscriptions, downloads or media that have associated charges.

After the initiation/baseline visit, the OSU study staff will also follow-up with these participants prior to the initiation of the TMRs to ensure that the patients know how to operate the phones properly and to see if they have any questions about the provision of the phones.

At this visit, site coordinators will:

1. If applicable, orient participants to the study-provided smart phone and ensure proper functioning, and provide patient "iPhone User Guide" handout, that can be found on the A191901 study page of the Alliance and CTSU websites, if necessary;
2. Set up the ePRO app on ALL participants' smart phone devices (regardless of whether they have received a study-provided smartphone), and orient the patients to the app;
3. Allow participants to complete the baseline questionnaire in the ePRO app. This must be done prior to informing the participant of their randomization assignment.
4. Inform participants of randomization assignment;

5. Show participants the generalized study video and any applicable arm-specific study videos (all videos are located on the study website);
6. Orient participants to the participant website, including how to log in to the participant portal with arm-specific password, CHART health education resources, the FAQs/Contact Us page, and any arm-specific resources;
7. Give participants study-related materials, based on the participant's randomization assignment, and provided by the UNC coordinating center:
   - Arm 1 (TMR) participants should receive: the "Text Message Reminder (TMR) Patient Handout," the "Text Message Reminder (TMR) Group Information" sheet, and the "Tips for Using the Pillsy Smart Cap" sheet.
   - Arm 2 (MI) participants should receive: the "Telephone Counseling Patient Handout," the Telephone Counseling Workbook, and the "Tips for Using the Pillsy Smart Cap" sheet.
   - Arm 3 (TMR+MI) participants should receive: the "Text Message Reminder + Counseling Patient Handout," the "Text Message Reminder (TMR) Group Information" sheet, the Telephone Counseling Workbook, and the "Tips for Using the Pillsy Smart Cap" sheet.
   - Arm 4 (Enhanced Usual Care) participants should receive: the "Enhanced Usual Care Patient Handout," and the "Tips for Using the Pillsy Smart Cap" sheet.
8. Download the Pillsy app on the participant's smart phone device, using a provided instruction sheet, connect the smart pill cap to Pillsy app, and review the "Tips for Using the Pillsy Smart Cap" sheet with the patient.

All participants will receive a study-provided bottle that fits the smart pill cap. They may choose to use this bottle or their pharmacy-provided bottle to store their medication. The participant will be asked to bring their pharmacy-provided pill bottle to the initiation visit so that the site coordinator can place the synced pill cap on the bottle of the patient's choice. If the participant does not bring their pharmacy-provided pill bottle to the initiation visit, the site coordinator will instruct the participant on how to place the cap on the pharmacy-provided bottle at home. In addition, the Pillsy Information Sheet provides guidance on the smart pill cap, and is available for participants in print and electronically on the GET SET study website. For future prescription refills, participants will be instructed on how to transfer their cap to a new bottle, when needed, or to transfer their medication refill to the study bottle. Further, any patient who uses the study-provided bottle to store their medication will be coached on how to move their pharmacy-provided instruction label over to the new bottle.

In extenuating circumstances and only if necessary due to the rare occurrence of an extended period of internet outage at the institution or study smart phone application outage, study coordinators may remotely complete the ePRO app setup and viewing of the GET SET video with the participant. If study coordinators exercise this option, the study coordinator should: (1) call the patient and set up the Patient Cloud ("ePRO") app with them via phone or video conference and allow the participant time to complete the baseline questionnaire, (2) inform the participant of their randomization assignment only after the baseline questionnaire is complete, (3) confirm in real time the participant is able to find and start the video on the GET SET website, after completing the baseline questionnaire, (4) send the participant's study-related materials (handouts and workbook, if applicable) via mail or allow the participant to pick it up at the clinic, and (5) send an email to Central Study Manager notifying them of the extended internet outage and choice to use alternate protocol option. The setup of the Pillsy app may not be completed remotely. The participant must complete the baseline visit and begin intervention activities within the same windows as detailed in Section 7.0.

After the initiation and baseline visit, site coordinators will review medical charts to abstract pertinent cancer-specific information, including stage at diagnosis, grade, treatments received to date, comorbidities, and number and dosing of medications taken at baseline.

7.3 Text-Message Reminder (TMR) Component (Arms 1 and 3)

7.3.1 TMR Intervention Content

Daily Text Message Reminders: A library of 62 distinct messages has been created to ensure participants receive a new message every day with scheduled variation and recycling of messages (see Appendix XIV). The messages were designed to encourage medication adherence in a positive manner. Two types of messages will be utilized to address adherence behavior: (1) daily text message reminders sent to participants to take their ET medication; and (2) monthly questions, which help to provide accountability and resolve specific barriers through provision of pre-set motivational responses. The daily messaging will focus on three behaviors: initiation, continuation, and adherence to the prescribed dose, as appropriate.

Monthly Questions: Once per month, participants in the TMR arms will receive the following text message: "Over the past 7 days, on how many days did you take your ET [endocrine therapy medication]? ("Endocrine therapy medication" may be replaced by the brand or generic name of endocrine therapy used, if women prefer.) Responses will be entered as 0-7. A response of 6 and above will trigger a positive message that encourages patients to keep taking the medication (see Appendix XIV). If the participants indicate that they missed more than 1 dose over the past 7 days (i.e., took 5 or few doses that week), they will receive a message asking about reasons for missed doses, followed by tips to improve adherence. Follow-up messages specific to the most common barriers provide encouragement (e.g. "Taking this medication is an investment in your health") and actionable approaches to addressing these barriers (e.g. "Please talk to your doctor or nurse about your concerns"). A sample of the most common response options and subsequent system replies (actionable approach to addressing the barrier) can be found in the table in Appendix XIV.

Starting at month 3, participants who have reported at least 2 consecutive months of adherence problems and have received text message replies to talk with their health care team, will receive additional texts. These participants will be asked if they did contact their physicians/health care teams about their adherence issues. If a participant replies "yes", they will be asked if someone on their health care team provided information to assist them with their adherence problems. If participants did not contact their physicians/health care teams, we will ask them why they did not contact them, and refer the patients back to the "Text Message Reminder (TMR) Group Information" handout that was provided on study entry. This handout describes the PACE principles (Presenting detailed information, Asking questions, Checking your understanding, and Expressing concerns), and all of the initial text messages are based on PACE, with the most common response being to talk directly with their health care team (Cegala et al., 2000; 2001). We will not complete any direct back-and-forth counseling with the patients, as it will confound the MI arms of the study.

7.3.2 TMR Intervention Delivery

Each participant's smart phone number and cell phone carrier domain will be entered into the REDCap system at OSU, which uses unique participant identifiers to send text messages in a non-identifiable manner. Alternative phone numbers will also be collected in case of message delivery failure.

Following the initiation/baseline visit, the team at OSU will send an initial text message to all participants randomized to the TMR- based arms to welcome them to the study and ask participants to verify the receipt of the message by answering a single survey question in REDCap. If the OSU Project Coordinator does not receive a confirmation of receipt within approximately 2-3 days, the project coordinator will reach out to the participant directly to

troubleshoot any technical issues. Participants experiencing difficulties in receiving the text messages, will work with the OSU Project Manager individually to resolve any problems. The OSU project manager will continue to work with the patients during the intervention to troubleshoot any problems that might arise in relation to their phones.

Participants will receive the initial welcome text message within 10-14 days following the Initiation & Baseline visit. If a participant has not yet started taking their endocrine therapy medication at the time of the Initiation & Baseline visit, the initial welcome text message will be sent out within 10-14 days following confirmation of ET start date by the site study coordinators.

Once daily text message reminders have commenced, a text message will be sent to the participant's smart phone reminding her to take her medication every day at a time specified by the participant during enrollment (or a 10AM local time default if not specified by the participant).

The charges for the OSU-provided smart phones' unlimited talk, text, and data service plans will be paid by the grant through the first 12 months of the study, while active interventions are administered. At no time, will the study cover or pay for any apps or additional services, such as subscriptions, downloads or media that have associated charges. All charges outside of the phone service and data plan must be linked to a personal account and/or card. After that time, the participants will need to pay for the monthly fees themselves, if continued service is desired. The actual phones, however, will belong to patients at the end of 12 months. Alternative contact information for someone who will know how to reach the participant will be collected on the iPhone Recipient Evaluation survey (Appendix XVIII), if possible. This may help reduce the number of participants lost to follow-up for the 18- & 24-month assessments.

7.3.3 TMR Intervention Standardization

The rigor and reproducibility of the TMR component of the intervention will be ensured through standardized text message reminders that are programmed and automated within the OSU Redcap platform. Any changes in reframing or rewording the messages during the course of the study, to ensure resonance over time, will be overseen by Dr. Naughton, implemented across all participants, and designed to maintain the positive framing and spirit of encouragement used in the initial text message library. Ongoing quality control will be managed by the OSU team and will include monitoring of the text message schedule and content, participants' response data, and any technical concerns or lapses in smart phone services.

7.4 Motivational Interviewing (MI) Counseling Component (Arms 2 and 3)

7.4.1 MI Counseling Intervention Content

Overview of MI Session Format:

The MI component of the study consists of a 5-session behavioral intervention utilizing motivational interviewing to target ET initiation, adherence, and persistence. These sessions will be conducted by a trained MI counselor, in a one-on-one format via telephone, and can be tailored to the participant's specific needs, circumstances and/or values. Participants will have the option to use a video call if that is their preference during the first and second counseling sessions.

MI sessions will begin as soon as possible after randomization but no earlier than the participant's ET has been initiated. Patients who do not complete the first MI session within 8 weeks of randomization will be withdrawn, although exceptions for extenuating circumstances, such as hospitalizations, may be granted at the discretion of the study PIs.

The first counseling session will provide a brief explanation of the program to participants, review the purpose of counseling, answer any questions regarding the purpose of the study or study procedures, as well as walk participants through the GET SET MI workbook, and how to use it. Study participants will be asked to complete 4 subsequent telephone-based counseling sessions over the course of 6-9 months following the initial counseling session.

Five total MI sessions will be conducted between randomization and 38 weeks post-randomization, with varied lengths of time between sessions. For patients who have not completed all five MI sessions by 38 weeks, but wish to finish their sessions, up to eight additional weeks will be allowed to complete delayed sessions (up to 46 weeks post-randomization total). MI participants will be contacted in advance of each session via text, email and/or telephone, according to their preference specified during the first MI session, to schedule a convenient time for each MI session.

Overview of MI Content:

In each MI session, the MI counselor will follow a standardized guide to help develop motivation and skills to obtain and adhere to ET, including sustained engagement in clinical care. See Section 7.4.2 for details on MI session topic schedule. The MI counselor will use Rogerian techniques, like reflective listening, to help patients feel understood, raise awareness of any ambivalence they feel and of any discrepancies between their values and their expected behaviors, leading them to make self-motivating statements to adhere [32, 41, 59, 60]. MI counselors use specific techniques to build clients' self-efficacy, such as helping them identify strategies to overcome barriers, conducting skills-building exercises, and leveraging facilitators, such as access to financial assistance programs, support from family or peers, and reminders.

The GET SET workbook is designed to aid study participants through the intervention, as well as facilitate conversation during counseling sessions and act as a journaling opportunity for participants in between counseling sessions. The workbook includes chapters on personal values, motivations, concerns, action planning, tips for medication adherence, and general resources that may be of interest to study participants. The workbook will be available to each participant in paper form, as well as electronic form through the GET SET study website.

Behavioral Support Intervention:

A behavioral intervention will be provided by trained counselors located at the coordinating center, at the University of North Carolina at Chapel Hill, overseen by Dr. Stephanie Wheeler.

- Participants will be paired with an individual counselor who will work with them throughout the 9-month counseling program. This assignment will only be changed in unusual circumstances, and at the discretion of the Multiple Principal Investigators (MPIs).
- The behavioral intervention is based on an adapted version of an integrative, poly-theoretical framework used previously in oral medication adherence research to identify factors that likely influence ET adherence.
- The behavioral intervention will ascertain how ready patients are to adhere to medical recommendations in terms of both motivation and self-efficacy, helps identify adherence barriers, and develop solutions to address numerous types of barriers.

Toolbox Format:

The individualized nature of MI requires some flexibility in how the intervention is delivered, and therefore, a toolbox approach will be used to meet the needs of each participant, optimizing medication adherence.

- Participants can actively choose from a toolbox of available activities, or counselors can suggest activities from the toolbox based on the participant's description of current needs
- Examples of activities include:
  - Values that Influence your Health Decisions
  - Getting Set with Endocrine Therapy
  - Planning Actions to Take
  - Dealing with Setbacks and Coping

Toolbox activities will also provide the ability to tailor the intervention to meet the needs of specific ethnic, socioeconomic and other specific populations.

7.4.2 Intervention Delivery

The Motivational Interviewing component of the GET SET trial will be delivered centrally by trained counselors from the University of North Carolina at Chapel Hill. The counseling sessions will only be delivered in English.

Participants will be paired with an individual MI counselor, who will work with the participant throughout the 9-month MI intervention. The counseling sessions will consist of a series of 5 semi-structured telephone calls, supplemented by a participant workbook, available in print and online. Online materials for the participants randomized into the MI counseling intervention will be accessible by a password-protected, secure website (www.getsetstudy.org). The generalized password will be printed in the workbook given to MI participants at the enrollment visit, after randomization. The website contains the following content: 1) general study information such as research objectives, eligibility criteria, study personnel contact information, 2) general information about endocrine therapy medication, 3) password-protected, arm-specific content such as patient information videos, CHART module access, and arm-specific study contact information (i.e. contact information for patients randomized to text message reminders will receive contact information for the Ohio State University coordinating center, and those patients randomized to counseling intervention will receive contact information for the UNC coordinating center), and 4) frequently asked questions relevant to the GET SET study. The website will be accessible via computer, tablet, or smart phone.

MI participants will be contacted in advance of each session via text, email and/or telephone, according to their preference specified during the first MI session, to schedule a convenient time for each MI session. A variety of days and times will be made available using a staff of MI counselors at the UNC Coordinating Center. To avoid MI counselor burnout and to provide back-up in case of absence, counseling hours will be divided among individual counselors. To ensure that counseling assignments over time are evenly distributed, assignment of new counseling participants will be alternated among counselors and total counseling time per week for each staff member will be monitored. In addition, counselors will be debriefed on their experience during weekly staff meetings, and counselors' notes will be audited regularly for fidelity and quality. During staff debriefings, the study team will discuss and troubleshoot challenges as they arise. Once a patient has been assigned to a particular counselor, this dyadic relationship will be maintained throughout the study except under unusual circumstances at the discretion of the MPIs.

Counseling sessions will last anywhere from 30-90 minutes (the initial calls may last longer), and they will be standardized as much as possible using a semi-structured guide. In each session, the MI counselor will follow a standardized guide to help develop motivation and skills for ET adherence, including encouraging sustained engagement in clinical care. These sessions provide: (1) choices of topics related to medication taking; (2) assessment of clients' motivation and self-efficacy; (3) values clarification exercises; (4) exploration of barriers and facilitators to address behavior; (5) elicitation of pros/cons of changing behavior; (6) goal-setting; and (7) standardized educational information that MI counselors can use to answer questions or probe on specific topics. The first hour-long session begins with building rapport and assessment of the patients' expectations for taking ET and invites them to choose from a menu of adherence topics that are salient to them. New topics may be selected at the follow-up phone sessions or the patient may choose to continue with a topic previously selected. The standardized MI guides range from rapport building and introducing ET concepts to assessment and finally goal setting.

A sample schedule for topics covered over the course of the 5-session intervention is listed below. This schedule is reflective of the GET SET pilot program. Progression of the MI sessions, based on the pilot data, moves from building rapport and assessing participants' expectations for taking ET to inviting the participant to choose from a toolbox of activities/topics that are applicable to current life experiences. This toolbox of activities will be made available at the beginning of each counseling session, as participants may have varying concerns or experiences over the course of the 9-month intervention. Additionally, at the beginning of each session, the counselor will review the activities completed previously and check in on any goals or plans for action that were made.

Sample MI Counseling Schedule:

Session #	Session Outline	Sample Activity with Chosen Topic
**1**	Welcome to GET SET<br>What is Important to You<br>What Motivates You<br>Identify Support Person<br>Select Topic<br>Make Plans	Topic: Worried about ET side effects<br>Problem/Barrier: Joint pain<br>Action to Overcome: Exercise and ask doctor for advice<br>Plan: Sign up for and attend at least 2 yoga classes a week.
**2**	Review Session 1<br>Check in on Session 1 Plans/Goals<br>Reflect on Important Values and Motivators<br>Select Topic<br>Make Plans and Set Goals<br>Dealing with Setbacks and Coping	Topic: Feeling stressed out<br>Problem/Barrier: Thinking about the cancer makes me stressed<br>Action to Overcome: Take more time for myself<br>Plan: Schedule at least 1 hour each week to do something by yourself (walk in park, get nails done, etc.)<br>Dealing with setbacks: Talk to my support person/people about my thoughts and struggles
**3,4,5**	Review Previous Session<br>Check in on Previous Plans/Goals<br>Select Topic<br>Make Plans and Set Goals	Topic: Have a hard time remembering to take my pills<br>Problem/Barrier: I travel a lot for work<br>Action to Overcome: Remember to pack pills<br>Plan: Add pills to my packing list for trips; keep "backup" pills in the bag I carry when I travel

7.4.3 Intervention Standardization

To maintain an appropriate balance between individual "tailoring" and testing a replicable intervention, we will incorporate the following strategies to standardize the delivery of the MI counseling sessions:

1. Centralized MI counselors: MI counselors will be housed at the UNC Coordinating Center under the direct supervision of the MPIs. The counselors will be experienced in the conduct of clinical research and will have prior experience working with cancer patients.
2. Standardized training of counselors: The counselors will attend three days of MI training that includes didactic sessions about the theoretical basis for, principles, techniques and spirit of health behavior change through MI as well as video demonstrations and practice sessions with role-play to develop and enhance MI skills. In addition to learning MI techniques, the training will provide detailed information about ET, cultural sensitivity and competency, barriers women taking ET often face, and potential practical solutions to these barriers.
3. Standardized counseling guides: MI counselors will follow counseling guides that include scripts and a toolbox of activities that can be used during each session. The standardized guides range from rapport building and introducing ET concepts to assessment and final goal setting. While counseling sessions will be modified to the individual needs of each participant, the toolbox of activities available will be standardized across all participants.
4. Standardized patient interaction and materials: All participants will undergo the same frequency of individual sessions according to set schedule. By holding the intensity of interactions constant, we will limit the variability of contact between the counselor and participants. Additionally, the standardized workbook will "fill in" gaps where individual sessions differ.
5. Standardized data entry: The counselors will use a standardized guide to conduct the MI sessions. During sessions, the counselors will use a standardized data entry form in the intervention administrative database to record topics chosen, goals set, and strategies developed. The process of writing these steps down serves, not only to record session content, but also prompts the counselor to carry out each step in the protocol.
6. Standardized supervision of counselors: adapted from the PACT study [31, 61], a supervisor or MPI will hold monthly feedback and troubleshooting sessions with the counselor team. Counselors will also meet regularly with each other to share problem-solving techniques, resources, and provide profession peer support.

7.5 Enhanced Usual Care (Arm 4)

Usual care consists of all the care related to breast cancer delivered to patients during non-clinical research encounters and by the providers not related to the research study team. While we expect the usual care activities to vary greatly across providers and clinics, we expect that routine activities include: physical examination, evaluation of symptoms related to breast cancer treatment, and individualized management of those symptoms. For most breast cancer survivors, usual care includes: clinic visits every 3-6 months per national guidelines, and additional interactions with their healthcare providers via phone or electronic means as well as urgent visits for medical problems.

In addition to this usual clinical care, participants in all arms will receive general health education information for breast cancer survivors, delivered via interactive modules on the study website, that they can access at any time. This health education will be delivered via the CHART tool. This combination is referred to in the protocol as Enhanced Usual Care.

CHART is an online health behavior assessment tool that aims to strengthen behavioral interventions and collect health behavior data by assessing, improving, modifying participants' behavioral risk and risk factors. This is achieved through online surveys in a variety of health behavior topics including physical activity, eating habits, emotional health, sleep habits, and medication adherence. Modules provided to study participants include: physical activity, eating habits, weight, emotional health, tobacco use, alcohol use, sleep habits and balance.

Study participants will be provided with a generic password to enter the interactive health education modules; however, the login and password will not identify patients and the CHART modules will not retain any patient inputs for research purposes.

The functions of the research study team are entirely distinct from those of the patient's medical providers in order to test a freestanding behavioral intervention that is not a replacement for usual clinical care.

7.6 Incentives

Participants will receive a Visa gift card worth $50 for completion of the baseline visit. Participants will then receive an additional Visa gift card worth $20 for completion of each of the 3, 6, 12, 18 and 24 month surveys. Finally, participants will receive a Visa gift card worth $55 for completion of the final Pillsy® Cap data collection at the 24 month time point. The most that a participant could receive for participation in this study is $205. If a participant does not complete the necessary requirements for the gift card based on the time point, they will not receive a gift card. Visa gift cards will be provided by the UNC coordinating center, and will be mailed directly to the patient upon verification of study requirement completion at each time point. Distribution of gift cards may take up to 12 weeks.

8.0 ADVERSE EVENTS

We do not anticipate any additional adverse events related to participation in this study beyond usual care.

If a participant reports feeling distressed as a result of study participation, either as a result of participating in the Motivational Interviewing (MT), interactive Text Messaging Reminders (TMR), or electronic patient reported outcome measures (ePRO), they may choose not to continue or to complete them and/or speak with the site staff. Participants experiencing any physical or psychological complications related to their endocrine therapy should discuss this with their treating physician.

9.0 MEASURES

**Domains and Measures***	**# items**	**Baseline**	**Months 3, 6, 18**	**Month 12**	**Month 24**
**Socio-demographics:**					
**Alliance Patient Questionnaire (select items):**<br>Age, gender, ethnicity, race, language, education, marital status, health insurance, household size, income, employment status	13<br>(9 items at Months 12, 24)	X		X<br>• Marital status<br>• Health insurance<br>• Household size/income<br>• Employment	X<br>• Marital status<br>• Health insurance<br>• Household size/income<br>• Employment
**Medical and Family History**:					
Medical and Family History	7	X		X	X
**Quality of Life/ Psychosocial:**					
PROMIS Global Health Questionnaire	10	X		X	X
Breast Cancer Prevention Trial (BCPT) Symptom Questionnaire	25	X		X	X
PROMIS Depression Short Form 8a	8	X		X	X

Concerns About Cancer Recurrence (CARS); overall concerns subscale only	4	X		X	X
Perceived Stress Scale	10	X		X	
Modified Medical Outcomes Study Social Support (mMOS-SS) Questionnaire	8	X		X	
**ET Medication Use:**					
Medication Understanding and Use Self-Efficacy Scale (MUSE)	8	X	X	X	X
Self-reported ET adherence and persistence	17	X	X	X	X
**Intervention Acceptability**:					
Intervention Evaluation: Acceptability, Appropriateness, Ease of Use	15			X	
Was It Worth It (WIWI) Questionnaire for Alliance clinical trial participation	5			X	
**TOTAL ITEMS:**	130	110	25	126	88
**TOTAL ESTIMATED TIME:**		37	10	42	30

9.1 Alliance Patient Questionnaire – Socio-demographics (Appendix II)

Women will be asked 13 items about their age, race, ethnicity, education, marital status, health insurance coverage, household size, income, and employment using selected items from section 8 of the Alliance Patient Questionnaire [62-64]. A shortened, 9-item survey will be administered at month 12 and month 24, including only items regarding age, education, marital status, health insurance coverage, household size, income, and employment.

9.2 Personal Health History (Appendix III)

Women will be asked 10-items about their personal health history and breast cancer characteristics. Domains assessed will include menopausal status prior to diagnosis, parity, and family history of breast cancer. The survey will be administered at baseline, and months 12 and 24.

9.3 PROMIS Global Health Questionnaire v1.2 (Appendix IV)

The PROMIS Global Health Questionnaire is a self-reported, 10-item instrument commonly used to assess an individual's general evaluation of their health within the five PROMIS domains (physical function, fatigue, pain, emotional distress, and social health). The global health questionnaire construct validity is supported by its correlation with other PROMIS multi-item scales [65]. Item responses range from 1 (meaning poor, not at all, always or very severe) to 5 (meaning excellent, completely, never, none). One item assessing average pain is measured on a scale 0-10, with 0 meaning "no pain" and 10 meaning "worst pain imaginable". The survey will be assessed at baseline, and month 12 and month 24.

9.4 Breast Cancer Prevention Trial (BCPT) Symptom Questionnaire (Appendix V)

The Breast Cancer Prevention Trial (BCPT) Symptom Questionnaire is a validated, 21-item self-report questionnaire used to assess commonly reported side effects associated with treatment and prevention of breast cancer [66, 67]. Items assess physical and psychological symptoms, such as

vaginal dryness, hot flashes, and joint pain, which are relevant to women undergoing treatment for breast cancer, over the last four weeks. Item responses range from 0 (meaning not at all) to 4 (meaning extremely). The survey will be administered at baseline, month 12, and month 24.

9.5 PROMIS Depression Short Form 8a v1.0 (Appendix VI)

The PROMIS Depression Short Form is a self-reported, 8-item instrument commonly used to measure general depression over the past seven days. The PROMIS depression item bank has demonstrated reliability, precision, and construct validity based on evidence of correlation with other instruments measuring depression, and the short-form demonstrated high correlation with the full item bank. The items focus on domains such as negative mood, decrease in positive affect, information-processing deficits, negative views of self, and negative social cognition [68]. Item responses range from 1 (meaning never) to 5 (meaning always). The survey will be administered at baseline, month 12, and month 24.

9.6 Concerns About Recurrence Scale (CARS) (Appendix VII)

The overall Fear of Recurrence subscale of the Concerns About Recurrence Scale (CARS) is a 4-item measure used to assess the extent of women's fears of breast cancer recurrence [69]. Testing of the scale provides evidence of good internal consistency, test-retest stability, and construct validity across different populations of women with breast cancer [69-71]. Additionally, the CARS is a breast cancer specific instrument. Item responses in the subscale (overall fear of recurrence) range from 1 (meaning "I don't think about it at all", "It does not upset me", "I never worry about it", or "Not at all afraid") to 6 (meaning "I think about it all the time", "It makes me very upset", "I worry about it all the time" or "Very afraid"). The survey will be administered at baseline, month 12, and month 24.

9.7 Perceived Stress Scale (PSS) (Appendix VIII)

The Perceived Stress Scale (PSS) is a self-reported, 10-item questionnaire used to measure general psychological stress. Initial testing of the PSS-14 (14 items) showed adequate internal and test-retest reliability and high correlation with self-reported health behaviors [72]. After further factor analysis, the scale was shortened to the 10-item scale. Testing of the PSS-10 provided evidence for high internal consistency and factorial validity [73]. Item response range from 0 (meaning never) to 4 (meaning very often). The survey will be administered at baseline and month 12.

9.8 Modified Medical Outcomes Study Social Support (mMOS-SS) Questionnaire (Appendix IX)

The Modified Medical Outcomes Study Social Support (mMOS-SS) Questionnaire is an 8-item instrument commonly used to assess various dimensions of social support. It is comprised of 8 items covering two domains (emotional and tangible support), each with four items. Psychometric testing of the 8-item instrument showed good internal reliability and good convergent, divergent and discriminate validity[74]. Item responses for the 8 items relating to aspects of social support range from 1 (meaning none of the time) to 5 (meaning all of the time). The survey will be administered at baseline and month 12.

9.9 Medical Use Self-Efficacy Scale (MUSE) (Appendix X)

The Medication Use Self-Efficacy Scale (MUSE) is an 8-item instrument measuring a person's understanding and ability to take prescription medication. Testing of the instrument has provided evidence that it is both valid and reliable [75]. Item responses range from 1 (meaning strongly disagree) to 4 (meaning strongly agree). The survey will be administered at baseline, and months 3, 6, 12, 18, and 24.

9.10 Self-reported Endocrine Therapy Adherence and Persistence (Appendix XI)

Women will be asked a series of previously piloted questions (17 items) about their endocrine therapy adherence and persistence. Domains assessed will include general adherence according to

prescription, specific self-reported adherence over the past 14 days, changes in endocrine therapy since initiation, potential reasons for non-adherence, self-efficacy of continuing with ET treatment plan, perceived risks due to non-adherence. The medication usage questionnaire was developed to understand both the extent of ET use and reason-specific underuse, based upon reviews of the literature[76-79] and input from clinical and health behavior experts. This questionnaire includes a multi-item measure of self-reported ET usage behavior as well as questions about recurrence risk perception, shared decision making, and overall assessment of ET. After initial development, we presented the questionnaire with a clinic-based sample of breast-cancer patients, conducted cognitive interviews, and refined the questionnaire based upon patient-reported response patterns and feedback regarding usability and content, as well as oncologist input on clinical relevance. The survey will be administered at baseline, and months 3, 6, 12, 18, and 24.

9.10.1 Tracking Adherence Through Pillsy Cap

Pillsy cap data collection will occur in real time through the Pillsy app via Bluetooth on the participant's smartphone. Each time the cap is removed from the prescription drug bottle, the date and time stamp will be communicated to the Pillsy app. Pillsy data collection will be monitored by the central project manager at UNC on a weekly basis using data reports run by the Pillsy database to ensure that cap data collection is occurring according to protocol. When necessary, the central project manager will contact the participant directly to help troubleshoot. If the central project manager and participant cannot resolve the issue via telephone. Pillsy cap data can be collected at a participant's routine clinical encounter to ensure maximum data retention. Routine clinical visits for breast cancer survivors in years 1-2 post-diagnosis are expected to occur every 3 months on average and no less frequently than every 6 months per national guidelines. Participants will only be asked to bring their Pillsy Cap to a routine clinical encounter to allow for a manual data collection to occur if absolutely necessary. If Pillsy cap data collection is necessary in the clinical setting but a participant does not anticipate returning to clinic during the data collection window (-7 or +90 days of the time-point), the participant will be asked to bring their cap to the center for data download. If returning to the center poses a hardship, they may return the cap by prepaid mail, with return/exchange of a new cap by mail.

9.11 Intervention Evaluation: Acceptability, Patient Satisfaction, and Appropriateness (Appendix XII)

Women will be asked a series of 15 items focused on evaluation of the GET SET intervention. These questions include the 4-item Acceptability of Intervention Measure (AIM) and 4-item Intervention Appropriateness Measure (IAM), which are psychometrically tested implementation outcomes measures[80], as well as 8-items regarding the ease of use and willingness to recommend the GET SET intervention. Testing of the AIM and IAM measures showed each of those measures to be valid and reliable measures of the targeted implementation outcomes. The remaining 8-items will focus on ease of use of certain components and willingness to recommend the GET SET intervention. While these items have not been psychometrically tested, they are more specific to our intervention components. The survey will be administered once at month 12.

9.12 Was It Worth It (WIWI) Questionnaire for Alliance Clinical Trial Participation (Appendix XIII)

The Was It Worth It (WIWI) Questionnaire is a 5-item survey measuring patient satisfaction with overall clinical trial participation. The survey will be administered once at month 12.

10.0 END OF TREATMENT/INTERVENTION

10.1 Duration of Protocol Intervention

Protocol intervention is to continue for 9 months. Please see the study calendar (Section 5.0) and the intervention section (Section 7.0) for intervention and following up time periods.

Completion of protocol intervention activities will be communicated to site staff by the central study staff via a monthly report including all participants who completed intervention activities in the previous 30 days and the corresponding completion date. Reports will only include participants who have completed intervention activities per protocol and will exclude withdrawals prior to the completion of intervention.

10.2 Criteria for Discontinuation of Protocol Intervention

In the absence of intervention delays due to adverse event(s), study participation may continue until one of the following criteria applies:

- Disease progression (e.g., progression to metastatic disease, a locoregional recurrence, or a primary breast cancer)
- Intercurrent illness that prevents further administration of treatment
- Patient decides to withdraw from the study
- General or specific changes in the patient's condition that render the patient unacceptable for further treatment in the judgment of the investigator
- Patient non-compliance with protocol specified activities (See Section 10.3.2)
- Termination of the study by sponsor

The reason(s) for protocol therapy discontinuation, the reason(s) for study removal, and the corresponding dates must be documented in the Case Report Form (CRF).

10.3 Follow-up

10.3.1 Duration of Follow-up

Adherence data will be collected continuously using the Pillsy Smart Pill Cap for a total of 24 months after randomization. All participants will receive reminders at regular intervals to sync the Pillsy cap with the smartphone. An automated text will be sent to all participants at Months 3, 9, 15 and 21 to provide the reminder. The automated text will read:

"*It's time to check that your GET SET Pillsy cap is working. Please turn your Bluetooth on in your phone settings, tap your Pillsy app, then place your pill bottle next to your phone. Make sure you leave the Pillsy app on. Please click <here> to confirm that it worked.*"

Participants will indicate whether the cap syncing process was successful by indicating "yes" or "no" on the hyperlink provided in the text. If a participant indicates "no", central study staff will be notified to contact the participant directly for troubleshooting. A subsequent text will be sent on the same day within a few hours of the first text. It will read:

"Please remember to use your Pillsy cap on any day that you take your medication. Thanks for being a part of GET SET."

If at any point, it becomes apparent that a participant's smart pill cap is not collecting data in real time or a participant has indicated the cap syncing process was not successful, the central project manager will contact the participant directly, if required, to troubleshoot any possible technical issues. If the central project manager and the participant cannot resolve the issue, the Pillsy cap data can be collected at the next routine clinical encounter at the site. Site staff would be responsible for collecting cap data at this time. Site staff will only be asked to collect cap data at a clinical encounter if necessary.

Batteries are guaranteed by the Pillsy Company for 6 months, and therefore, participants will be asked to change the battery proactively at 6 month intervals to avoid loss of data. The central project manager will mail a new battery and instruction sheet to each actively enrolled participant at months 6, 12, and 18 after the study initiation visit. The instruction sheet will provide detailed instructions on how to change the battery in the Pillsy cap, a reminder to sync

the participant's cap with her smart phone, and contact information for the central project manager in the event a participant needs to troubleshoot changing the battery or syncing her cap. In addition to the reminder text messages sent to participants at Months 3, 9, 15 and 21, participants will receive a reminder to sync the Pillsy cap every three months for the duration of the study participation.

Patient reported outcomes data will be collected via surveys using the ePRO app. In addition to reminder notification through the ePRO app, all participants will receive an automated reminder text regarding their upcoming follow-up assessments at M3, 6, 12, 18, and 24. The automated text will read: "*It's time to complete a short survey for GET SET. Please open the Patient Cloud application on your smart phone, and complete all forms available. Don't forget to click submit when you are finished. Thank you for being a part of the study!*" Participants will complete a total of 5 follow-up assessments at varying time points during the 24 months post randomization. These assessments occur at Months 3, 6, 12, 18, and 24. See PRO assessment timeline below:

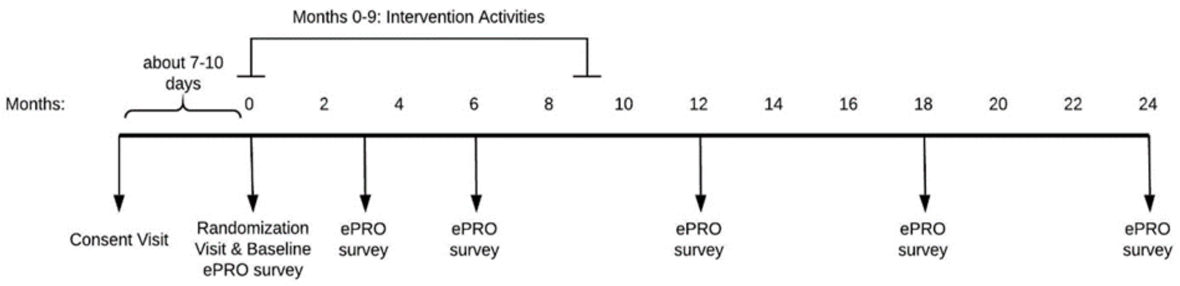

Participants randomized to the TMR component of the intervention (Arms 1 &3) will receive a follow-up process evaluation survey texted to them at the end of the 9-month intervention period. The texted survey will be sent directly to the participant at the end of the final monthly questions. See Section 11.6.3 for more information and Appendix XVII for the questions included.

Participants who receive a study-provided smartphone will receive an evaluation survey at the end of the 12-month contract period. The texted survey will be sent directly to the participant. See Section 11.6.3 for more information and Appendix XVIII for the questions included. Additionally, participants who receive a study-provided smartphone, regardless of randomization, may be contacted by study staff at various times during the 12 months of provided phone service. These follow-up texts or phone calls may involve topics such as troubleshooting and repair of provided phones, as well as information about service options after the free service contract period ends.

10.3.2 Follow-up for Patients who stop ET medication during study participation

For participants who stop ET before starting intervention activities and do not intend to resume taking the medication:

- Participants will be withdrawn from the study, and follow-up for all protocol endpoints will be discontinued.

For participants who stop ET after starting intervention activities, but do not have disease progression:

- If willing to continue receiving study intervention activities, patients should be instructed to store the Pillsy cap in a safe place, and follow-up will continue for protocol endpoints as required by the Study Calendar. During study follow-up if the

patient resumes taking medication, they should resume using the Pillsy Smart Pill Cap.

- If unwilling to continue receiving study intervention activities, please see Section 10.3.3.

For participants who stop ET after starting intervention activities due to disease progression

- These patients should be instructed to discontinue using the Pillsy cap at time of disease progression and will discontinue all assigned protocol interventions (see Section 10.2). End of treatment procedures should be followed for these patients. Patients with disease progression will be considered evaluable for all endpoints occurring prior to their disease progression.

For participants who stop ET after starting intervention activities due to disease free survival event (DFS event). A DFS event is defined as one of the following: invasive ipsilateral recurrence, local/regional invasive recurrence, distant recurrence, death from any cause, invasive contralateral breast cancer, ipsilateral or contralateral DCIS, or second primary invasive non-breast cancer.

- Patients who experience a DFS event during study intervention (i.e., prior to completion of counseling and/or text intervention), will discontinue protocol intervention (see section 10.2). To ensure timely discontinuation of intervention activities and minimize patient distress, the coordinator should notify central study staff of disease progression by email as soon as possible, in additions to completing the appropriate Case Report Form (CRF).
- Patients who experience a DFS event prior to end of study follow-up (24 months) should discontinue use of Pillsy cap at time of disease progression. Collection of other follow-up data including ePRO surveys will stop at the time of the DFS event, and the patient will be evaluable for all timepoints prior to DFS event. The coordinator should document disease progression as the reason(s) for protocol therapy discontinuation, and the corresponding dates must be documented in the CRF. Documentation of the DFS event should be uploaded per protocol (see Section 6.1).

10.3.3 Follow-up for Patients who Stop Study Intervention Early

For participants who stop protocol intervention activities, for any reason other than disease progression and:

- Are *willing* to continue being followed for both medication adherence outcomes (i.e. continue using the Pillsy Smart Pill Cap whenever they take medication), and for patient-reported outcomes (PRO) (i.e. continue completing ePRO surveys):

  Follow-up will continue for these protocol endpoints as required by the Study Calendar.

- Are *willing* to continue being followed for the medication adherence outcomes (i.e. continue using the Pillsy Smart Pill Cap whenever they take medication), but *unwilling* to continue being followed for PROs (i.e. continue completing the ePRO surveys):

  Follow-up will continue for the medication adherence endpoint as required by the Study Calendar.

- Are *unwilling* to continue being followed for medication adherence outcomes (i.e. continue using the Pillsy Smart Pill Cap whenever they take medication), but are *willing* to continue being followed for PROs (i.e. continue completing the ePRO surveys):

Participants will be withdrawn from the study, and follow-up for all protocol endpoints will be discontinued.

- Are *unwilling* to continue being followed for medication adherence outcomes (i.e. continue using the Pillsy Smart Pill Cap whenever they take medication), and for PROs (i.e. continue completing the ePRO surveys):

  Participants will be withdrawn from the study, and follow-up for all protocol endpoints will be discontinued.

10.4 Extraordinary Medical Circumstances

If, at any time the constraints of this protocol are detrimental to the patient's health and/or the patient no longer wishes to continue, study participation shall be discontinued. In this event:

- Document the reason(s) for discontinuation of therapy on data forms.
- Follow the patient for protocol endpoints as required by the Study Calendar.

10.5 Managing ineligible patients and registered patients who never receive protocol intervention

Definition of ineligible patient

A study participant who is registered to the trial but does not meet all of the eligibility criteria is deemed to be ineligible.

Follow-up for ineligible patients who continue with protocol intervention

Patients who are deemed ineligible after registering may continue protocol intervention, provided the treating physician, study chair, and executive officer agree there are no safety concerns if the patient continues protocol intervention. All scans, tests, and data submission are to continue as if the patient were eligible. Notification of the local IRB may be necessary per local IRB policies.

Follow-up for ineligible patients who discontinue protocol intervention

For patients who are deemed ineligible after registering to the trial, who start intervention, but then discontinue study intervention, the same data submission requirements are to be followed as for those patients who are eligible and who discontinue study intervention.

10.6 Follow-up for patients who are registered, but who never start study intervention

For all study participants who are registered to the trial but who never receive study intervention (regardless of eligibility), the follow-up requirements are specified below.

Non-treatment DCP studies: Baseline and off-treatment notice data submission required. See the Data Submission Schedule accompanying the All Forms Packet.

11.0 STATISTICAL CONSIDERATIONS

11.1 Study Design

This is a multiple-armed randomized, controlled clinical trial to determine the ability of two single component interventions (TMR; MI) to improve ET adherence among HR+ breast cancer survivors when compared with enhanced usual care. Additionally, this randomized study is designed to determine the ability of the multicomponent intervention (TMR+MI) to improve ET adherence among HR+ breast cancer survivors when compared with enhanced usual care if either or both single component interventions are deemed effective at improving ET adherence. Women will be randomized in a 1:1:1:1 ratio to receive either enhanced usual care, TMR-only, MI-only, or TMR+MI. Randomization will be stratified on the basis of age, in years, (< 50; ≥ 50) and race (Black women; non-Black women). These two stratification factors were chosen because they are clinically meaningful prognostic factors assumed to have a powerful effect on the study outcomes.

11.1.1 Rationale

We considered multiple study design options for this clinical trial to assess the statistical and feasibility tradeoffs with each one, including a 3-arm head-to-head trial (enhanced usual care; TMR-only; TMR+MI) as well as a 2-by-2 factorial design powered to detect a reasonable target interaction effect. We concluded that these other design options were inferior to the current design from an efficiency and sample size practicality perspective. Further, we determined that the current study design was most appropriate in allowing assessment of each individual intervention's effectiveness versus enhanced usual care, while also permitting some exploration of potential synergy and the quantification of the marginal gains of TMR+MI over either single component alone as well as the imprecision of such potential gains. Although the current study design is underpowered to formally assess the interaction between TMR and MI needed in a pre-planned statistical analysis, the information gained in an explorative manner could motivate future studies of multicomponent interventions, that is, if either or both single component interventions are deemed effective at improving ET adherence. Further, although conceivably a multicomponent intervention consisting of both MI and TMR may better target complex barriers and reinforce motivation and self-efficacy through complementary formats, this potential gain in effectiveness will likely come at the expense of additional resources, more complexity in delivery, and potentially lower feasibility for broad dissemination. The current study design will, therefore, also allow us to assess and estimate the cost trade-offs associated with the single component interventions and the multicomponent intervention.

11.2 Description of Randomization Routine

After a woman is registered she will be assigned to one of the four arms (enhanced usual care; TMR; MI; TMR + MI) with an equal allocation ratio utilizing a dynamic allocation algorithm based on the methods by Pocock and Simon. The goal of the algorithm is to maintain arm balance with respect to the aforementioned stratification factors. In order to ensure that arm assignment is not deterministic, a level of randomness will be added to the algorithm such that patients will be assigned to the arm that leads to more imbalance 10% of the time. Dynamic allocation is a common approach in cancer clinical trials where we desire arms to be balanced across several prognostic factors. The method is considered robust because it ensures excellent arm balance overall and within the stratification factor levels, even with a large number of stratification factors.

11.3 Sample Size, Accrual Time, and Study Duration

The co-primary aims are to separately demonstrate that TMR-only and MI-only will result in higher adherence at 12 months, compared with enhanced usual care. Enhanced usual care adherence at 12 months is assumed to be 69%, based on our preliminary studies of pharmacy refill data from claims in Medicare, Medicaid, and private insurance populations, using weighted estimates of 12-month adherence among black women < 50 years old, non-black women < 50 years old, black women ≥ 50 years old, and non-black women ≥ 50 years old. We hypothesize 81% adherence for the TMR-only and MI-only intervention arms based on preliminary data from our GETSET MI counseling pilot intervention. We desire a primary family-wise two-sided Type I error rate of $\alpha = 0.05$; under a Bonferroni correction, each test will be conducted with nominal $\alpha = 0.05 / 2 = 0.025$. At a 2.5% two-sided significance level, the study will have at least 80% power to detect differences in adherence (81% in single component intervention arms vs 69% in enhanced usual care) if 263 evaluable patients are randomized each to the TMR-only, MI-only, and enhanced usual care arms. The test statistic used in this calculation is the two-sided Z-Test with continuity correction and pooled variance. Evaluable patients (per-protocol set) are defined as all women who signed a consent form, met the eligibility criteria, were randomized, and completed at least 12 months of study follow-up; in other words, the power calculation was based on the per-protocol set. To achieve an equal allocation of evaluable patients across the 4 arms, 263 patients will also be randomized to the TMR+MI arm for a total of no less than 1052 evaluable patients randomized.

Because we anticipate ineligible and loss-to-follow-up of approximately 11%, consistent with other clinical trials of cancer survivors, we will over-recruit to allow for 1052 evaluable patient records to be analyzed on the per-protocol set, rendering a recruitment target of 1180 randomized patients total (295 per arm). Further, due to the study's focus on minorities and young patients, the study will be conducted at Alliance NCORP sites that serve a high proportion of black women and women aged < 50 years old but also care for patients from other strata. Therefore, the study's targeted accrual goal is to enroll approximately 30% black women and 30% women aged < 50 years old. Details on how we will achieve this important accrual goal are found in Section 11.9.

We anticipate an accrual rate of 33 patients per month; therefore, to achieve the targeted accrual of 1180 randomized patients we anticipate that the accrual period will be approximately 36 months. Since scheduled follow-up ends at 24 months post-randomization, the corresponding total study duration will be approximately 60 months.

11.4 Statement for Primary Endpoint

11.4.1 Primary Endpoint

The primary endpoint is ET adherence at 12 months post-randomization as measured by electronic pill monitoring cap (Pillsy-reported) and defined as the proportion of days covered by any ET medication at or above 80% (i.e. taking ET at least 292 of 365 days).

A patient will have an assessment of "adherent" or "non-adherent" for each 24-hour period following the first cap open (or close) event after the Pillsy cap activation (i.e., if the cap is activated at 4pm, the patient's adherence measurement begins the following calendar day at 3am, whether or not any events are registered before that time). The Pillsy cap activation is defined as the first open and close on the same day. Adherent measurements will begin from the first open (or close) event recorded on a calendar date after the cap activation date. The definition of a calendar day and an event of "adherent" (or "non-adherent") are listed below.

- A day begins at 3am on the calendar date, in the patient's local time zone (which is set up at the beginning of study)
- the first "open" or "close" event registered by the cap after 3am indicates an adherent day for that calendar day
- additional "open" or "close" events occurring between 3am and 2:59am on the following calendar day, are disregarded in the adherence measurements.
- if no "open" or "close" event occurs between 3am on a given calendar day and 2:59am on the following calendar day, that day is counted as non-adherent.

11.4.2 Analysis Plan for Primary Endpoint

The primary analysis will be based on a modified intention-to-treat population (mITT; our full analysis set), defined as all women who signed a consent form, met the eligibility criteria, were randomized, took ET on at least one occasion, and began the intervention, if randomized to an active intervention arm; furthermore, these women will be analyzed in the arms to which they were randomized. Excluding women who do not meet the eligibility criteria or do not take ET on at least one occasion is not expected to introduce bias. Within the mITT analysis population, women who do not take ET for at least 292 days will be deemed non-adherent at 12 months. For the primary analysis, the chi-squared test for differences in the proportion adherent in each single component intervention arm compared with the enhanced usual care arm will be applied. For each comparison (there are two), statistical significance will be assessed at the 2.5% level.

Should either TMR-only or MI-only arms achieve statistical significance at its allocated α-level (or accumulated unused α-levels if both achieve statistical significance), then the TMR+MI vs enhanced usual care comparison will be assessed and the unused α-level passed down and applied to determine statistical significance. In this way, the Type I error rate applied to the

primary family of endpoints can be controlled at the 5% level. Assuming 85% ET adherence at 12 months post-randomization in the TMR+MI arm, the study will be adequately powered to detect an absolute difference of 16% vs enhanced usual care.

11.4.3 Interim and Final Analysis for Primary Endpoint

No interim analyses for futility or efficacy are planned. The final analysis for the primary endpoint will be performed once all randomized patients have had the opportunity to complete 12 months of ET.

11.4.4 Planned Sensitivity Analyses for Primary Endpoint

We are also interested in evaluating whether the intervention effects on ET adherence differ by the stratification factors age (< 50; ≥ 50) and race (Black women; non-Black women). Therefore, logistic regression is planned to model the binary measure of adherence at 12 months separately for each intervention vs enhanced usual care comparison so that three separate logistic regression models will be estimated. Covariates included in each logistic regression model will be: age (< 50; ≥ 50), race (Black women; non-Black women), intervention arm assignment, and the interaction terms between each stratification factor (age and race) and intervention arm assignment to ascertain whether the magnitude of the intervention effect is different across the levels of age and race. Statistical significance of the interaction terms will be assessed at the 10% level with the likelihood ratio test comparing models with and without the interaction terms. We will report odds ratios and their associated 95% confidence intervals.

As an additional, albeit exploratory analysis, we will fit a single logistic regression model to model ET adherence at 12 months that includes main effects for TMR and MI, and an interaction between TMR and MI. The goal of this analysis is to consider both the separate effects of each intervention and the benefits of receiving both interventions together. Because the study was not prospectively powered to detect such an interaction effect, assessing the possibility of synergism is explorative. To allow for interpretation of the magnitude of any synergism between the interventions, as well as an indication of the imprecision of the results for the interaction, the regression coefficient and 95% confidence interval for the interaction term will be reported.

The mITT analysis set will tend to give a conservative estimate for each intervention vs enhanced usual care effect, because of including in the analysis women who have not conformed entirely with the protocol. Therefore, the primary analysis will also be performed on the per-protocol set defined as all women in the mITT analysis population who completed at least 12 months of study follow-up post-randomization. Additionally, the sensitivity analyses described above will be repeated on the per-protocol set.

11.5 Statement for Secondary Endpoints

11.5.1 Secondary Endpoints

There is a single secondary endpoint that may provide supportive information about the longer-term effect of the single and multi-component interventions compared with enhanced usual care.

1. Pillsy-reported adherence at 24 months post-randomization and defined as the proportion of days covered by any ET medication at or above 80% (i.e. taking ET at least 584 or 730 days).

11.5.2 Analysis Plan for Secondary Endpoints

We hypothesize that each single component intervention and the multicomponent intervention will result in a higher proportion of Pillsy-reported adherence at 24 months compared with enhanced usual care. The three planned statistical tests corresponding to each pairwise comparison constitute a secondary family of tests. To maintain the study-wise Type I error rate

at 5% across the primary and secondary family of tests, any unused α-level from testing the primary family will be passed down, such that the secondary family may be examined and may contribute important supportive information about the longer-term effectiveness of the interventions. Provided that some unused α remains, the secondary family of tests will be conducted and will be tested with the Holm procedure. The primary analyses described above in Section 13.4.2 for the primary family will be applied to the secondary family.

11.5.3 Planned Sensitivity Analyses for Secondary Endpoints

The planned sensitivity analyses for PILLSY-reported adherence at 12 months and described in Section 13.4.4 will be performed for PILLSY-reported adherence at 24 months. Further, because PILLSY-reported adherence is captured at 12 and 24 months for each patient, we will assess changes in adherence within each of the 4 arms. Specifically, to assess changes in adherence within each of the 4 arms, we will test for marginal homogeneity using McNemar tests for comparing dependent proportions; estimated proportions adherent at 12 and 24 months along with the corresponding 95% confidence interval for the difference will be provided.

11.6 Statement for Exploratory Endpoints

11.6.1 Exploratory Endpoints

There are several exploratory endpoints that will be evaluated in an explorative and descriptive fashion to contextualize and to further aid our understanding of the potential effects of the interventions on medication self-efficacy, health-related quality of life, and cancer worry. Additionally, there is an exploratory endpoint related to cost associated with the TMR-only, MI-only, and TMR+MI interventions, relative to enhanced usual care. The accrual pattern of Black women and younger women will also be evaluated separately at the initial 50% of patients enrolled and at the end of the trial. To evaluate the accrual pattern at the initial 50% of patients enrolled could inform the design and conduct of future Alliance studies where researchers may wish to over-sample racial and ethnic sub-groups, and strengthen support for sites to recruit Black participants during the latter half of enrollment if it is needed. Exploratory endpoints are intended to be hypothesis-generating and not definitive and are summarized here:

1. The serially measured self-reported adherence at 3, 6, 12, 18, and 24 months and defined as the proportion of days covered by any ET medication at or above 80% at each assessment month.
2. The serially measured patient-reported MUSE score at baseline, and at months 3, 6, 12, 18, and 24.
3. The serially measured physical health subscale obtained from the PROMIS Global Health Questionnaire at baseline, and at months 12 and 24.
4. The serially measured mental health subscale obtained from the PROMIS Global Health Questionnaire at baseline, and at months 12 and 24.
5. The serially measured patient-reported total score obtained from the PROMIS Depression Short Form 8a at baseline, and at 12 and 24 months.
6. The serially measured patient-reported overall fear of recurrence subscale from the Concerns of Recurrence Scale at baseline, and at months 12 and 24.
7. The serially measured patient-reported total score obtained from the Perceived Stress Scale at baseline, and at month 12.
8. The serially measured patient-reported total score obtained from the Medical Outcomes Study (MOS) Social Support Questionnaire at baseline, and at month 12.
9. Disease-free survival including DCIS.

10. The total incremental per-patient cost over the first 12 months.
11. Patient-reported intervention acceptability at month 12.
12. Patient-reported research study satisfaction at month 12.
13. The accrual pattern on Black women and younger women at the initial 50% of patients enrolled (590 patients) and after the trial is closed.

11.6.2 Analysis Plan for Exploratory Endpoints

Findings regarding exploratory endpoints will be considered hypothesis-generating and will require confirmation in further independent study.

Self-reported adherence is recorded at 3, 6, 12, 18 and 24 months. A longitudinal analysis, therefore, will be used for the binary response; marginal probability of adherence will be modeled as a logistic function of covariates and the within-patient association among the repeated binary responses will assume to follow an unstructured pairwise log odds ratio pattern. The goal of the longitudinal analysis is to determine whether patterns of change in self-reported adherence are the same for the 4 arms.

The remaining patient-reported exploratory endpoints measuring medication self-efficacy, aspects of health-related quality of life, and cancer worry, and are continuous in nature and measured longitudinally over the 24-month study period. These patient-reported outcomes will be summarized descriptively over the study period by all 4 randomization arms. For each pairwise intervention vs enhanced usual care comparisons, longitudinal modeling of the continuous patient reported outcome measures over time will be separately performed using linear mixed models. The baseline values will be retained as part of the response vector. Furthermore, we will assume that the mean values in each 2-level arm (e.g. TMR-only; enhanced usual care) are equal at baseline. Stated differently, each longitudinal model will include indicators for time (baseline being the reference level for time) and the interaction between the 2-level arm and time, but no main effect of arm. The focus in these analyses will be on the interaction effect, which concerns the intervention vs enhanced usual care comparisons of the patterns of changes in the mean values. In addition to plotting the model-based mean response profiles by the 2-levels of arm, we will report 12- and 24-month time-specific comparisons between the intervention and enhanced usual care arms. Because arm assignment is random, constraining the randomization arm means at baseline to be equal will, in general, be more powerful than including the arm main effect and making no assumptions about arm differences in the mean response at baseline. Also this modeling approach for adjusting for baseline incorporates all available data at baseline, and at 12- and 24-months in the analysis, despite the fact that some patients may be missing baseline values. This would not be the case if we analyzed the post-baseline values and made an adjustment for the baseline value by including it as a covariate. Other covariates considered for inclusion in each longitudinal model include the stratification factors age and race; the two-way interaction terms between each stratification factor and the indicator for arm (e.g. TMR-only; enhanced usual care) as well as the three-way interaction terms between each stratification factor, arm, and time.

Disease-free survival (DFS) including DCIS (DFS-DCIS) is defined as the length of time from the date of random assignment to any recurrent disease (including ipsilateral breast recurrence), the appearance of a second primary cancer (including contralateral breast cancer), ipsilateral or contralateral DCIS, or death, whichever occurs first. DFS-DCIS percentages, standard errors, and intervention effect comparisons (each intervention vs. enhanced usual care) will be obtained from the Kaplan-Meier method, Greenwood's formula, and log-rank tests, respectively. Cox proportional hazards regression models will be used to estimate relative risks and 95% confidence intervals for the pairwise intervention comparisons.

We are also interested in estimating the total cost (sum of fixed and variable costs) in current US dollars per person recruited to the TMR-only, MI-only, or TMR+MI arms, relative to enhanced usual care. We will document all fixed and variable costs associated with delivering the interventions (above and beyond usual care) from the health system perspective, including personnel costs associated with identifying eligible patients, MI counseling, TMR message delivery system setup and monitoring, and educational materials printing/mailing costs. For MI, allowable costs will include all counselor time required to schedule, complete, and document MI sessions. For TMR, allowable costs will include staff time to support the text-messaging platform, provision of phone service for 12 months to participants without text-messaging-capable phones, and staff time to troubleshoot participant problems with phones or other technical aspects of the TMR intervention. Patient time required to complete surveys and research coordinator time for study-related procedures will not be included.

We will report total incremental costs for the TMR-only, MI-only, and TMR+MI intervention arms relative to enhanced usual care. Should any of the active intervention arms be efficacious compared to enhanced usual care, we will compute incremental cost-effectiveness ratios reporting the cost per 1 percentage point gain in PDC, compared to enhanced usual care. We will also perform additional analyses to examine the effect of different pricing of intervention delivery (potentially negotiable for a larger scaled program). For analyses from the societal perspective, we will estimate the time spent participating in the intervention, and use the human capital method to calculate the value of patient time/opportunity cost of each intervention, relative to usual care.

At 12 months, the following measures will be administered to assess intervention acceptability from the patients' perspective: (1) the 4-item Acceptability of Intervention Measure[80]; (2) the 4-item Intervention Appropriateness Measure[80]; (3) a series of 7 ad hoc items specific to GET SET intervention focused on ease of use of certain components and willingness to recommend; and (4) Was It Worth It (WIWI) Questionnaire. Each response item on these three surveys will be descriptively summarized according to arm and tabulated.

Once accrual reaches 50% of total accrual (590 participants, regardless of eligibility), we will perform the following data analyses (A-C):

(A) To describe the trajectory of accrual per month in A191901, the overall accrual rate will be plotted longitudinally (actual vs. expected accrual).

(B) To describe accrual patterns in A191901 by site characteristics, descriptive statistics will be tabulated by race, and by age. The variables will include parent site type (Lead Academic Site; Other Main Site; NCORP site; Minority NCORP site), geographic region (Northeast, North Central, South and West by the National longitudinal study of youth), neighborhood racial composition in quartiles (4 categories, by race strata only), and site volume of Alliance accruals (4 categories, by race strata only). The neighborhood racial composition is categorized in quartile of proportion of Black residents in the zip code in which accruing site or sub-site (designated by CTEP code) is located according to 2020 US Census data (provided by the University of North Carolina's Cancer Informatics and Population Health Resource). The site volume will be categorized in quartiles based on Alliance accrual records of # patients enrolled at each site (designated by CTEP code) to Alliance interventional studies during 2018-19 (low, low-mid, high-mid, and high) among those Alliance sites who have enrolled at least 1 participant to A191901. Our hypothesis is that a lower total number of patients accrued to interventional Alliance trials in 2018-19 ("low site volume") will be associated with a higher proportion of Black patients enrolled by the site to this trial (A191901).

(C) The descriptive statistics on site volume will be tabulated by race and overall (Table 1) Spearman's Rho correlations coefficient will be applied to evaluate the association between the proportion of Black participant accruals in A191901 (beginning in December 2020) and the

number of patients enrolled by the site (classified by quartile) across all interventional Alliance trials in 2018-2019.

Note: Once the enrollment is completed, the above accrual analyses will be repeated. The Chi-square test will be applied to detect proportional difference of Black participants or of age < 50 years between each site characteristics. The detail analysis plan will be included in the SAP.

11.6.3 GET SET Process Evaluation

We will conduct a process evaluation to explore the integration of the GET SET intervention into the existing care system, taking into consideration the cost effectiveness. This will provide direction for scalability and future implementation, possibly allowing us to provide data to public health and policy decision makers about the facilitators and barriers that may accompany similar interventions in the real world.

Based on the process evaluation conducted in the counseling-based pilot study, we will use the Consolidated Framework for Implementation Research (CFIR)[81] and conduct semi-structured, one-on-one interviews with key informants from participating organizations, as well as intervention and research staff. No interviews will be conducted with participants. The qualitative interview guides will be tailored to include questions specific to each category of respondent, and will address constructs such as intervention characteristics, compatibility with patients' needs and resources, compatibility with organizational structure, and process. A semi-structured interview guide can be found in Appendix XVI.

Participant data from the "Intervention Evaluation: Acceptability, Appropriateness, Ease of Use" surveys collected at the end of the study will be analyzed. In addition, participants in the TMR component will also be asked to complete a brief survey about the ease of identifying and answering the text messages and responses, the length of time of receiving text message reminders, and other feedback about the TMR components (Appendix XVII). The survey will be texted directly to participants at the same time as their monthly questions (month 9).

Any participant randomized to the TMR component and who received an iPhone from OSU will be texted a brief evaluation survey (Appendix XVIII) about training provided, the ease of using the iPhone, benefits of having a smart phone, and whether they plan to purchase a phone/data plan at the end of the study-provided 12 month service contract with Verizon Wireless. The survey will be texted at the end of the 12 month service contract.

11.7 Missing Data

The amount of missing data in Alliance clinical trials, especially with respect to patient-reported outcomes (PROs), have been minimized, typically less than 5%, due to a long history of targeted approaches. In the event that the proportion of missing patient information is larger than 5%, we will examine the missingness mechanism by modeling indicators of missing values as a function of baseline patient characteristics, observed outcomes, and missingness of outcomes. Based on this diagnostic exercise, we will determine whether we can enhance the analyses by incorporating certain covariates or whether we need to explore more advanced methods.

11.8 Study Monitoring

11.8.1 Data and Safety Monitoring Board

This study will be monitored by the Alliance Data and Safety Monitoring Board (DSMB), an NCI-approved functioning body, twice per year. The DSMB follows the Alliance Policies and Procedures for all randomized phase III trials. The DSMB will review administrative information, accrual, including accrual of black women and according to age categories,

adverse events, and interim analysis results. All summary findings of the DSMB will be communicated to study investigators by the Alliance.

Required submission of patient demographic data for this study will be submitted automatically via OPEN.

11.8.2 Adverse Event Stopping Rule

No adverse events are being collected as part of this minimal risk, behavioral intervention study.

If a participant reports feeling distressed because of study participation, either because of participating in the TMR, MI, or electronic patient reported outcome measures, they may choose not to continue or to complete them and/or speak with the site staff. Participants experiencing any physical or psychological complications related to their endocrine therapy should discuss this with their treating physician.

11.9 Inclusion of Women and Minorities

This study will be available to all eligible patients, regardless of race or ethnic origin.

Young age and self-reported black race have been shown to be risk factors for low ET adherence. As such, we will make a concerted effort to accrue 30% (or 354 of 1180) of the total patients who self-identify as Black as well as 30% who are < 50 years old at recruitment. Additionally, the ideal case is for race and age to be uncorrelated with one another so that they have independent effects on the outcome of interest. Additionally, this will maximize power for the analyses described in Section 11.4.4. To achieve this, we will focus on accruing four non-overlapping groups (i.e., Black women < 50 years, non-Black women < 50 years, Black women ≥ 50 years, and non-Black women ≥ 50 years) and close accrual for each group independently. The target accrual numbers for each group are shown in the crosstable below:

	Black	Non-Black	Total
< 50	106	248	354
≥ 50	248	578	826
Total	354	826	1180

To achieve these accrual numbers, we have pursued advanced commitments from champions at minority-rich Alliance sites and referral centers seeing younger patients, namely, Gabrielle Rocque, MD (University of Alabama-Birmingham), Lisa Carey, MD (University of North Carolina), Ann Partridge, MD, MPH (Dana Farber Cancer Institute), and Heidi Klepin, MD, MS (Wake Forest University). The Health Disparities and Community Oncology Co-Chairs will assist in developing additional strategies to use in sites and are available for consultation, as needed.

We are not aware of any evidence suggesting differential effects in other subsets defined by race or ethnicity, and there is no reason to expect such differences to exist. Expected sizes of race by ethnicity subsets for patients randomized to this study are shown in the table below. In this study, all data will be tabulated according to race and ethnicity and reviewed; however, the sample size is not increased in order to provide additional power for subset analyses.

DOMESTIC PLANNED ENROLLMENT REPORT					
Racial Categories	Ethnic Categories				Total
	Not Hispanic or Latino		Hispanic or Latino		
	Female	Male	Female	Male	
American Indian/ Alaska Native	0	0	0	0	0
Asian	59	0	0	0	59
Native Hawaiian or Other Pacific Islander	0	0	0	0	0
Black or African American	285	0	69	0	354
White	604	0	163	0	767
More Than One Race	0	0	0	0	0
**Total**	948	0	232	0	1180

12.0 CREDENTIALING REQUIREMENTS

12.1 Institutional Credentialing

12.1.1 Protocol-Specific Training

The protocol-specific training (approximately 60 minutes long) will include a web-conference site activation training provided to the Site-Protocol PI and other registered site staff.

To schedule this training, the site staff should submit the following documents to the Central Project Manager at the UNC coordinating center:

(1) A completed Site Information Form, with the site-Protocol PI information as well as names and CTEP codes for all sites being activated.

(2) A completed Site Information Sheet, with all requested information regarding activated sites and contact information for all registered site staff, including the Site-Protocol PI.

Once submitted, the Central Study Project Manager will work with local site staff to schedule the site activation training. All site activation training materials, including a comprehensive set of training slides, site staff resource documents, and relevant study forms such as the Pillsy Request Form and Smart Phone request form, will be provided prior to the training along with the web-conference invitation. The site activation training will include the following information:

- Introduction of the study team, including key point of contacts at coordinating centers for questions regarding the MI counseling intervention or TMR intervention.
- Overview of A191901 protocol, including background/rationale, study objectives, schema, key eligibility criteria, statistical considerations, and intervention overview for each arm of the study.
- Text-messaging reminder training, including key information on troubleshooting text message reminders, and other relevant topics.
- Training on setting up the smart phones that are provided (for patients receiving study-provided smart phones) and how to orient the participant to basic mechanics of the device.
- Medication adherence monitoring (Pillsy) cap training, including procedures for ordering caps from the central pharmacy, setting up and assigning the cap to a patient, downloading the Pillsy app onto participant's phone, syncing the cap to the Pillsy app, troubleshooting, disposing of the cap after participation ends, and if collected in clinic, where to send cap after participation ends.
- ePRO app training, including downloading the ePRO app onto the participant's phone, and reviewing the ePRO app with participants.

Time will be provided at the end of the training for any questions site staff may have regarding the protocol or operating procedures. Additionally, site staff can use all training documents provided prior to the activation training for future reference.

After completion of the site activation training, the site staff will receive the Site Information Form, signed by the Central Project Manager, attesting to the site's completion of protocol specific training. Once received, the site staff will upload the signed Site Information Form to CTSU.

13.0 REFERENCES

1. Society, A.C., *Breast cancer facts & figures 2017–2018*. 2017, American Cancer Society Atlanta, GA.
2. *Tamoxifen for early breast cancer: an overview of the randomised trials. Early Breast Cancer Trialists' Collaborative Group.* Lancet, 1998. **351**(9114): p. 1451-67.
3. Gray, R.G., et al., *aTTom: Long-term effects of continuing adjuvant tamoxifen to 10 years versus stopping at 5 years in 6,953 women with early breast cancer*. 2013, American Society of Clinical Oncology.
4. Davies, C., et al., *Long-term effects of continuing adjuvant tamoxifen to 10 years versus stopping at 5 years after diagnosis of oestrogen receptor-positive breast cancer: ATLAS, a randomised trial.* The Lancet, 2013. **381**(9869): p. 805-816.
5. Cheung, K.L., *Endocrine therapy for breast cancer: an overview.* Breast, 2007. **16**(4): p. 327-43.
6. Hershman, D.L., et al., *Early discontinuation and nonadherence to adjuvant hormonal therapy in a cohort of 8,769 early-stage breast cancer patients.* J Clin Oncol, 2010. **28**(27): p. 4120-8.
7. Farias, A.J. and X.L. Du, *Association between out-of-pocket costs, race/ethnicity, and adjuvant endocrine therapy adherence among Medicare patients with breast cancer.* Journal of Clinical Oncology, 2017. **35**(1): p. 86.
8. Partridge, A.H., et al., *Adherence to initial adjuvant anastrozole therapy among women with early-stage breast cancer.* Journal of Clinical Oncology, 2008. **26**(4): p. 556-562.
9. Hershman, D.L., et al., *Early discontinuation and non-adherence to adjuvant hormonal therapy are associated with increased mortality in women with breast cancer.* Breast Cancer Res Treat, 2011. **126**(2): p. 529-37.
10. Farias, A.J. and X.L. Du, *Racial differences in adjuvant endocrine therapy use and discontinuation in association with mortality among Medicare breast cancer patients by receptor status.* Cancer Epidemiology and Prevention Biomarkers, 2017. **26**(8): p. 1266-1275.
11. Group, E.B.C.T.C., *Effects of chemotherapy and hormonal therapy for early breast cancer on recurrence and 15-year survival: an overview of the randomised trials.* The Lancet, 2005. **365**(9472): p. 1687-1717.
12. Demissie, S., R.A. Silliman, and T.L. Lash, *Adjuvant tamoxifen: predictors of use, side effects, and discontinuation in older women.* Journal of Clinical Oncology, 2001. **19**(2): p. 322-328.
13. Wheeler, S.B., et al., *Endocrine therapy adherence, side effects, and risk perception among racially diverse breast cancer patients.* Journal of Clinical Oncology, 2015. **33**(15_suppl): p. 6560-6560.
14. Pinheiro, L.C., et al., *Investigating Associations Between Health-Related Quality of Life and Endocrine Therapy Underuse in Women With Early-Stage Breast Cancer.* Journal of Oncology Practice, 2017. **13**(5): p. e463-e473.
15. Fallowfield, L.J., et al., *Assessment of quality of life in women undergoing hormonal therapy for breast cancer: validation of an endocrine symptom subscale for the FACT-B.* Breast Cancer Res Treat, 1999. **55**(2): p. 189-99.
16. Brett, J., et al., *Factors associated with intentional and unintentional non-adherence to adjuvant endocrine therapy following breast cancer.* Eur J Cancer Care (Engl), 2018. **27**(1).
17. Kimmick, G., et al., *Medication taking behaviors among breast cancer patients on adjuvant endocrine therapy.* The Breast, 2015. **24**(5): p. 630-636.
18. Neugut, A.I., et al., *A prospective cohort study of early discontinuation of adjuvant chemotherapy in women with breast cancer: the breast cancer quality of care study (BQUAL).* Breast Cancer Res Treat, 2016. **158**(1): p. 127-138.
19. Bright, E.E., et al., *Barriers to and facilitative processes of endocrine therapy adherence among women with breast cancer.* Breast cancer research and treatment, 2016. **158**(2): p. 243-251.
20. Walker, H.E., et al., *Perceptions, Attributions, and Emotions Toward Endocrine Therapy in Young Women with Breast Cancer.* J Adolesc Young Adult Oncol, 2016. **5**(1): p. 16-23.

21. Neugut, A.I., et al., *Association between prescription co-payment amount and compliance with adjuvant hormonal therapy in women with early-stage breast cancer.* Journal of Clinical Oncology, 2011. **29**(18): p. 2534.
22. Riley, G.F., et al., *Endocrine therapy use among elderly hormone receptor-positive breast cancer patients enrolled in Medicare Part D.* Medicare & medicaid research review, 2011. **1**(4).
23. Ekinci, E., et al., *Interventions to improve endocrine therapy adherence in breast cancer survivors: what is the evidence?* Journal of Cancer Survivorship, 2018. **12**(3): p. 348-356.
24. Roberts, M.C., S.B. Wheeler, and K. Reeder-Hayes, *Racial/Ethnic and socioeconomic disparities in endocrine therapy adherence in breast cancer: a systematic review.* Am J Public Health, 2015. **105 Suppl 3**: p. e4-e15.
25. Partridge, A.H., et al., *Nonadherence to adjuvant tamoxifen therapy in women with primary breast cancer.* Journal of Clinical Oncology, 2003. **21**(4): p. 602-606.
26. Wheeler, S.B., et al., *Oncology providers' perspectives on endocrine therapy prescribing and management.* Patient preference and adherence, 2016. **10**: p. 2007.
27. Levy, A.E., et al., *Recent Approaches to Improve Medication Adherence in Patients with Coronary Heart Disease: Progress Towards a Learning Healthcare System.* Curr Atheroscler Rep, 2018. **20**(1): p. 5.
28. Palacio, A., et al., *Motivational Interviewing Improves Medication Adherence: a Systematic Review and Meta-analysis.* J Gen Intern Med, 2016. **31**(8): p. 929-40.
29. Zomahoun, H.T.V., et al., *Effectiveness of motivational interviewing interventions on medication adherence in adults with chronic diseases: a systematic review and meta-analysis.* Int J Epidemiol, 2017. **46**(2): p. 589-602.
30. Golin, C.E., et al., *SafeTalk, a multicomponent, motivational interviewing-based, safer sex counseling program for people living with HIV/AIDS: a qualitative assessment of patients' views.* AIDS patient care and STDs, 2010. **24**(4): p. 237-245.
31. Adamian, M.S., et al., *Brief motivational interviewing to improve adherence to antiretroviral therapy: development and qualitative pilot assessment of an intervention.* AIDS Patient Care STDS, 2004. **18**(4): p. 229-38.
32. Rollnick, S., W.R. Miller, and C. Butler, *Motivational interviewing in health care: helping patients change behavior*. 2008: Guilford Press.
33. Fuller, R.H., et al., *Improving medication adherence in patients with cardiovascular disease: a systematic review.* Heart, 2018. **104**(15): p. 1238-1243.
34. Adler, A.J., et al., *Mobile phone text messaging to improve medication adherence in secondary prevention of cardiovascular disease.* Cochrane Database Syst Rev, 2017. **4**: p. CD011851.
35. Viswanathan, M., et al., *Interventions to improve adherence to self-administered medications for chronic diseases in the United States: a systematic review.* Ann Intern Med, 2012. **157**(11): p. 785-95.
36. Fisher, J.D., et al., *The information-motivation-behavioral skills model of antiretroviral adherence and its applications.* Curr HIV/AIDS Rep, 2008. **5**(4): p. 193-203.
37. Bosworth, H.B., et al., *Medication adherence: a call for action.* Am Heart J, 2011. **162**(3): p. 412-24.
38. Wheeler, S.B., K.E. Reeder-Hayes, and L.A. Carey, *Disparities in breast cancer treatment and outcomes: biological, social, and health system determinants and opportunities for research.* Oncologist, 2013. **18**(9): p. 986-93.
39. White-Means, S.I. and A.R. Osmani, *Racial and Ethnic Disparities in Patient-Provider Communication With Breast Cancer Patients: Evidence From 2011 MEPS and Experiences With Cancer Supplement.* Inquiry, 2017. **54**: p. 46958017727104.
40. Wells, K.J., et al., *Barriers and facilitators to endocrine therapy adherence among underserved hormone-receptor-positive breast cancer survivors: a qualitative study.* Supportive Care in Cancer, 2016. **24**(10): p. 4123-4130.

41. Emmons, K.M. and S. Rollnick, *Motivational interviewing in health care settings. Opportunities and limitations.* Am J Prev Med, 2001. **20**(1): p. 68-74.
42. McCowan, C., et al., *Cohort study examining tamoxifen adherence and its relationship to mortality in women with breast cancer.* Br J Cancer, 2008. **99**(11): p. 1763-8.
43. Rosenberg, S.M. and A.H. Partridge, *New insights into nonadherence with adjuvant endocrine therapy among young women with breast cancer*. 2015, Oxford University Press US.
44. Mougalian, S.S., et al., *Bidirectional Text Messaging to Monitor Endocrine Therapy Adherence and Patient-Reported Outcomes in Breast Cancer.* JCO Clin Cancer Inform, 2017. **1**: p. 1-10.
45. de Vries, S.T., et al., *Interest in a mobile app for two-way risk communication: a survey study among European healthcare professionals and patients.* Drug safety, 2018. **41**(7): p. 697-712.
46. Reeder-Hayes, K.E., et al., *Racial disparities in initiation of adjuvant endocrine therapy of early breast cancer.* Breast Cancer Res Treat, 2014. **145**(3): p. 743-51.
47. Wheeler, S.B., et al., *Endocrine therapy initiation among Medicaid-insured breast cancer survivors with hormone receptor-positive tumors.* Journal of Cancer Survivorship, 2014. **8**(4): p. 603-610.
48. Reeder-Hayes, K.E., et al., *Influence of provider factors and race on uptake of breast cancer gene expression profiling.* Cancer, 2018. **124**(8): p. 1743-1751.
49. Cegala, D.J. and D.M. Post, *The impact of patients' participation on physicians' patient-centered communication.* Patient education and counseling, 2009. **77**(2): p. 202-208.
50. Cegala, D.J. and D.M. Post, *On addressing racial and ethnic health disparities: The potential role of patient communication skills interventions.* American Behavioral Scientist, 2006. **49**(6): p. 853-867.
51. Cegala, D.J., D.M. Post, and L. McClure, *The effects of patient communication skills training on the discourse of older patients during a primary care interview.* J Am Geriatr Soc, 2001. **49**(11): p. 1505-11.
52. Post, D.M., D.J. Cegala, and T.M. Marinelli, *Teaching patients to communicate with physicians: the impact of race.* J Natl Med Assoc, 2001. **93**(1): p. 6-12.
53. Krok-Schoen, J.L., et al., *Increasing adherence to adjuvant hormone therapy among breast cancer patients: A smart phone app-based pilot study.* Journal of Clinical Oncology, 2018. **36**(15_suppl): p. e12523-e12523.
54. Parker, C.S., et al., *Adherence to warfarin assessed by electronic pill caps, clinician assessment, and patient reports: results from the IN-RANGE study.* J Gen Intern Med, 2007. **22**(9): p. 1254-9.
55. Shuter, J., et al., *Sequential antiretroviral adherence measurement using electronic bottle cap monitors in a cohort of HIV-infected adults.* Journal of the International Association of Physicians in AIDS Care, 2012. **11**(2): p. 94-97.
56. Clifford, S., et al., *A systematic literature review of methodologies used to assess medication adherence in patients with diabetes.* Current Medical Research and Opinion, 2014. **30**(6): p. 1071-1085.
57. Williams, A.B., et al., *A proposal for quality standards for measuring medication adherence in research.* AIDS Behav, 2013. **17**(1): p. 284-97.
58. Hudis, C.A., et al., *Proposal for standardized definitions for efficacy end points in adjuvant breast cancer trials: The STEEP system.* Journal of Clinical Oncology, 2007. **25**(15): p. 2127-2132.
59. Lundahl, B., et al., *Motivational interviewing in medical care settings: a systematic review and meta-analysis of randomized controlled trials.* Patient Educ Couns, 2013. **93**(2): p. 157-68.
60. Smedslund, G., et al., *Motivational interviewing for substance abuse.* Cochrane Database Syst Rev, 2011(5): p. CD008063.
61. Golin, C.E., et al., *A 2-arm, randomized, controlled trial of a motivational interviewing–based intervention to improve adherence to antiretroviral therapy (ART) among patients failing or initiating ART.* Journal of acquired immune deficiency syndromes (1999), 2006. **42**(1): p. 42.
62. Ward, E., et al., *Cancer disparities by race/ethnicity and socioeconomic status.* CA: a cancer journal for clinicians, 2004. **54**(2): p. 78-93.

63. Bach, P.B., et al., *Survival of blacks and whites after a cancer diagnosis.* Jama, 2002. **287**(16): p. 2106-2113.
64. Pollitt, R.A., et al., *Examining the pathways linking lower socioeconomic status and advanced melanoma.* Cancer, 2012. **118**(16): p. 4004-4013.
65. Hays, R.D., et al., *Development of physical and mental health summary scores from the patient-reported outcomes measurement information system (PROMIS) global items.* Quality of Life Research, 2009. **18**(7): p. 873-880.
66. Stanton, A.L., C.A. Bernaards, and P.A. Ganz, *The BCPT symptom scales: a measure of physical symptoms for women diagnosed with or at risk for breast cancer.* Journal of the National Cancer Institute, 2005. **97**(6): p. 448-456.
67. Alfano, C.M., et al., *Psychometric properties of a tool for measuring hormone-related symptoms in breast cancer survivors.* Psycho-Oncology: Journal of the Psychological, Social and Behavioral Dimensions of Cancer, 2006. **15**(11): p. 985-1000.
68. Cella, D., et al., *The Patient-Reported Outcomes Measurement Information System (PROMIS) developed and tested its first wave of adult self-reported health outcome item banks: 2005–2008.* Journal of clinical epidemiology, 2010. **63**(11): p. 1179-1194.
69. Vickberg, S.M.J., *The Concerns About Recurrence Scale (CARS): a systematic measure of women's fears about the possibility of breast cancer recurrence.* Annals of Behavioral Medicine, 2003. **25**(1): p. 16-24.
70. van den Beuken-van Everdingen, M.H., et al., *Concerns of former breast cancer patients about disease recurrence: a validation and prevalence study.* Psycho-Oncology: Journal of the Psychological, Social and Behavioral Dimensions of Cancer, 2008. **17**(11): p. 1137-1145.
71. Momino, K., et al., *Psychometric properties of the Japanese version of the Concerns About Recurrence Scale (CARS-J).* Japanese journal of clinical oncology, 2014. **44**(5): p. 456-462.
72. Cohen, S., T. Kamarck, and R. Mermelstein, *A global measure of perceived stress.* Journal of health and social behavior, 1983: p. 385-396.
73. Cohen, S., *Perceived stress in a probability sample of the United States.* 1988.
74. Moser, A., et al., *The eight-item modified Medical Outcomes Study Social Support Survey: psychometric evaluation showed excellent performance.* Journal of clinical epidemiology, 2012. **65**(10): p. 1107-1116.
75. Cameron, K.A., et al., *Measuring patients' self-efficacy in understanding and using prescription medication.* Patient education and counseling, 2010. **80**(3): p. 372-376.
76. Pérez-Escamilla, B., et al., *Identification of validated questionnaires to measure adherence to pharmacological antihypertensive treatments.* Patient preference and adherence, 2015. **9**: p. 569.
77. Lavsa, S.M., A. Holzworth, and N.T. Ansani, *Selection of a validated scale for measuring medication adherence.* Journal of the American Pharmacists Association, 2011. **51**(1): p. 90-94.
78. Kim, C.-J., et al., *Evaluation of the measurement properties of self-reported medication adherence instruments among people at risk for metabolic syndrome: a systematic review.* The Diabetes Educator, 2016. **42**(5): p. 618-634.
79. Beyhaghi, H., et al., *Psychometric properties of the four-item morisky green levine medication adherence scale among atherosclerosis risk in communities (ARIC) study participants.* Value in Health, 2016. **19**(8): p. 996-1001.
80. Weiner, B.J., et al., *Psychometric assessment of three newly developed implementation outcome measures.* Implementation Science, 2017. **12**(1): p. 108.
81. Damschroder, L.J., et al., *Fostering implementation of health services research findings into practice: a consolidated framework for advancing implementation science.* Implementation science, 2009. **4**(1): p. 50.

APPENDIX I: PATIENT MAILING AND CONTACT INFORMATION FORM

Patient Contact Information Form

Instructions: Complete all participant information regardless of randomization assignment at the time of the Screening & Consent visit. After visit and randomization are complete, fill in the site staff section at the bottom of the form. **Both pages of the form should be faxed to the OSU Project Coordinator at (614) 293-2654.** If you have questions, please contact via phone the OSU Project Coordinator at (614) 293-8006.

1. Patient Name *(required)*: ____

2. Mailing Address *(required)*: ____
   Street Address

   ____
   City State Zip

3. County *(required)*: ______________________________

4. Email Address *(required)*: ____

5. Preferred Time Zone *(required)*:
   - ☐ Eastern Time UTC -5:00/-4:00
   - ☐ Central Time UTC -6:00/-5:00
   - ☐ Mountain Time UTC -7:00/-6:00
   - ☐ Pacific Time UTC-8:00/-7:00

6. Mobile Phone Number *(required)*: (______)_______–________

7. Mobile Phone Carrier *(required)*: ____

8. Alternate Phone Number #1 *(required)*: (______)_______–________

   Type____________Name:_____________

9. Alternate Phone Number #2 *(optional)*: (_______)_______–________

   Type____________Name:_____________

10. If randomized to daily text messages, preferred time to receive texts *(required)*: _________
    (Note: 10:00am in preferred time zone is the default if patient has no preference)

11. Date of Birth *(required)*: ___/____/________(mm/dd/yyyy)

12. Race (select all that apply, *required*):

    ☐White

    ☐Black or African-American

    ☐Native Hawaiian or other Pacific Islander

☐American Indian or Alaska Native

☐Asian

☐Other, please specify: ____________________

13. Have you started taking your endocrine therapy medication yet *(required)*? ☐Yes ☐No

(required) If yes, approximately when did you start? ___/____/________ (mm/dd/yyyy)

(required) If no, patient must start 6 weeks from randomization, which is on: ___/_____/_______

THIS PORTION TO BE COMPLETED BY SITE STAFF AFTER CONSENT VISIT (page 2 of 2)

Instructions: Complete this section of the form after the participant has been registered and randomized. Afterwards, **fax completed form to the OSU project coordinator at (614) 293-2654**. If you have questions, please contact via phone the OSU project coordinator at (614) 293-8006.

Site CTEP Code: ______________

Site Contact Name: __________________________________

Email: _____________________ Phone: (_____) ______-__________

A191901 Participant Study ID: ________________

Date of Randomization: ______________

Randomization Assignment:
☐Arm 1: Text-Message Reminder (TMR)
☐Arm 2: Motivational Interviewing Counseling (MI)
☐Arm 3: TMR + MI
☐Arm 4: Enhanced Usual Care

APPENDIX II: ALLIANCE PATIENT QUESTIONNAIRE – SOCIO-DEMOGRAPHICS

1. What is your age (in years)? __________

2. What is your gender?
   ___ Male
   ___ Female
   ___ Non-binary/third gender
   ___ Don't know
   ___ I prefer to self-describe: ___________
   ___ I prefer not to answer

3. Are you of Hispanic, Latino, or Spanish in origin?
   ___ Yes
   ___ No
   ___ Don't know
   ___ I prefer not to answer

4. What is your race? Select all that apply.
   ___ Black or African American
   ___ Native Hawaiian or other Pacific Islander
   ___ American Indian or Alaska Native
   ___ White
   ___ Asian
   ___ Other, please tell us: ____________
   ___ Don't know
   ___ I prefer not to answer

5. Do you speak a language other than English at home?
   ___ Yes →
   ___ No
   ___ Don't know
   ___ I prefer not to answer

5a. What is this language?
   ___ Spanish
   ___ Other, please specify: __________

5b. How well do you speak English?
   ___ Very well
   ___ Well
   ___ Not well
   ___ Not at all

6. What is the highest level of education that you completed?
   ___ Less than 9 Years of school
   ___ Some high school (9-11 years)
   ___ High school graduate, or GED
   ___ Some college or technical school
   ___ College graduate
   ___ Post graduate education, but no higher degree
   ___ Graduate degree
   ___ Don't know
   ___ I prefer not to answer

7. Are you now…
   ___ Married
   ___ Domestic partnership
   ___ Widowed
   ___ Divorced
   ___ Separated
   ___ Never Married
   ___ Don't know
   ___ I prefer not to answer

8. Do you currently have health insurance coverage?
   ___ Yes
   ___ No
   ___ Don't know
   ___ I prefer not to answer

9. If you answered 'Yes' to question 8, what kind(s) of health insurance or health coverage do you have? Mark all that apply
   ___ Private health insurance
   ___ Medicare
   ___ Medi-Gap
   ___ Medicaid
   ___ Military health care (TRICARE, VA, CHAMP-VA)
   ___ State-sponsored health plan
   ___ Single service plan (for example, dental, vision, prescriptions)
   ___ Other, please tell us: __________________
   ___ Don't know
   ___ I prefer not to answer

10. How many people are in your household (including yourself)?
   ____________ people
   ___ Don't know
   ___ I prefer not to answer

11. Are you the primary caregiver to at least 1 dependent child or adult living in your household?
    ___ Yes
        If yes, how many dependent children or adults live in your household? ___
    ___ No

12. What was the total combined income of your household in the past year, including income from all sources such as wages, salaries, Social Security or retirement benefits, help from relatives and so forth? Please tell us the total income before taxes.
    ___Less than $20,000
    ___$20,000 to $49,999
    ___$50,000 to $89,999
    ___$90,000 to $119,999
    ___$120,000 or above
    ___Don't know
    ___I prefer not to answer

13. How would you describe your current employment situation?

    Please choose the ONE that best describes your employment situation.

    NOT CURRENTLY WORKING

    ___ On paid sick leave
    ___ On unpaid sick leave
    ___ On disability
    ___ Retired
    ___ Not employed – looking for a job
    ___ Not employed – not looking for a job
    ___ Not working – other

    CURRENTLY WORKING

    ___ Currently working in at least one FULL time job (35 hours or more per week)
    ___ Currently working in at least one PART time job (less than 35 hours per week)

APPENDIX III: PERSONAL HEALTH HISTORY

1. I would describe my menopausal status as…
   ___ Premenopausal (menstrual periods have not stopped OR not sure whether menstrual periods have stopped
   ___ Postmenopausal (menstrual periods have stopped)

2. How many times have you been pregnant in your lifetime? _________

3. How many biological children have you given birth to? _________

4. Were any of your grandparents Ashkenazi Jewish descent? (A person of Ashkenazi Jewish descent is someone having origins in any of the original Jewish people of Eastern Europe.)
   ___ Yes
   ___ No
   ___ Don't know
   ___ I prefer not to answer

5. Has your biological mother been diagnosed with…
   Breast Cancer?
      ___ Yes
      ___ No
      ___ Don't know
      ___ I prefer not to answer
   Ovarian cancer?
      ___ Yes
      ___ No
      ___ Don't know
      ___ I prefer not to answer

6. Do you have any biological sisters or biological daughters?
      ___ Yes
      ___ No
      ___ Don't know
      ___ I prefer not to answer

   If yes, have any of your sister(s)/daughter(s) been diagnosed with…
      Breast Cancer?
         ___ Yes
         ___ No
         ___ Don't know
         ___ I prefer not to answer

Ovarian cancer?
___ Yes
___ No
___ Don't know
___ I prefer not to answer

7. Have any of your second- or third-degree female blood relatives had breast or ovarian cancer? (Second- or third-degree relatives include cousins, aunts, grandmothers, and nieces on either your father or mother's side.)
___ Yes
___ No
___ Don't know
___ I prefer not to answer

APPENDIX IV: PROMIS GLOBAL HEALTH QUESTIONNAIRE V1.2

PROMIS® Scale v1.2 – Global Health

Global Health

Please respond to each question or statement by marking one box per row.

		Excellent	Very good	Good	Fair	Poor
Global01	In general, would you say your health is:	☐ 5	☐ 4	☐ 3	☐ 2	☐ 1
Global02	In general, would you say your quality of life is:	☐ 5	☐ 4	☐ 3	☐ 2	☐ 1
Global03	In general, how would you rate your physical health?	☐ 5	☐ 4	☐ 3	☐ 2	☐ 1
Global04	In general, how would you rate your mental health, including your mood and your ability to think?	☐ 5	☐ 4	☐ 3	☐ 2	☐ 1
Global05	In general, how would you rate your satisfaction with your social activities and relationships?	☐ 5	☐ 4	☐ 3	☐ 2	☐ 1
Global09r	In general, please rate how well you carry out your usual social activities and roles. (This includes activities at home, at work and in your community, and responsibilities as a parent, child, spouse, employee, friend, etc.)......	☐ 5	☐ 4	☐ 3	☐ 2	☐ 1
		**Completely**	**Mostly**	**Moderately**	**A little**	**Not at all**
Global06	To what extent are you able to carry out your everyday physical activities such as walking, climbing stairs, carrying groceries, or moving a chair?	☐ 5	☐ 4	☐ 3	☐ 2	☐ 1

13 April 2018

© 2010-2018 PROMIS Health Organization (PHO)

PROMIS® Scale v1.2 – Global Health

In the past 7 days…

		Never	Rarely	Sometimes	Often	Always
Global10r	How often have you been bothered by emotional problems such as feeling anxious, depressed or irritable?	☐ 5	☐ 4	☐ 3	☐ 2	☐ 1

		None	Mild	Moderate	Severe	Very severe
Global08r	How would you rate your fatigue on average?	☐ 5	☐ 4	☐ 3	☐ 2	☐ 1

Global07r	How would you rate your pain on average?	☐ 0 No pain	☐ 1	☐ 2	☐ 3	☐ 4	☐ 5	☐ 6	☐ 7	☐ 8	☐ 9	☐ 10 Worst pain imaginable

13 April 2018
© 2010-2018 PROMIS Health Organization (PHO) Page 2 of 2

APPENDIX V: BREAST CANCER PREVENTION TRIAL (BCPT) SYMPTOM QUESTIONNAIRE

Circle one number on each line. If you do not have the problem, circle "0 – Not at all".

During the past 4 weeks, how much were you bothered by:

	Not at all	Slightly	Moderately	Quite a bit	Extremely
1. Hot flashes	0	1	2	3	4
2. Nausea	0	1	2	3	4
3. Vomiting	0	1	2	3	4
4. Difficulty with bladder control when laughing or crying	0	1	2	3	4
5. Difficulty with bladder control at other times	0	1	2	3	4
6. Vaginal dryness	0	1	2	3	4
7. Pain with intercourse	0	1	2	3	4
8. General aches and pains	0	1	2	3	4
9. Joint pains	0	1	2	3	4
10. Muscle stiffness	0	1	2	3	4
11. Weight gain	0	1	2	3	4
12. Unhappy with the appearance of my body	0	1	2	3	4
13. Forgetfulness	0	1	2	3	4
14. Night sweats	0	1	2	3	4
15. Difficulty concentrating	0	1	2	3	4
16. Easily distracted	0	1	2	3	4
17. Arm swelling (lymphedema)	0	1	2	3	4
18. Decreased range of motion in arm on surgery side	0	1	2	3	4
19. Vaginal discharge	0	1	2	3	4

20. Vaginal bleeding or spotting	0	1	2	3	4
21. Genital itching/irritation	0	1	2	3	4
22. Lack of energy	0	1	2	3	4
23. Tiredness	0	1	2	3	4
24. Lack of interest in sex	0	1	2	3	4
25. Low sexual enjoyment	0	1	2	3	4

Stanton, A. L., Bernaards, C. A., & Ganz, P. A. (2005). The BCPT Symptom Scales: A measure of physical symptoms for women diagnosed with or at risk for breast cancer. *Journal of the National Cancer Institute*, 97, 448-456.

APPENDIX VI: PROMIS DEPRESSION SHORT FORM 8A V1.0

Emotional Distress – Depression – Short Form 8a

Please respond to each question or statement by marking one box per row.

	In the past 7 days...	Never	Rarely	Sometimes	Often	Always
EDDEP04	I felt worthless	☐ 1	☐ 2	☐ 3	☐ 4	☐ 5
EDDEP06	I felt helpless	☐ 1	☐ 2	☐ 3	☐ 4	☐ 5
EDDEP29	I felt depressed	☐ 1	☐ 2	☐ 3	☐ 4	☐ 5
EDDEP41	I felt hopeless	☐ 1	☐ 2	☐ 3	☐ 4	☐ 5
EDDEP22	I felt like a failure	☐ 1	☐ 2	☐ 3	☐ 4	☐ 5
EDDEP36	I felt unhappy	☐ 1	☐ 2	☐ 3	☐ 4	☐ 5
EDDEP05	I felt that I had nothing to look forward to	☐ 1	☐ 2	☐ 3	☐ 4	☐ 5
EDDEP09	I felt that nothing could cheer me up	☐ 1	☐ 2	☐ 3	☐ 4	☐ 5

26 June 2016
© 2008-2016 PROMIS Health Organization and PROMIS Cooperative Group Page 1 of 1

APPENDIX VII: CONCERNS ABOUT RECURRENCE SCALE (CARS)

Concerns about Recurrence

The following questions ask you to tell us about any worries you may have about the possibility of breast cancer recurrence. By recurrence we mean the breast cancer coming back in the same breast or another area of the body, or a new breast cancer in either breast.

Although most women who have been diagnosed with early stage breast cancer will never have another problem with the cancer, we are aware that many women do worry about this possibility. Other women may not worry about recurrence at all. Either way, your answers to these questions are very important to us. We understand that it may be upsetting to think about or answer questions about the possibility of recurrence. However, we need your help to understand how women think about this possibility.

For the following four questions please circle the number that comes closest to the way you feel. For example, for the first question you should circle "1" if you don't think about recurrence at all, circle "6" if you think about recurrence all the time, or circle "2", "3", "4" or "5" if the amount of time you spend thinking about recurrence is somewhere in between.

1. How much time do you spend thinking about the possibility that your breast cancer could recur?

1	2	3	4	5	6
I Don't Think About It At All					*I Think About It All The Time*

2. How much does the possibility that your breast cancer could recur upset you?

1	2	3	4	5	6
It Does Not Upset Me At All					*It Makes Me Extremely Upset*

3. How often do you worry about the possibility that your breast cancer could recur?

1	2	3	4	5	6
I Never Worry About It					*I Worry About It All The Time*

4. How afraid are you that your breast cancer may recur?

1	2	3	4	5	6
Not At All Afraid					*Very Afraid*

The Concerns About Recurrence Scale
©2002 Suzanne M. Johnson Vickberg, Ph.D.

Scoring

Score the CARS by computing the mean rating of all items in each subscale.

Overall Fear (4 Items): 1, 2, 3, 4

Health Worries (10 Items): 5, 6, 7, 9, 11, 16, 20, 21, 22, 26

Womanhood Worries (7 Items): 8, 12, 18, 23, 25, 28, 30

Role Worries (5 Items): 13, 15, 17, 24, 29

Death Worries (2 Items): 14, 27

The Concerns About Recurrence Scale
©2002 Suzanne M. Johnson Vickberg, Ph.D.

APPENDIX VIII: PERCEIVED STRESS SCALE (PSS)

PSS

INSTRUCTIONS:

The questions in this scale ask you about your feelings and thoughts during THE LAST MONTH. In each case, please indicate your response by placing an "X" over the circle representing HOW OFTEN you felt or thought a certain way.

	Never 0	Almost Never 1	Sometimes 2	Fairly Often 3	Very Often 4
**1. In the last month, how often have you been upset because of something that happened unexpectedly?**	○	○	○	○	○
**2. In the last month, how often have you felt that you were unable to control the important things in your life?**	○	○	○	○	○
**3. In the last month, how often have you felt nervous and "stressed"?**	○	○	○	○	○
**4. In the last month, how often have you felt confident about your ability to handle your personal problems?**	○	○	○	○	○
**5. In the last month, how often have you felt that things were going your way?**	○	○	○	○	○
**6. In the last month, how often have you found that you could not cope with all the things that you had to do?**	○	○	○	○	○
**7. In the last month, how often have you been able to control irritations in your life?**	○	○	○	○	○
**8. In the last month, how often have you felt that you were on top of things?**	○	○	○	○	○
**9. In the last month, how often have you been angered because of things that were outside your control?**	○	○	○	○	○
**10. In the last month, how often have you felt difficulties were piling up so high that you could not overcome them?**	○	○	○	○	○

APPENDIX IX: MODIFIED MEDICAL OUTCOMES STUDY SOCIAL SUPPORT SURVEY (MMOS-SS)

People sometimes look to others for companionship, assistance, or other types of support. How often is each of the following kinds of support available to you if you need it?

(Circle one number on each line)

Tangible Support

If you needed it, how often is someone available…	None of the Time	A Little of the Time	Some of the Time	Most of the Time	All of the Time
1. to help you if you were confined to bed	1	2	3	4	5
2. to take you to the doctor if you needed it	1	2	3	4	5
3. to prepare your meals if you were unable to do it yourself	1	2	3	4	5
4. to help with daily chores if you were sick	1	2	3	4	5

Emotional/Informational Support

If you needed it, how often is someone available…	None of the Time	A Little of the Ti[illegible]	Some of the Time	Most of the Time	All of the Time
1. to have a good time with?	1	2	3	4	5
2. to turn to for suggestions about how to deal with a personal problem?	1	2	3	4	5
3. who understands your problems?	1	2	3	4	5
4. to love and make you feel wanted?	1	2	3	4	5

APPENDIX X: MEDICAL USE SELF-EFFICACY SCALE (MUSE)

Medication Understanding and Use Self-Efficacy Scale

I am going to read you 8 statements to see how confident you are at taking/understanding your medication. Please tell me if you strongly disagree, slightly disagree, slightly agree, or strongly agree.

	**DISAGREE***		**AGREE**	
	**Strongly (1)**	**Slightly (2)**	**Slightly (3)**	**Strongly (4)**
It is easy for me to take my medicine on time. (TM)	1	2	3	4
It is easy for me to ask my pharmacist questions about my medicine. (LM)	1	2	3	4
It is easy for me to understand my pharmacist's instructions for my medicine. (LM)	1	2	3	4
It is easy for me to understand instructions on medicine bottles. (LM)	1	2	3	4
It is easy for me to get all the information I need about my medicine. (LM)	1	2	3	4
It is easy to remember to take all my medicines. (TM)	1	2	3	4
It is easy for me to set a schedule to take my medicines each day. (TM)	1	2	3	4
It is easy for me to take my medicines every day. (TM)	1	2	3	4

*If individuals have difficulty with the four item scale, it can be presented to them in two levels, such that they are asked if they agree or disagree, and then the extent to which they agree or disagree.

(LM) denotes item is part of the "Learning about medication" subscale.

(TM) denotes item is part of the "Taking medication" subscale.

Cameron KA, Ross EL, Clayman ML, Bergeron AR, Federman AD, Bailey SC, Davis TC, Wolf MS. Medication Understanding and Use Self-Efficacy Scale, 2010.

Relevant literature: Cameron KA, Ross EL, Clayman ML, Bergeron AR, Federman AD, Bailey SC, Davis TC, Wolf MS. Measuring patients' self-efficacy in understanding and using prescription medication. *Pat Educ Couns* 2010;80 372-6.

APPENDIX XI: SELF-REPORTED ENDOCRINE THERAPY ADHERENCE AND PERSISTENCE

Many women who have had breast cancer take endocrine therapy pills, also known as hormonal therapy or anti-estrogen therapy, to help keep breast cancer from coming back. Specific names of these drugs are Tamoxifen, Femara (or Letrozole), Aromasin (or Exemestane), and Arimidex (or Anastrozole).

At this time, are you taking endocrine therapy pills?

____ 1. Yes, I'm taking them exactly as prescribed by my doctor
____ 2. Yes, I'm taking them, but not every day
____ 3. No, I stopped taking those pills

If yes, please answer the following questions

Currently, what endocrine therapy medication are you taking?
____ Tamoxifen
____ Femara (or Letrozole)
____ Aromasin (or Exemestane)
____ Arimidex (or Anastrazole)
____Other (please specify: ____________________)

Over the past two weeks, how many days did you miss your endocrine therapy pills? (Mark an "X" in front of one answer)
___ 0 days
___ 1 day
___ 2 days
___ 3 days
___ 4 days
___ 5 or more days

Whether or not you have stopped taking endocrine therapy pills, please answer the following questions. If you are no longer taking endocrine therapy pills, answer these questions about the time when you were taking them.

At any time since you began taking Endocrine Therapy, have you ever changed the type of medication you are taking?

____ 1. YES
____ 2. NO

When you fill your endocrine therapy prescription, do you USUALLY get a 30-day supply (30 pills) or a 90-day supply (90 pills)?

____ 1. 30-day Supply

____ 2. 90-day Supply

Have you ever cut back or stop taking your endocrine therapy pills because these pills made you feel bad?

____ 1. YES

____ 2. NO

Many people forget to take their pills when they are away from home. Have you forgotten to take your endocrine therapy pills when you were away from home?

____ 1. Yes, I've sometimes forgotten to take my pills when away from home

____ 2. No, I always remember to take my pills, even when I'm away from home

Taking pills every day can be hard. For you, is sticking to your endocrine therapy treatment plan…
(Mark an "X" in front of one answer)

____1. Very hard

____2. Somewhat hard

____3. Not hard at all

Would you say you have trouble remembering to take your endocrine therapy pills…

____ 1. Often

____ 2. Sometimes

____ 3. Practically never

For you, how often is it true that you miss your endocrine therapy pills because…

(Circle one number per line)

	Often true for you	Sometimes true for you	Practically never true for you
The pills are too expensive.	1	2	3
You just forget to take them.	1	2	3
You don't get around to refilling the prescription.	1	2	3
The side effects of these pills are too hard to deal with.	1	2	3

Taking medication for a long time is not healthy.	1	2	3

Think about the whole process of taking these endocrine therapy pills—from filling the prescription to taking the pills every day and putting up with the side effects from them. Do you think… (Mark an "X" in front of one answer)

____1. The good outweighs the bad

____2. The good and bad are equal

____3. The bad outweighs the good

Suppose you took all your endocrine therapy pills as prescribed. What do you think the chance would be that your cancer would ever come back? (Mark an "X" in front of one answer)

____ 1. Very low chance

____ 2. Low chance

____ 3. Moderate chance

____ 4. High chance

____ 5. Very high chance

Suppose you stopped taking these pills as prescribed. What do you think would happen to your chances of your breast cancer coming back? (Mark an "X" in front of one answer)

____ 1. My chances would go up a lot

____ 2. My chances would go up a little

____ 3. My chances would not really change

APPENDIX XII: INTERVENTION EVALUATION: ACCEPTABILITY, PATIENT SATISFACTION, AND APPROPRIATENESS

	**Completely Disagree**	**Disagree**	**Neither Agree Nor Disagree**	**Agree**	**Completely Agree**
Acceptability of Intervention Measure (AIM)					
The GET SET program met my approval	1	2	3	4	5
Participating in the GET SET program was appealing to me.	1	2	3	4	5
I liked the GET SET program	1	2	3	4	5
I welcomed the GET SET program in my life	1	2	3	4	5
Intervention Appropriateness Measure (IAM)					
The GET SET program seemed fitting for me	1	2	3	4	5
The GET SET program seemed suitable for me	1	2	3	4	5
The GET SET program seemed applicable to me	1	2	3	4	5
The GET SET program seemed like a good match for me	1	2	3	4	5
Ease of Use and Willingness to Recommend GET SET					
The GET SET print materials were visually appealing and easy to use	1	2	3	4	5
The GET SET website was visually appealing and easy to use	1	2	3	4	5
The Pillsy® Smart Cap that was given to me for the study was easy to use	1	2	3	4	5
The GET SET program was convenient for me	1	2	3	4	5
I felt that participating in the GET SET program made it easier for me to stay on my endocrine therapy pills	1	2	3	4	5
The GET SET program addressed issues that were important to me	1	2	3	4	5
I felt that participating in the GET SET program made it easier for me to talk to my doctor about my endocrine therapy pills	1	2	3	4	5

APPENDIX XIII: WAS IT WORTH IT (WIWI) QUESTIONNAIRE

Participating in a clinical trial/research study is a personal choice and an individual experience. We would like to get your feedback on your experience in this research study.

Directions: Please mark one response for each of the following questions.

1. **Was it worthwhile for you to participate in this research study?**

   ☐ Yes

   ☐ No

   ☐ Uncertain

2. **If you had to do it over, would you participate in this research study again?**

   ☐ Yes

   ☐ No

   ☐ Uncertain

3. **Would you recommend participating in this research study to others?**

   ☐ Yes

   ☐ No

   ☐ Uncertain

4. **Overall, did your quality of life change by participating in this research study?**

   ☐ It improved

   ☐ It stayed the same

   ☐ It got worse

5. **Overall, how was your experience of participating in this research study?**

   ☐ Better than I expected

   ☐ The same as I expected

   ☐ Worse than I expected

APPENDIX XIV: TEXT MESSAGE REMINDER (TMR) MESSAGES AND INTERACTIVE MONTHLY QUESTIONS

The following is a list of the text message reminder (TMR) messages that will be used:

- This is a friendly reminder to take your medication!
- Time to take your medication.
- Spring into action and take your medication!
- Your medication says, "Take me know please."
- Hand to pills to mouth time.
- Stop, drop and pop your pills!
- Don't forget to take your medication today.
- Stop everything! It's medication time!
- Your medication says, "Find me and take me!"
- The thing about remembering to take your medication is that you don't forget.
- It's a great time to take your medication.
- She places her medication in her mouth, big gulp…Score!
- I'm reminding you to take your medication.
- Step to the left, grab your medication. Step to the right, take your medication.
- Did you remember? Time to take your medication today!
- Hop, skip and jump to take your medication!
- It's a great day to take your medication!
- Don't forget! Time to take your medication.
- It's important to take your medication today.
- It's that time again. Take your medication!
- What time is it? It's medication time!
- Ready, set, go! Take your medication!
- Taking your medication = taking care of yourself.
- Hydrate yourself with a nice glass of water. Take your pills while you're at it!
- It's time to take your pills!
- It's part of your daily routine to take your pills. Go do it now!
- Time out! Take your pills. Resume life!
- Pause. It's time to take your medication.
- Real quick – take your pills.
- If you have a minute, go take your pills!
- Knock Knock – who's there? It's time. It's time who? Time to take your medication!
- Don't take a vacation from your medication!

- Ahora! Maintenant! 现在! It's time…to take your medication!
- Ready for a taste of your own medicine? Take your pills!
- If the pill fits, take it!
- Practice makes perfect! It's time to take your pills.
- Check me off your to-do list! Take your pills.
- Let's do this, take your pills!
- We interrupt your regularly scheduled program to bring you…your medication reminder!
- Time to take your pills! We now return to your regularly scheduled programming.
- Put your best self forward. Take your medication.
- She's nearing the goal, takes her medication, and score!
- Let's go get that pill bottle.
- Seize the day and your medication!
- Down the hatch! Time to take your pills.
- Guess what? It's medication time!
- It's time to open that bottle and take your medication.
- It's that all-important medication time again!
- VIP – Very Important Pill time!
- Medication + Hydration. It's a great combination!
- Well, look at the time! It's time to take your pills.
- The time has come…to take your pills!
- I'm prompting you to take your medication.
- This is your cue: time to take your pills.
- I suggest you take your medication today.
- I encourage you to take your medication today!
- Reminder: take your pills. Have a great day!
- Just a little reminder to take your medication.
- Shake, shake, shake! Grab that pill bottle! It's time to take your medication.
- Next item on the agenda: take your medication!
- Medication reminder! Now is the time.
- Daily notification: time to take your medication.

The following is a list of encouraging messages that may be used in response to the Monthly Questions:

- Well done!
- Keep up the good work!
- Way to go!

- Nice job!
- Keep it up!
- Fantastic!
- Great job staying on track!
- Wonderful!
- Sweet!
- You’re taking care of yourself!
- Awesome!
- Keep the streak alive!
- You’re doing a great job!
- You got this!
- Excellent!

**Participant Response**	**System Reply (Actionable Approach)**
Delay in starting/filling prescription	Please try to start taking your medication as prescribed by your physician.
My doctor told me to stop taking the medication for a while.	Ok, thank you for letting us know. We will continue to send you the daily text reminders to take your medication. You can just ignore them for now. We will send you another survey next month to see if you have started back on your medication by then.
I forgot	I forgot → That happens to all of us. Some ways to help you remember to take your medication are: 1) use a pill box; 2) establish a routine of taking your medication at the same time every day; or 3) place your medication in a location where you can’t miss it.
Symptoms (with interactive feedback tailored to response)	Have you talked to your doctor about this? → Yes → Great job!<br>**<u>OR</u>**<br>Have you talked to your doctor about this? → No → Please talk to your physician about the symptoms you are having. The doctor can help. Write down the symptoms you are having, when they occur, and how much they bother you, so that you can better describe your symptoms to your physician. Your doctor wants to help you manage these symptoms, so that you can take your medication regularly!
Health insurance doesn’t cover it	Many patients have this problem. Please talk to your health care team about this. They may be able to help you. Be specific about what expenses your health insurance doesn’t cover and any other insurance problems that you are having.
Medication/co-payments cost too much	Please talk to your health care team about this. They may be able to help you find a medication that has a lower cost or co-payment.

Family/friends discouraged me from taking medication	That happens sometimes, but taking this medication is an investment in your health. Please talk to your doctor or nurse about the concerns of your family or friends, and the importance of taking your medication every day.
I am taking too many medications	Please talk to your doctor or nurse about this and the number of medications you take. They may be able to help you. When you talk to them, tell them your concerns, and how you feel about being on so many medications.
I don't like taking medications	That's understandable. Many people feel this way, however, taking this medication is an investment in your health. Please talk to your doctor or nurse about your concerns. Think about what might make you more willing to take your medication every day.
Other (if participant response does not fall into a category listed above)	Please talk to your health care team about this. They may be able to help you and/or find you resources for this concern.

APPENDIX XV: ALLIANCE EPRO

a. Introduction

Electronic collection is mandatory for this study. Patients will need to use their own device (IOS, Android phone or tablet). If a participant does not own a smart phone, has limited data or texting capabilities, or their smart phone cannot support the iMedidata Patient Cloud app, a smart phone and service can be provided to the participant at no cost. Short term data will only appear on the patient's device until responses are completed. The patient data will import directly into the Rave database once the patient clicks the "submit" button and will no longer be on the patient's device.

Site users of ePRO and the Patient Cloud require the same access as those using Rave. Access to the trial in the Patient Cloud is granted through the iMedidata application to all persons with the appropriate roles assigned in Regulatory Support System (RSS). To access the Patient Cloud via iMedidata, the site user must have an active CTEP-IAM account and the appropriate Rave role (Rave CRA, Read-Only, Site Investigator) on either the LPO or participating organization roster at the enrolling site.

Upon initial site registration approval for the study in RSS, all persons with Rave roles assigned on the appropriate roster will be sent a study invitation e-mail from iMedidata. To accept the invitation, site users must log into the Select Login using their CTEP-IAM username and password, and click on the "accept" link in the upper right corner of the iMedidata page. Please note, site users will not be able to access the study in the Patient Cloud until all required Medidata and study specific trainings are completed. Trainings will be in the form of electronic learnings (eLearnings), and can be accessed by clicking on the link in the upper right pane of the iMedidata screen.

Users who have not previously activated their iMedidata/Rave account at the time of initial site registration approval for the study in RSS will also receive a separate invitation from iMedidata to activate their account. Account activation instructions are located on the CTSU website, Rave tab under the Rave resource materials (Medidata Account Activation and Study Invitation Acceptance).

Additional information on iMedidata/Rave is available on the CTSU members' website under the Rave tab at www.ctsu.org/RAVE/ or by contacting the CTSU Help Desk at 1-888-823-5923 or by e-mail at ctsucontact@westat.com.

b. CRP Training for ePRO

Please visit the Medidata Learning Tool for reference information on Patient Cloud ePRO for CRPs.

c. Registration Instructions for ePRO

Please visit the Medidata Learning Tool for additional screenshots and video tutorials on how to register participants to the study.

i. The subject registration process starts in iMedidata. Begin by clicking on the Patient Cloud Registration link for your study
ii. The patient management app will display. Select your STUDY and SITE from the drop downs and click Launch.
iii. Now you can register your first patient. Create a subject ID and select a Country / Language from the drop down (these are the only required data fields). The subject initials are optional but may help in identifying which subject ID maps with which activation code. When finished, click Add.
iv. The subject added will appear at the top of the table as seen in the picture below and will include the date the patient was added, the subject ID, initials (if included) and a unique auto-generated activation code. The activation code is unique for each patient and linked to the subject ID; it is not interchangeable. In addition, there is a status

section, which determines if the patient has registered. When the patient has registered, the status will change from "invited" to "registered."

d. Patient Compliance

The patient data imports directly from a device into the Rave database. There are no documents to audit. The electronic responses are the source documentation.

e. Security

All data are encrypted on the device (128 bit on file +https transfer) and the app requires a user to have a username and password. If the user is idle for too long (5 minute inactivity time), the app will time out and the user will need to log in again.

The data will only reside on the device for a short period of time. Once the user clicks "submit," the data is securely transferred over https between the device and internal relay. No identifying information is stored in iMedidata (only email address is stored).

The Patient information (email/password) does not reside in Medidata Rave EDC and the patient accounts are hidden in iMedidata from sites and sponsors in the Patient Cloud Relay.

The ePRO application is Part 11 compliant and acts as a gateway between device and Medidata Clinical Cloud (MCC).

Messages and information communicated to and from the Patient Cloud are encrypted and therefore this information cannot be read if intercepted while in transit.

f. Checklist for Activities Prior to Consenting a Patient

- ☐ Contact the Data Manager to request Patient Cloud access
- ☐ Accept study invitation at imedidata.com
    - Note: you must be rostered and have received an invitation
- ☐ Complete required eLearning
- ☐ Verify Patient Cloud app is up-to-date
- ☐ Review Quick Reference Guides

APPENDIX XVI: SEMI-STRUCTURED QUALITATIVE INTERVIEW GUIDE FOR PROCESS EVALUATION

I. Introduction

Hello, thank you for participating in this interview. My name is ____ and I'll be interviewing you today. With me is ____, who will take notes on our discussions.

Today we'd like to talk about your experiences with the GET SET program. It is important that you feel comfortable sharing your opinions on how this program worked- either good or bad- so that we can continue to make improvements.

We will be recording our discussion, because we want to make sure we don't miss anything you share with us. We want to assure you that all of your comments are confidential and that nothing you say will be connected with your name or shared with any of your colleagues. Participation in this study is completely voluntary and you may stop at any time or skip any question you do not wish to answer.

Do you have any questions before we begin?

General questions:

1. **Do you feel GET SET was implemented according to the implementation plan?**
   o [If Yes] Can you describe this?
   o [If No] Why not?

Intervention characteristics:

2. **How does GET SET compare to other options for increasing ET adherence?**
   -What are its advantages and disadvantages compared to other options for increasing adherence?

3. **How complicated do you feel GET SET was for you? For participating patients?**
   - [Please consider the following aspects of the intervention: duration, scope, intricacy and number of steps involved and whether the intervention reflects a clear departure from previous practices.]

4. **What is your perception of the quality of the supporting materials, packaging, and bundling of GET SET for implementation?**
   - [If needed, prompt what they did or did not like about it]

Outer setting:

5. **How well do you think GET SET will meet the needs of the individuals who participate?**
   - [In what ways will the intervention meet their needs? E.g. improved access to services? Reduced wait times? Help with self-management? Reduced travel time and expense?]
   6b. What barriers will the individuals face to participating in the intervention?

Organization characteristics [Think about your site as whole]

6. **How did your relationship with the study coordinator and other members of the study team effect the implementation of GET SET?**
   - What changes would you make in future?

7. **Was your training adequate for implementing GET SET?**

Final Question:

8. **Is there anything else you'd like to add about what worked or did not work about the implementation of GET SET?**

Thank you so much for your time!

APPENDIX XVII: TEXT MESSAGE REMINDER (TMR) INTERVENTION EVALUATION

1. I was able to recognize that the text message reminders and monthly questions were coming from the GETSET Study when I received them. (In other words, you didn't think they were "spam" or "junk" messages.]

a. Strongly Agree
b. Agree
c. Disagree
d. Strongly Disagree

2. I was able to complete the monthly questions with little or no difficulty.

a. Strongly Agree
b. Agree
c. Disagree
d. Strongly Disagree

3. The number of months (9) that I received the daily texts to take my ET medication was:

a. Just about right
b. Too long → How many months would you have preferred to receive the text message reminders instead? ______ months
c. Too short → How many more months would you have liked to have received the daily text message reminders to take your ET medication? _____

4. Do you have any other comments or feedback about these ET text reminders that you would like to share with us?

a. No
b. Yes → Comment Box: ____________

APPENDIX XVIII: IPHONE RECIPIENT EVALUATION SURVEY

(only for participants randomized to TMR arm AND who received a study-provided iPhone)

Thank you for participating in the GETSET Study. Because you were randomized to the text message reminder group and received an iPhone with Verizon Wireless service in order to participate in this study, we are interested in learning about your experience with the iPhone over the past year. Please complete the survey below. This survey should take about 5 minutes to complete.

For the following items, please indicate how much you agree or disagree with each statement:

	**Strongly agree**	**Agree**	**Disagree**	**Strongly disagree**
1. The phone was easy to use.				
2. I received enough training from study staff to use my new phone.				
3. Phone service from Verizon was fast and reliable.				
4. The phone helped me communicate better with my loved ones or support network.				
5. Having this phone has made a positive impact on my everyday life.				

6. Do you plan to get phone service and a data plan for your iPhone from Verizon or another carrier when your 12 months of phone service has ended?

a. Yes
b. No

7. Regardless of whether you get a cell phone plan from Verizon or another carrier, do you plan to continue using your iPhone after you are done participating in this study (for example, using the phone in a place where Wi-Fi is available to access the internet or using the camera)?

a. Yes
b. No→

a. Why not? ____

8. Do you have any additional comments or feedback about your experience using the iPhone in this study that you would like to share with us?

a. No
b. Yes→ please specify: ____

9. Please provide us with the name and phone number of a family member or friend who will know how to reach you over the next 12 months. The reason we are asking for this information is that you will be contacted to complete surveys using the ePRO app at 6 and 12 months from now. We will need to be able to reach you if you do not have phone service at this time. The person you list below will not be contacted at any time, unless we are unable to reach you.

Name: ______________________ ____________________

First Last

Relationship to you: ____________________________ (Example: friend, sister, daughter)

Phone Number: (   ) - _________ - ______________

Thank you for completing this survey. Please contact the TMR team at GETSETtext@osumc.edu if you have any questions.

APPENDIX XIX: IPHONE PARTICIPANT RESPONSIBILITIES AND AGREEMENT FORM

iPhone Participant Responsibilities and Agreement Form

You are being provided with an iPhone from The Ohio State University to participate in this study. These iPhones will include a cell phone service and data plan provided to you free of charge from Verizon Wireless with unlimited internet data.

The Get Set study will pay for 12 months of cell phone service. This does **not** include any apps or additional services, subscriptions, downloads or media that have charges. All charges must be linked to a personal account and/or card. At the end of the 12-month period, you may keep the iPhone for your own use, if you complete the study requirements listed below. However, you will need to get a service contract from Verizon Wireless or another carrier if you want to use your phone to call or text. Even if you do not decide to get another cell phone service contract, you can still use your phone using Wi-Fi in a public place or at a friend or family member's home.

<u>In order to receive an iPhone, we ask that you agree to the following</u>:

1. Complete surveys on your iPhone using the ePRO app about your quality of life and medication use at the time you are enrolled in the study, and then at 3, 6, and 12 months.

2. Agree to receive and respond to text messages and phone calls from The Ohio State University staff regarding your smart phone through the end of your 12-month service contract.

3. Provide the name and phone number of a family member or friend who will know how to contact you, if for some reason we are not able to reach you during the study period. We will only contact this person in the unlikely event that we are unable to reach you.

4. Complete a survey about your experience using the iPhone at 12 months, before your 12-month service contract ends.

5. Receive daily text message reminders to take your endocrine therapy medicine for 9 months. (Text message reminder participants only)

6. Answer questions every month for 9 months on your smart phone asking you about your endocrine therapy use during the past 7 days. (Text message reminder participants only)

7. Complete an end of the text message intervention survey at 9 months. (Text message reminder participants only.)

8. Complete 5 sessions of counseling on your iPhone about taking your endocrine therapy. (Telephone counseling group only)

Study participants who receive an iPhone, but do not perform the study activities (surveys or telephone counseling sessions) will be contacted by study staff. Study staff reserve the right to discontinue the phone service plan early if you are no longer participating in the study.

End of the 12-month contract Period:
Close to the end of your 12-month iPhone Verizon contract period, you will be contacted by Ohio State University staff to help you obtain a new service contract, if desired. Staff will explain options to you if you want to keep your phone number, add your phone to someone else's phone plan, or switch to a new phone company.

Completing the 18- and 24- month ePro Surveys
Just a reminder that we are asking all study participants to complete surveys using the ePRO app at 18 and 24 months from the time they began the study. We are only able to pay the phone charges for the first 12 months, when the active parts of the study (text messaging reminder and the counseling sessions) are being completed. However, we still would like participants receiving study iPhones to complete the 18- and 24-month surveys. You will still be able to use the apps on your phone at places where Wi-Fi is available – even if you do not choose to set up the phone on another calling plan.

Who to contact for iPhone assistance?
If at any time you have questions about your iPhone or need a phone repair, please call the Project Coordinator at Ohio State University for assistance at 614-293-8006 or GETSETtext@osumc.edu. Please do not contact the medical center where you received the iPhone when you enrolled in the study. The Ohio State University has service contracts for all study phones, and will be happy to help you with any issues that you may have.

I understand and agree to the requirements for receiving an iPhone for this research study.

______________________________________ ____________________
Participant Signature Date

________-_________-_____________________
iPhone Number Assigned

Witness name:

____

Site Coordinators – after obtaining signature, please fax to OSU study coordinator at (614) 293-2654.

APPENDIX XX: A191901 RECRUITMENT SCRIPTS

A191901 General Script for Telephone Verbal Recruitment

Hi (*insert name of patient*),

This is name of caller (consenter) from (*insert name of site*). I am calling you about a research study called, "Optimizing Endocrine Therapy Through Motivational Interviewing and Text Interventions." A member of your clinical care team (*insert name of provider*) mentioned that you might be interested in learning more about this study.

This study is for women who are about to begin taking endocrine therapy medication for their breast cancer. We are doing this study because we want to find out if using either text message reminders or telephone-based counseling, or both is better or worse than the usual approach for making sure that women take their ET medication as prescribed.

If you decide to take part in this study, you will be assigned to one of four study groups. Each group offers basic education about endocrine therapy and healthy living after breast cancer. Additionally, some groups will get extra support programs, such as daily text message reminders and/or telephone counseling sessions.

If you are interested in participating, you will need to have a smart phone device. However, if you do not already have a smart phone, the study might be able to provide you one.

The next step is to review and sign the consent forms which can be done at your next standard of care appointment. We will go over the consent in detail and you will be given a physical hard copy of your signed forms for your records.

Do you have any questions? (answer them appropriately, if not, move on with script)

If you have any questions or concerns, you can call me at (*insert site contact number*) or email me at ___site staff email address___.

Thanks and have a nice day!

*** Note date and time of today's call and if a telephone consent is needed, collect the information below (Date/Time of appointment, In-person/Telephone Consent). Additionally, obtain the mailing address of the potential subject and send them the ICFs and HIPAA prior to consent.***

Date of today's call: ________________ Time of today's call: ______________

Mailing Address of potential subject:_____________________________________

Date/Time of Appointment for Informed Consent: __________________________

☐ In-person Consent Visit ☐ Telephone Consent

A191901 Text for Email/Post Recruitment

Dear Mrs/Ms ___(name)

My name is **name of study staff** from (*insert name of site*). I am contacting you about a research study called Guiding Endocrine Therapy Success through Empowerment and Technology, or GET SET. A member of your clinical care team (*insert name of provider*) mentioned that you might be interested in learning more about this study.

The GET SET study is for women who are about to begin taking endocrine therapy medication for their breast cancer. We are doing this study because we want to find out if using either text message reminders or telephone-based counseling, or both is better or worse than the usual approach for making sure that women take their ET medication as prescribed.

If you decide to take part in this study, you will be assigned to one of four study groups. Each group offers basic education about endocrine therapy, and healthy living after breast cancer. Additionally, some groups will get extra support programs, such as daily text message reminders and/or telephone counseling sessions.

If you are interested in participating, you will need to have a smart phone device. However, if you do not already have a smart phone, the study might be able to provide you one.

The next step is to review and sign the consent forms which can be done at your next standard of care appointment or by phone. We will go over the consent in detail and you will be given a physical hard copy of your signed forms for your records.

I will be calling you in the next couple of weeks to see if you are interested in hearing more about the GET SET study and sending you consent forms for us to discuss. If you have any questions or are interested in participating in this study, you can also call me at (*insert site contact number*) or email me at __site staff email address__.

Thanks and have a nice day!

Kind Regards,

Site Staff Name and Credentials

*** Note date of email/snail mail correspondence in study records, as well as any follow-up correspondence. If potential subject chooses to move forward with informed consent procedures, indicate the appropriate mailing address, date/time of appointment for informed consent, and mode of informed consent. Send the ICFs and HIPAA prior to the consent date listed below***

Date of initial correspondence: _________________

Date of follow-up correspondence: _______________

Mailing Address of potential subject:_______________________________________

Date/Time of Appointment for Informed Consent: ____________________________

☐ In-person Consent Visit ☐ Telephone Consent

APPENDIX XXI: TELEPHONE CONSENT PROCEDURE & SCRIPT

Sites should follow the NCI's CIRB Remote Consenting Procedures to implement a remote consenting process. Please consult CIRB Remote Consent Procedures prior to remote consenting participants.

Telephone Consenting Script

Introduction

Hello Mrs./Ms. [patient's last name], my name is [name of individual delegated to obtain informed consent].

- I work with [name of PI], who is working with the Alliance for Clinical Trials in Oncology to do a research study called "Guiding Endocrine Therapy through Empowerment and Technology." This study is also known as GET SET.
- I am calling you because either you have expressed interest in participating in GET SET or your provider has confirmed you may be eligible for the study.
- You should have received a copy of the informed consent form document in advance of this conversation to allow for time to review study details and to make an informed decision regarding your voluntary participation.[1]
- Would you like to continue to discuss the GET SET trial?
    - *If patient declines: Thank the patient for her time and consideration and end the call.*
    - *If patient accepts: Continue with the consent discussion.*

Site staff goes through the specific study and consent form, including HIPAA[2]

- Do you have any questions about anything I've told you or anything you see in the consent forms? (answer questions)
- Remember, this research study is completely voluntary and you can withdraw at any time. Are you interested in participating?[3]
- Okay, now I would like you to sign the consent forms while I still have you on the phone. The line for you to sign is on the last page of each form. There is a place for you to sign your name, a place for you to print your name, and a place for you to put the date. Please fill out each one. Done?
- Now, let's go over how you are going to return these forms to me. What's the best way for you to send them back? (I'm going to mail/fax/email them). Good, I'll look forward to getting them from you. Do you have the (*insert site fax number, address, email address*)?
- Once we have received the consent forms back from you, we will call you back to go through the Patient Contact Information Form for the study and schedule your baseline visit.
- If you have any questions or concerns in the meantime, you can call me at (*insert site staff phone number*) or email me at (*insert site staff email*).

Notes:

1. If the patient does not have the consent forms, check to see if she knows where they are and can get them, or arrange to re-send them if lost and schedule another time.
2. Please consult the CIRB Remote Consent Procedures to see the requirements of a witness.
3. If the person states they are not interested, thank them for their time and let them know that they can call you (the caller/consenter) if they have any further questions or concerns.
